# Modelling the impact of screening for chlamydia and gonorrhoea in youth and other high-prevalence groups in a resource-limited setting

**DOI:** 10.1007/s00038-020-01351-0

**Published:** 2020-04-09

**Authors:** Rachel T. Esra, Leigh F. Johnson

**Affiliations:** School of Public Health and Family Medicine, University of Cape Town, Cape Town, South Africa

**Keywords:** STI screening, Mathematical model, Chlamydia, Gonorrhoea, South Africa

## Abstract

**Objectives:**

Modelling the potential impact of screening for chlamydia and gonorrhoea in youth and other populations in a resource-limited setting.

**Methods:**

We extended an agent-based model of heterosexual chlamydia and gonorrhoea transmission in South Africa to investigate the impact of screening strategies in key populations including youth, patients in HIV care, pregnant women and female sex workers (FSWs). Additionally, we compared the modelled impact of a standardised screening programme to results obtained from other published mathematical models of chlamydia screening.

**Results:**

All strategies resulted in reductions in general and targeted population chlamydia and gonorrhoea transmission. Opportunistic screening of patients in youth and HIV care was shown to be the most effective, and FSW screening was shown to be the most efficient strategy. Differences between models could be attributed to differences in the modelled heterogeneity in sexual behaviour as well as differences in assumptions about immunity following chlamydia recovery.

**Conclusions:**

Taking modelling assumptions into account, opportunistic chlamydia and gonorrhoea screening of youth and those in HIV care represents a viable intervention for reducing sexually transmitted infections in the South African population.

**Electronic supplementary material:**

The online version of this article (10.1007/s00038-020-01351-0) contains supplementary material, which is available to authorised users.

## Introduction

Curable bacterial sexually transmitted infections (STIs) such as *Chlamydia trachomatis* (chlamydia) and *Neisseria gonorrhoea* (gonorrhoea) were responsible for 214 million new infections in 2016 (World Health Organisation 2018). If undiagnosed or untreated, chlamydia and gonorrhoea can result in serious reproductive problems in women (Menon et al. 2015). This presents a major global health challenge, particularly in youth, with 14–25-year-olds in the USA accounting for 65% of chlamydia infections and 53% of gonorrhoea infections (Centers for Disease Control and Prevention 2015). Barriers to accessing healthcare, multiple concurrent sexual partners and limited knowledge of sexual health contribute to increased risk of STIs during adolescence (Centre for Disease Control and Prevention 2013). Additionally, cervical ectopy during adolescence may place young women at an increased risk of bacterial STI acquisition (Lee et al. 2006).

In high-income countries, disproportionately high rates of bacterial STIs in those under the age of 25 have resulted in youth-directed opportunistic chlamydia screening programmes to identify and treat STI cases and interrupt the spread of infection through the population. Despite this, notifications of curable bacterial STIs have risen consistently (Low 2007), resulting in questions about the quality, or absence, of the clinical evidence currently informing STI screening strategies (Low 2007). Due to the lack of high-quality empirical data on the effectiveness of opportunistic STI screening (Low et al. 2006, 2016), dynamic mathematical modelling has been used to help understand the levels of coverage and frequency of screening required to reduce STIs levels (Turner et al. 2006; Kretzschmar et al. 2009; Low et al. 2009). Modelling studies of the impact of STI screening in high-income countries have resulted in substantially different conclusions regarding the impact of chlamydia screening in youth (Althaus et al. 2011), and the reasons for these model discrepancies are not well understood.

Globally, STI screening guidelines that have been developed focused on chlamydia specifically and only in the USA is gonorrhoea screening indicated for high-risk groups (Centre for Disease Control 2015). This reflects the STI disease burden in high-income countries, in which the prevalence of gonorrhoea is negligible compared to the prevalence of chlamydia (Newman et al. 2015). In developing countries with limited resources for diagnosis and treatment, STI screening is not generally recommended and most STIs are treated syndromically. With up to 60–70% of chlamydial and gonococcal infections remaining asymptomatic (Korenromp et al. 2002; Farley et al. 2003), there is a strong appreciation of the limitations of syndromic management and few modelling studies have been conducted to assess the potential impact of opportunistic STI screening programmes in developing countries (Vickerman et al. 2006, 2010). Historically, limited access to laboratory diagnostic services has stood as a barrier to STI testing, and the feasibility of using rapid point-of-care (POC) STI tests to deliver on-site STI diagnosis in resource-poor settings has yet to be determined.

South Africa is an upper-middle income country with some of the highest STI prevalence levels compared to other African countries and global averages (Newman et al. 2015). There are no nationally representative data on the population-wide prevalence of bacterial STIs in South Africa, and clinical studies employing laboratory testing of STIs report varying levels of STI prevalence in different regions and sentinel populations. Consistent with global trends, bacterial STI rates in young South African females are generally higher than their male counterparts (Pettifor et al. 2005; O’Leary et al. 2015). The prevalence of chlamydia and gonorrhoea in South African women in 2005 was estimated to be 10.1% and 4.4%, respectively (Johnson et al. 2012a) compared to levels of 3.0% and 0.3%, respectively, in high-income countries (Newman et al. 2015). Reports of high burdens of asymptomatic STIs in South African women (Mlisana et al. 2012; Peters et al. 2014) bring into question the adequacy of the syndromic approach to STI control that is currently being implemented. South Africa also has a relatively high prevalence of concurrent sexual partnerships, which have been shown to be important in sustaining the relatively high incidence of gonorrhoea and chlamydia seen in the country (Johnson and Geffen 2016), and large partner age differences are also relatively common when compared to high-income countries (Wellings et al. 2006). In a country like South Africa, where the prevalence of gonorrhoea is almost half the prevalence of chlamydia (Johnson et al. 2012a), it is unclear whether the screening approach should be modified to include gonorrhoea as well as chlamydia or to include populations other than youth, nor is it obvious whether youth STI screening would have a different effect to that estimated in high-income settings.

We therefore aim to model the potential impact of chlamydia and gonorrhoea screening in South African youth and to compare this with other potential screening strategies. We also aim to build a better understanding of the factors that account for differences in model predictions by comparing our model estimates of the impact of youth chlamydia screening against those of other models applied in high-income settings. This study uses a previously developed agent-based model of STIs in South Africa to estimate the effectiveness and efficiency of opportunistic STI screening in a South African setting. We investigate the impact of opportunistic screening strategies targeting high-risk groups including youth, female sex workers (FSWs), pregnant women and those in human immunodeficiency virus (HIV) care. Additionally, we compare the modelled impact of a standardised screening programme to results obtained from other published mathematical models of chlamydia screening.

## Methods

We extended a previously developed model of heterosexual HIV and STI transmission in South Africa (Johnson and Geffen 2016). MicroCOSM (Microsimulation for the Control of South African Morbidity and Mortality) is an agent-based model simulating the behaviour and disease profile of a nationally representative sample of South Africans; the initial population size in 1985, at the start of the simulation, is 20,000, and the model is projected to 2028. A detailed description of the model specification and sexual behaviour parameterisation can be found in Electronic Supplementary Material 2. Briefly, the population is stratified by sexual risk behaviour. High-risk individuals have a propensity for concurrency and commercial sex, and low-risk individuals engage only in monogamous relationships. Both high- and low-risk individuals can engage in either short-term non-cohabitating or long-term cohabiting relationships. The choice of modelling approach was informed by previous work demonstrating more realistic results using stochastic network-based as opposed to frequency-dependent modelling (Johnson and Geffen 2016).

We have introduced the components of a hypothetical opportunistic screening intervention while keeping parameters describing the transmission, duration and syndromic management of STIs the same as in the original model (Johnson and Geffen 2016). For the purpose of this study, only chlamydia, gonorrhoea and HIV transmission are simulated. Assumptions are made regarding probabilities of transmission per act of unprotected sex, proportions of cases that become symptomatic and the average duration of infection in the absence of treatment, as has been previously described (Johnson and Geffen 2016). The model has been fitted to South African STI prevalence data, and for each STI a set of 100 parameter combinations has been identified that yield the best model fit to South African STI prevalence data (Electronic Supplementary Material 2, Tables S8 and S9). All figures were created using GraphPad Prism version 6 for MacBook (GraphPad software, La Jolla California USA).

### STI screening

In each scenario, a hypothetical ten-year screening programme is simulated, starting in 2018. The screening and testing strategies are detailed in Electronic Supplementary Materials 1 and 2. Individuals are screened simultaneously for chlamydia and gonorrhoea with a POC test assumed to have the same sensitivity as the GeneXpert sensitivity for urine samples (97.4% in females and 97.5% in males for CT, 95.6% in females and 98.0% in males for NG) (Gaydos et al. 2013). We compared the population-wide impact of 4 different opportunistic screening strategies targeting youth (aged 15–24), FSWs, pregnant women and patients in HIV care (aged > 14). A detailed description of the specification of these target groups is provided in Electronic Supplementary Material 1. For each strategy, we investigated the population impact of targeted screening with and without partner notification. Women engaging in sex work are assumed not to form short-term or long-term relationships during the periods in which they are active as sex workers. Due to this, partner notification could not be simulated in the FSW population. In partner notification scenarios, if screening produces a positive result in the index case, both primary and secondary partners are screened with a 50% probability (Electronic Supplementary Material 1, Table S4). The assumed probability of cure in treated individuals is the same in index cases and screened partners. The effectiveness of different screening programmes was assessed by comparing the cumulative incidence of new STI cases over the 10-year screening programme and the reduction in STI prevalence after 10 years of screening, relative to a base scenario in which no screening takes place.

### Specification of modelling assumptions

In the case of antenatal screening, a single screening probability was applied to all pregnant women based on the current rates of antenatal syphilis screening in South Africa (Dinh et al. 2013). For the other screening strategies, the annual rate of STI screening was calculated as the product of the probability of healthcare utilisation, STI screening acceptability and screening coverage (the probability of being offered an STI test). STI screening parameters were specified separately for each population, as summarised in Table 1.

**Table 1:** Sexually transmitted infection (STI) screening model parameters

Parameter	Females	Males	Source^a^
Assumed rate of screening in populations for which screening rates are unknown			
Screening coverage^b^	0.80	0.80	
Annual healthcare utilisation rate			
Youth (15–24)	0.48	0.32	GHS (South Africa)
FSWs	0.50	N/A	Literature (South Africa)
HIV care^c^	0.90	0.90	Literature (South Africa)
Screening acceptability			
Youth (15–24)	0.60	0.60	Literature^e^
FSWs	0.80	N/A	Literature^e^
HIV care	1.00	1.00	Literature^e^
Total annual screening rate			
Youth (15–24)	0.23	0.15	The total annual screening rate was calculated as the product of heath care utilisation, screening acceptability and screening coverage
FSWs	0.32	N/A	
HIV care	0.72	0.72	
ANC	0.71	N/A	Literature (South Africa)
Partner notification^d^			
Proportion of partners screened	0.50	0.50	Literature^e^

*ANC* antenatal care, *FSW* female sex worker, *GHS* General Household Survey

^a^A full references of the sources on which these assumptions were based on are included in Supplemental Digital Content 1 Table S2

^b^Probability of being offered an STI test

^c^HIV-positive individuals, above the age of 14 and either on ART or in the symptomatic stages of HIV disease (WHO stage III or IV), are eligible for annual STI screening

^d^Only applicable in scenarios where partner notification is implemented

^e^In the cases where there are no current data on these behaviours in the South African population, data sources from other countries, where screening for chlamydia and gonorrhoea has been implemented, have been considered

### Standardised screening programme

Previous studies have compared three published models of opportunistic chlamydia screening, simulating the same hypothetical youth-directed screening strategy in European settings (Kretzschmar et al. 2009; Althaus et al. 2011). We adjusted our model in order to simulate the same screening strategy and compared the outcomes to the above-mentioned studies. In order to be comparable, the following adjustments were made: 16–24-year-olds were screened with an annual probability of 35%, and partner notification resulted in 45% of partners successfully screened and treated (Kretzschmar et al. 2009). We additionally calculated the Gini coefficient, a measure of the distribution of chlamydia infections among individuals with different levels of sexual activity (Althaus et al. 2011). A Gini coefficient of 0 represents a situation where the prevalence of chlamydia infections is equal across all sexual behaviour groups. A Gini coefficient of 1 represents a situation of maximum inequality where chlamydia infections occur only in the group with the highest rate of sexual contacts.

## Results

### Population-level impact of targeted STI screening

**Fig. 1:**
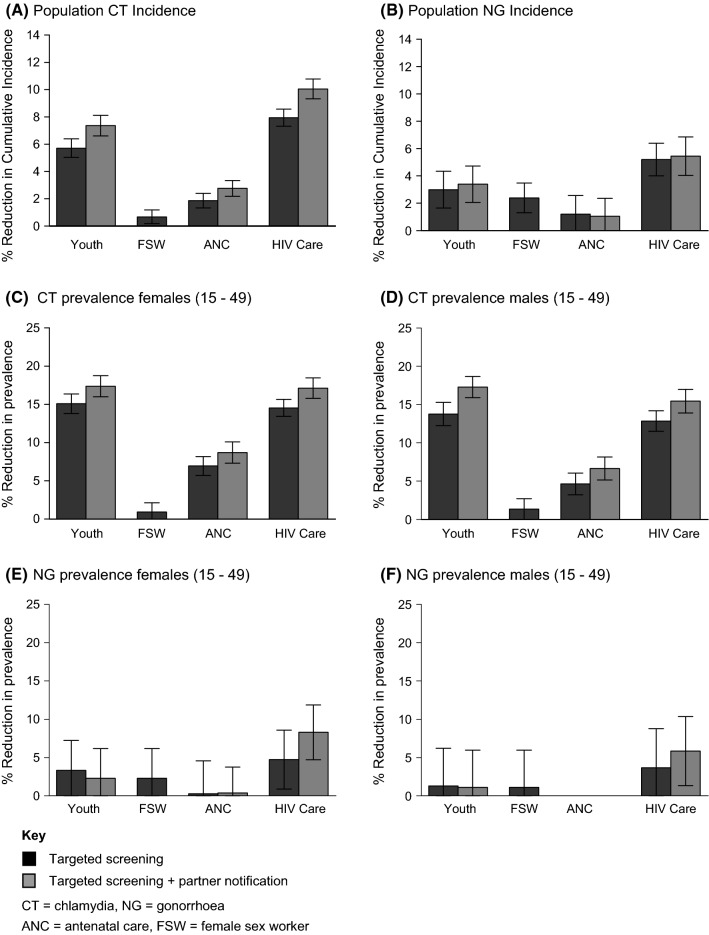
Comparison of estimated population-level effect of targeting youth, female sex workers (FSW), antenatal care (ANC) and those in HIV care for sexually transmitted infection (STI) screenings. Bars represent mean reductions in the incidence (A + B) and prevalence (C-F) of chlamydia and gonorrhoea after the implementation of a targeted STI screening programmes (errors bars represent 95% confidence intervals). The reduction in incidence and prevalence is calculated relative to the level that would be expected in the absence of any screening programme. Lower limits of the 95% CI are not shown if they extend past zero. The means and 95% CIs are calculated from the range of model outputs generated when the 100 best-fitting parameter combinations are entered into each model. Estimated trends for South Africa from 2018 to 2028

Figure 1 illustrates the predicted change in population-level chlamydia and gonorrhoea incidence and prevalence after different screening strategies, relative to the base scenario. For both chlamydia and gonorrhoea incidence, HIV care and youth screening resulted in the largest decrease in cumulative incidence over the 10 years of the screening period, followed by antenatal care (ANC) and FSW screening (Electronic Supplementary Material 1, Table S10). In terms of chlamydia prevalence, youth screening resulted in the largest population-wide reductions, followed by screening those in HIV care, ANC and FSWs (Fig. 1, Tables S10 and S11). Significant reductions in population-level gonorrhoea prevalence were only seen for HIV care screening (Fig. 1). Partner notification resulted in significantly fewer incident and prevalent cases of chlamydia for both men and women for all screening scenarios in comparison with screening without partner notification (Fig. 1). Across all scenarios, partner notification did not significantly reduce the number of incident or prevalent cases of gonorrhoea. Screening FSWs was the most efficient screening strategy resulting in significantly more STI cases averted per screening test than any of the other screening strategies (Table 2).

**Table 2:** Estimated number of new sexually transmitted infection (STI) cases averted (in the general population) per 100 individual STI tests

Screening strategy	Chlamydia	Gonorrhoea
Adolescent	5.7 (5.1–6.3)	5.2 (3.1–7.2)
Adolescent PN	6.9 (6.2–7.5)	5.9 (4–7.8)
FSW	45 (13–78)	248 (140–357)
ANC	5.5 (4–7)	8.2 (1.5–14.9)
ANC PN	7.5 (6–9)	6.5 (1.1–11.9)
HIV care	5.2 (4.8–5.6)	5.5 (4.3–6.7)
HIV care PN	6.3 (5.8–6.7)	5.8 (4.5–7.1)

Number of STI cases averted per single dual screening test was calculated as the cumulative reduction in incident STI cases divided by the total number of screening tests over the 10-year screening period

The means and 95% CIs are calculated from the range of model outputs generated when the 100 best-fitting parameter combinations are entered into each model. Estimated trends for South Africa from 2018 to 2028

*ANC* antenatal care, *ART* antiretroviral treatment, *FSW* female sex worker, *PN* partner notification

### Key population impact of targeted STI screening

Youth-directed screening resulted in large decreases of chlamydia prevalence in both female and male youth and modest reductions in gonorrhoea prevalence (Electronic Supplementary Material 1, Table S13). While HIV care and ANC-directed screening resulted in the reduction of chlamydia prevalence in youth, these effects were much smaller than those estimated for youth-targeted screening (Electronic Supplementary Material 1, Table S13). Additionally, FSW-directed screening was shown to have no impact on STI prevalence in the youth population (Electronic Supplementary Material 1, Table S13). Significant reductions in chlamydia prevalence were predicted in ART patients, pregnant women and FSWs after respective targeted screening strategies (Tables 3 and S12). Significant reductions in gonorrhoea prevalence after targeted screening were predicted in all targeted populations with the exception of male youth and pregnant women (Tables 3 and S12). Partner notification resulted in significantly fewer prevalent cases of chlamydia in all key populations but had no significant impact on gonorrhoea prevalence in comparison with targeted screening without partner notification (Tables 3 and S12).

**Table 3:** Estimated sexually transmitted infection (STI) prevalence in targeted populations in 2028, after the implementation of a 10-year targeted screening programme

Scenario	Chlamydia		Gonorrhoea	
	Females	Males	Females	Males
Projected prevalence in 2018 (prior to the implementation of screening)				
General population (15–49 years)	9.2 (8.99–9.38)	6.73 (6.53–6.92)	3.08 (2.89–3.27)	1.57 (1.46–1.69)
Youth (15–24 years)	12.62 (12.32–12.92)	6.69 (6.46–6.93)	4.01 (3.77–4.25)	6.69 (6.46–6.93)
FSWs	15.33 (14.66–15.99)		24.22 (23.31–25.14)	
ANC	12.38 (12.13–12.63)		4.30 (4.03–4.56)	
HIV care	9.60 (9.28–9.92)	9.19 (8.84–9.55)	3.77 (3.50–4.03)	2.49 (2.28–2.71)
*Projected prevalence in 2028 (after a 10-year opportunistic screening programme)*				
Prevalence in the general population (aged 15 to 49 years)				
Base scenario^a^	8.87 (8.69–9.05)	6.56 (6.39–6.74)	2.97 (2.79–3.15)	1.51 (1.40–1.63)
Youth screening	7.52 (7.35–7.69)	5.66 (5.48–5.84)	2.82 (2.65–3.00)	1.45 (1.34–1.56)
Youth screening + PN	7.33 (7.14–7.52)	5.44 (5.26–5.62)	2.85 (2.67–3.02)	1.46 (1.35–1.57)
FSW screening	8.77 (8.59–8.96)	6.47 (6.29–6.65)	2.88 (2.71–3.06)	1.48 (1.36–1.60)
ANC screening	8.25 (8.06–8.44)	6.26 (6.07–6.46)	2.89 (2.72–3.07)	1.51 (1.40–1.63)
ANC screening + PN	8.10 (7.90–8.30)	6.13 (5.94–6.33)	2.90 (2.74–3.06)	1.47 (1.36–1.57)
HIV care screening	7.57 (7.40–7.74)	5.72 (5.55–5.88)	2.77 (2.61–2.94)	1.41 (1.31–1.52)
HIV care screening + PN	7.35 (7.16–7.53)	5.55 (5.37–5.73)	2.68 (2.52–2.84)	1.38 (1.28–1.49)
Prevalence in youth (aged 15 to 24 years)				
Base scenario^a^	12.27 (11.96–12.57)	6.35 (6.59–6.1)	3.91 (3.66–4.17)	1.52 (1.40–1.64)
Youth screening	9.10 (8.82–9.38)	4.88 (4.65–5.10)	3.53 (3.31–3.76)	1.41 (1.30–1.51)
Youth screening + PN	8.49 (8.20–8.78)	4.34 (4.15–4.53)	3.49 (3.27–3.70)	1.41 (1.30–1.52)
Prevalence in FSWs				
Base scenario^a^	15.19 (14.51–15.88)		24.15 (23.2–25.09)	
FSW screening	13.84 (13.22–14.46)		22.59 (21.69–23.49)	
Prevalence in pregnant women				
Base scenario^a^	12.15 (11.92–12.38)		4.27 (4.01–4.53)	
ANC screening	11.28 (11.02–11.53)		4.16 (3.91–4.41)	
ANC screening + PN	11.06 (10.79–11.33)		4.14 (3.92–4.37)	
Prevalence in patients linked to HIV care				
Base scenario^a^	8.37 (8.10–8.65)	8.24 (7.94–8.55)	2.91 (2.71–3.11)	1.95 (1.79–2,12)
HIV care screening	3.94 (3.76–4.12)	3.93 (3.71–4.14)	2.21 (2.04–2.38)	1.50 (1.36–1.64)
HIV care screening + PN	3.63 (3.46–3.79)	3.70 (3.52–3.88)	2.09 (1.93–2.26)	1.44 (1.30–1.57)

The means and 95% CIs are calculated from the range of model outputs generated when the 100 best-fitting parameter combinations are entered into each model. The hypothetical implementation of STI screening programmes is simulated as being initiated in 2018. Estimated trends for South Africa from 2018 to 2028

*ANC* antenatal care, *ART* antiretroviral treatment, *FSW* female sex worker, *PN* partner notification

^a^Base scenario prevalence estimates are the prevalence levels that would be expected in the absence of any screening intervention

### Sensitivity analysis

We calculated correlation coefficients between the reduction in chlamydia and gonorrhoea incidence and prevalence in the general population and the 100 best-fitting parameter combinations. The probability of chlamydia transmission per sex act was positively correlated with the reduction in chlamydia incidence and prevalence due to screening (Electronic Supplementary Material 1, Table S8). Additionally, the duration of asymptomatic chlamydial infection and the duration of chlamydial immunity were negatively associated with the reduction in chlamydia incidence and prevalence (Electronic Supplementary Material 1, Table S8). The same relationships were not observed between gonorrhoea parameters and reductions in gonorrhoea incidence and prevalence (Electronic Supplementary Material 1, Table S9).

### Standardised screening programme

**Fig. 2:**
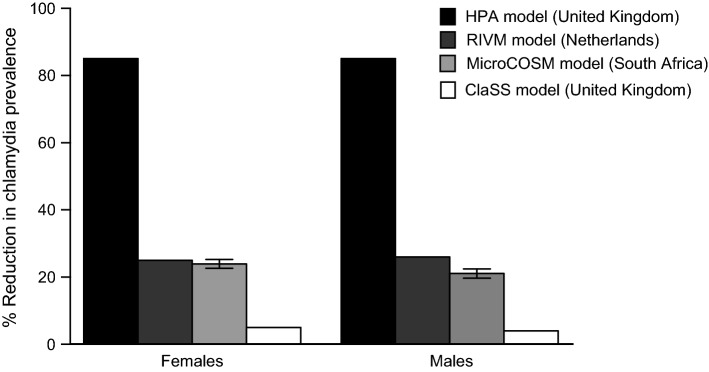
Comparison of modelled effects of a standardised chlamydia screening programme on chlamydia prevalence, as estimated by different mathematical models. Bars represent the percentage reduction in chlamydia prevalence in 16–44-year-olds after 10 years of chlamydia screening in women only (errors bars represent 95% confidence intervals). For the MicroCOSM model, the means and 95% CIs are calculated from the range of model outputs generated when the 100 best-fitting parameter combinations are entered into the model. Estimated trends for UK, Netherlands and South Africa after a hypothetical 10-year screening programme. *HPA* Health Protection Agency, UK, *RIVM* National Institute for Public Health and the Environment, Netherlands, *MicroCOSM* Microsimulation for the Control of South African Morbidity and Mortality, South Africa, *ClaSS* The Chlamydia Screening Studies, UK

A comparison of MicroCOSM and the three previously published models of chlamydia screening is summarised in Electronic Supplementary Material 1 tables S15 and S16. Compared to other models, prescreening chlamydia prevalence in MicroCOSM was substantially higher, at 10.2% (95% CI 9.9–10.4%) for females and 7.4% (95% CI 7.2–7.6%) for males (Electronic Supplementary Material 1, Table S14). The MicroCOSM model estimated the impact of the standardised chlamydia screening programme to be similar to that estimated by the RIVM model, but substantially less than that estimated by the HPA model and substantially more than that estimated by the ClaSS model (Fig. 2). We calculated the Gini coefficients for chlamydia and gonorrhoea to be 0.37 and 0.40, respectively.

## Discussion

High rates of bacterial STIs in youth have directed STI screening strategies in the USA, UK, Sweden and Australia. Based on estimates of low levels of health-seeking behaviours in South African youth, we assumed that only 23% of female and 15% of male youths would be opportunistically screened per annum in a South African setting (Statistics South Africa 2017). This study demonstrates that even at such low levels of coverage, opportunistic STI screening of youth may be an effective intervention to reduce bacterial STI transmission in both South African youth and the general population. Screening youth and those in HIV care resulted in large reductions of population-wide bacterial STI incidence and prevalence. Screening those in HIV care was the most effective strategy for reducing population-wide STI transmission despite relatively low levels of STI prevalence in patients in HIV care, especially compared to youth (Table 3). This is due to the fact that they comprise a large fraction of the population and they have a higher level of healthcare engagement than youth. Our model demonstrated that while partner notification results in larger reductions in STI transmission, it is not necessary for a sustained reduction in population-wide chlamydia and gonorrhoea prevalence.

We estimated that ANC targeted screening resulted in a modest reduction in STI transmission in comparison with other strategies, despite high screening coverage in this subpopulation. South Africa has the lowest fertility rate in sub-Saharan Africa (Beatty 2016), and as a result, the pregnant population comprises a relatively small fraction of the total population. The modest impact of the intervention may therefore be due to the relatively small population of pregnant women when compared to the sizes of the populations targeted in the youth and HIV care screening strategies. While STI screening has been shown to be acceptable and feasible in low-income setting where women are already attending antenatal clinics for routine check-ups (Wynn et al. 2016), there are limited empirical data on its effectiveness at reducing both STI transmission and adverse birth outcomes (Mullick et al. 2005).

FSW screening was shown to be the least effective but most efficient screening strategy overall. This is partly due to the high prevalence of STIs in FSWs and the small size of the FSW population relative to the other subpopulations. The prevalence of chlamydia in FSWs is slightly higher than the general population, and as a result, FSWs do not play a large role in sustaining chlamydia transmission at a population level. In contrast, gonorrhoea prevalence in FSWs is almost 10 times that of the general population, so there is a relatively greater impact of FSW screening on gonorrhoea incidence. At a population level, there was a relatively smaller impact of opportunistic screening for gonorrhoea compared to chlamydia. This is likely due to the relatively low population prevalence of gonorrhoea in this model resulting in less statistical power to observe the impact of the intervention. A Gini coefficient of 0.40 was calculated for gonorrhoea, higher than the value of 0.37 for chlamydia, indicating greater concentration of gonorrhoea in high-risk groups, like FSW, and thus targeted screening of other subpopulations is less effective.

Previously published models of chlamydia screening have reported substantially different outcomes (Kretzschmar et al. 2009), creating uncertainty in health policy decision-making. We considered the standardised screening programme described by Kretzschmar et al. to compare the predicted impact of a 10-year youth-directed screening chlamydia programme to that estimated by the HPA, RIVM and ClaSS models (Kretzschmar et al. 2009). Our model yielded similar estimates to those from the RIVM model, but not to the ClaSS and HPA models. The previously published models discussed in this study were developed to inform STI screening guidelines in high-income settings (Kretzschmar et al. 1996; Turner et al. 2006; Low et al. 2009). The MicroCOSM model has been parametrised to the South African setting, and as a consequence, we would not necessarily expect the simulated intervention impact to be the same across models.

There were substantial differences in the model characteristics and chlamydia natural history parameters between the MicroCOSM model and the three previously published models (Electronic Supplementary Material 1, Tables S15 and S16). Most notably, our model assumed a longer duration of asymptomatic infection and included the possibility of temporary immunity after treatment which was not present in the other models. In our model, both the duration of immunity and duration of asymptomatic infection were negatively correlated with the impact of the screening intervention. There is substantial uncertainty regarding the nature and magnitude of immunity following recovery from chlamydia and gonorrhoea, and our findings must therefore be viewed with caution. However, there is evidence of partial strain-specific immunity after both chlamydia (Batteiger et al. 2010) and gonorrhoea (Moodley et al. 2002) and it has been hypothesised that early treatment of chlamydia may interrupt the development of natural immunity resulting in a higher proportion of susceptible individuals in the population (Brunham et al. 2005). Previous modelling studies demonstrated that including partial immunity for both chlamydia and gonorrhoea resulted in estimates that better fit empirical STI prevalence compared to models that do not allow for immunity (Johnson et al. 2011; Omori et al. 2019). Additionally, models that included temporary immunity predicted substantially lower impacts of therapeutic STI interventions, most likely because the direct benefit of the intervention in the short term is offset by a longer-term reduction in the prevalence of immunity (Johnson et al. 2011). All other things being equal, we might therefore expect MicroCOSM to estimate a lower impact of chlamydia screening than the other models.

However, model differences can also be explained in terms of behavioural factors. Previous studies have shown that greater heterogeneity in modelled risk of infection results in a lower impact of STI prevention interventions (Johnson et al. 2012b). The ClaSS model substantially overstated the extent of heterogeneity in the distribution of chlamydia risk in the UK (Gini coefficient = 0.84) (Althaus et al. 2011) and predicted the lowest impact of the 10-year screening programme (Kretzschmar et al. 2009; Althaus et al. 2011). The impact of the screening programme estimated by our model was similar to that of RIVM model, which was found to best describe the dynamics of chlamydia transmission, sexual partnerships and the distribution of chlamydia infection in the UK population (Althaus et al. 2011). Although our allowance for immunity and greater assumed duration of asymptomatic infection would be expected to lead to a lesser impact of screening in our model compared to the RIVM model, this effect is offset by the greater degree of heterogeneity in the distribution of chlamydia risk in the RIVM model (Gini coefficient = 0.46) compared to our model (Gini coefficient = 0.37).

A strength of this study is that we utilised a model that has been parametrised to South African sexual behaviour and STI epidemiology to investigate the impact of dual screening for chlamydia and gonorrhoea in a low-resource setting. Globally, STI screening guidelines have focused on chlamydia specifically, and few have called for gonorrhoea screening. This reflects the STI disease burden in high-income countries, in which the prevalence of gonorrhoea is negligible compared to the prevalence of chlamydia (Newman et al. 2015). Due to the overall low prevalence of gonorrhoea and the threat of increasing antimicrobial resistance, opportunistic screening for gonorrhoea is not recommended unless clinically indicated, with recent modelling studies, suggesting that increasing screening for NG in high-risk population may result in a proliferation of resistant strains that may not be offset by the initial reduction of population prevalence (Chan et al. 2012; Grad et al. 2018). In South Africa, however, the prevalence of gonorrhoea is almost half the prevalence of chlamydia (Johnson et al. 2012a), and there is thus a need for both chlamydia and gonorrhoea screening. A limitation of this study was that we did not consider the development of gonorrhoea antibiotic resistance in response to widespread gonorrhoea screening and treatment. Gonorrhoea resistance to first-line antibiotics is a global concern (Ohnishi et al. 2011; Unemo 2013), and recent modelling studies have highlighted the importance of considering the spread of gonorrhoea resistance when developing public health recommendations for gonorrhoea screening (Grad et al. 2018).

When modelling the impact of STI screening, there are concerns about the validity of assumptions surrounding sexual contact structure and the natural history of STIs (Low et al. 2009). Comparative modelling studies have demonstrated how differences in the specifications of heterogeneity in sexual risk behaviour can explain the differing results of previously published models evaluating the effectiveness of STI screening (Kretzschmar et al. 2009; Althaus et al. 2011). A limitation of our analysis is that we do not consider this uncertainty when assessing the impact of STI screening. An additional limitation is that we have run a single simulation for each of the 100 parameter combinations used to generate the model results, and measures of uncertainty may therefore be exaggerated by stochastic variation.

A further limitation of this study is that our model has been calibrated using South African STI prevalence data from specific locations that are not nationally representative, as there is currently no routine chlamydia and gonorrhoea surveillance in South Africa. Additionally, the model is not geographically stratified and therefore cannot evaluate screening strategies targeting high-risk areas. In this version of MicroCOSM, only heterosexual STI transmission is modelled.

This model assumed STI test specifications consistent with the Cepheid Dual Xpert CT/NG rapid POC test that is commonly used in high-income countries. The Xpert CT/NG test has a substantially higher cost than commercially available NAATs, and the feasibility of implementing this test in resource-poor settings has yet to be determined (Herbst de Cortina et al. 2016). There is a need for simple and inexpensive POC tests that can be delivered in a single patient that may be utilised in a South African setting.

### Conclusions

Youth-directed opportunistic screening may significantly reduce STI transmission in South African youth, despite low levels of healthcare engagement in this high-risk population. While all four screening strategies resulted in reductions in general population STI transmission, opportunistic chlamydia and gonorrhoea screening of youth and those accessing antiretroviral treatment are likely to be the most effective interventions for reducing bacterial STI transmission in the South African population, and screening of sex workers is likely to be the most efficient strategy.

## Electronic supplementary material

Below is the link to the electronic supplementary material.

Supplementary material 1 (PDF 754 kb)

Supplementary material 2 (PDF 1079 kb)
