# Supplementary material for: Modelling the impact of screening for chlamydia and gonorrhoea in youth and other high-prevalence groups in a resource-limited setting
Source: Int J Public Health. 2020 Apr 9;65(4):413–23. doi: 10.1007/s00038-020-01351-0 (PMC7274998; doi:10.1007/s00038-020-01351-0)
Supplement: Supplementary file 1 — Supplementary material 1 (PDF 754 kb) [file 38_2020_1351_MOESM1_ESM.pdf]

Electronic Supplementary Material 1
Title: The impact of screening for chlamydia and gonorrhoea in youth and other high-prevalence groups in a resource-limited setting: insights from mathematical modelling
Journal: International Journal of Public Health
Authors:
Rachel T. Esra, MPH, University of Cape Town, South Africa
racheltalesra@gmail.com
Dr Leigh F. Johnson, PhD, University of Cape Town, South Africa
leigh.johnson@uct.ac.za

1. Model of Sexual Behaviour

The model is an extension of the MicroCOSM (Microsimulation for the Control of South African Morbidity and Mortality) model, a previously developed mathematical model of heterosexual Human Immunodeficiency Virus (HIV) and sexually transmitted infection (STI) transmission in South Africa. For the purpose of the STI screening model we will simulate the transmission of HIV, *Chlamydia trachomatis* (CT) and *Neisseria gonorrhoeae* (NG). For a detailed description of the model calibration, risk-group specification, data fitting procedures and data sources on which model assumptions are based, please refer to Supplemental Digital Content 2 Appendix of this publication. Electronic Supplementary Material 2 is largely based on the original publication of the MircoCOSM model (Johnson and Geffen 2016) and has been reproduced here for convenience. Additionally, the model fitting and data validation procedures used to validate this model have been previously published (Johnson et al. 2010).

2. Mathematical modelling of sexually transmitted infection transmission and natural history

2.1 Mathematical model of chlamydia and gonorrhoea including opportunistic sexually transmitted infection screening

The mathematical model of the natural history of gonorrhea and chlamydia in the original model is described in detail in Supplemental Digital Content 2 and has been reproduced here to include opportunistic STI screening as illustrated in Figure S2 and summarised in Table S1. The models used for CT and NG are identical in structure but different parameters are assigned to each. In short, susceptible individuals become infected at rate λ, and newly infected individuals may develop symptoms or remain asymptomatic. Both states may result in temporary immunity after spontaneous resolution of infection or after receiving treatment. Symptomatic individuals seek treatment at rate υ, which is successful at curing the infection with the probability ψ. The natural history parameters assigned to chlamydia and gonorrhea are summarised in Electronic Supplementary Material 2 in tables S8 and S9.

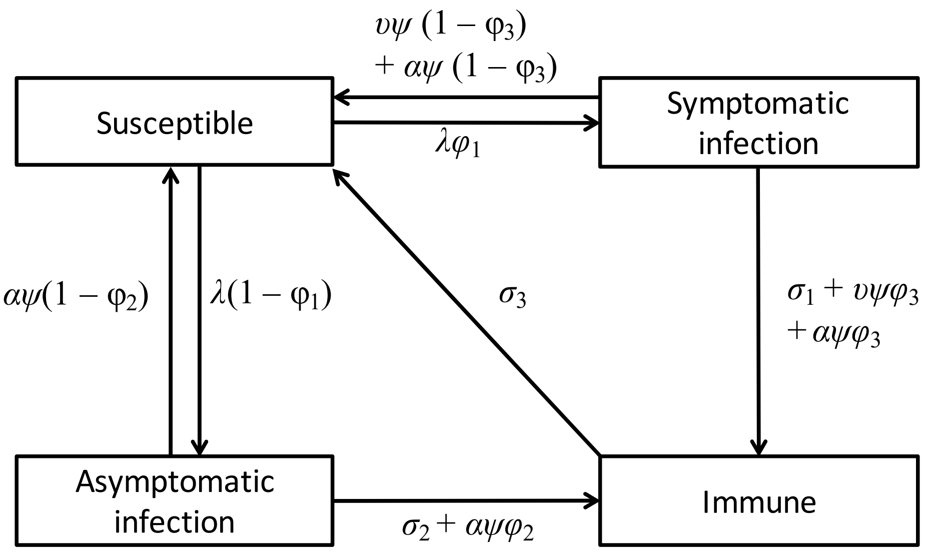

Figure S1: Multi-State model of the natural history of chlamydia and gonorrhoea
Updated from the MicroCOSM model (Johnson and Geffen 2016) to include opportunistic STI screening

Both symptomatic and asymptomatic individuals will be screened at rate α, which is a product of three independent probabilities:
(1) The probability of attending a health service where screening is available
(2) The probability of being offered a screening test
(3) The probability of accepting a screening test

These parameters have been calculated separately for each population based on estimates from the literature. For both CT and NG, probability distributions were specified to represent uncertainty around key natural history and transmission parameters as described in Electronic Supplementary Material 2. In the previous application of the model, immunity following successful treatment of asymptomatic infection was not considered, since the model considered only treatment of symptomatic infection. Due to lack of data on immunity following successful treatment, we assume here that the extent of immunity following the successful treatment of asymptomatic infection is the same as that for symptomatic infection (i.e. $\phi_2 = \phi_3$).

Table S1: Key natural history parameters

Symbol	Definition
φ_1	Proportion of people who become symptomatic
φ_2	Proportion of people temporarily immune after experiencing resolution of an asymptomatic infection after treatment
φ_3	Proportion of people temporarily immune after experiencing resolution of a symptomatic infection after treatment
σ_1	Rate at which symptomatic people recover in the absence of treatment
σ_2	Rate at which asymptomatic people recover in the absence of treatment
σ_3	Rate at which immunity wanes

2.2 Mathematical model of sexually transmitted infection screening

We have introduced the components of a hypothetical 10-year screening intervention while keeping parameters describing transmission and duration of infection the same as in the original model (Johnson and Geffen 2016).

Four targeted screening programs are simulated:

1. **Youth screening:** Males and females aged 15-24 are eligible for annual screening. This screening strategy is in line with global Chlamydia screening policies (Hughes et al. 2014; Nelson et al. 2014)
2. **Antenatal care (ANC) screening:** The Centres for Disease Control recommend antenatal STI screening for CT and NG for all women at the onset of prenatal care, and again in the third trimester for women who are younger than 25 years or at increased risk (Centers for Disease Control and Prevention 2015). Our model does not simulate the duration of pregnancy but rather occurrence of a new birth. Because of this, antenatal screening is simulated as occurring at the time of birth.
3. **Female Sex Worker (FSW) screening**: All FSWs are eligible for annual screening.
4. **HIV Care Screening:** HIV positive individuals, above the age of 14 and either on ART or in the symptomatic stages of disease (WHO stages III and IV) are eligible for annual STI screening. Recent changes in South African guidelines recommend that all HIV positive individual should be initiated onto ART treatment regardless of CD4 counts (South African Department of Health 2016), therefore theoretically all individuals with a known HIV positive status should be accessing ART.

For the populations in which the coverage of STI screening is known (ANC), a single probability of screening is applied (Table S1). For populations in which the coverage of screening in not known (youth, FSW and ART patients), an annual rate of screening has been calculated as the product of the the probability of annual health care utilisation, STI screening acceptability and screening coverage (Table S1). A random number between 0 and 1 is assigned to each eligible individual at the start of each weekly time step. All individuals assigned random probabilities below the probability of screening are screened for STIs and treated with a rate of success described by the model above (Figure 1).

Table S2: Model parameters

Parameter	Females	Males	Source
**Probability of screening in populations with known screening rates**			
ANC	0.71	N/A	Literature (Dinh et al. 2013)
**Assumed rate of screening in populations for which screening rates are unknown**			
**Screening coverage**	0.80	0.80	
**Annual Health Care Utilisation rate**			
Youth (15-24)	0.48	0.32	GHS (Statistics South Africa. 2017)
FSWs	0.50	N/A	Literature (Department of Health Republic of South Africa; Ramesh et al. 2010; Richter et al. 2014; Wong et al. 2015; Kohler et al. 2016)
HIV care*	0.90	0.90	Literature (Johnson et al. 2017; Takuva et al. 2017)**
**Screening acceptability**			
Youth (15-24)	0.60	0.60	Literature (Ostergaard et al. 1998; Cohen et al. 1999; Shafer et al. 2002; Monroe et al. 2003; Kent et al. 2004; Marrazzo et al. 2007; Goldenkranz Salomon et al. 2012a; Miller et al. 2015; Goyal et al. 2016)
FSWs	0.80	N/A	Literature (Ramjee et al. 1998; Morton et al. 1999; Wong et al. 2015)
HIV care	1.00	1.00	Literature (Takuva et al. 2017)
**Annual screening rate**			
Youth (15-24)	0.23	0.15	

FSWs	0.32	N/A	The total screening probability was calculated as the product of heath care utilisation, screening acceptability and screening coverage.
HIV care	0.72	0.72	
**Partner Notification*** **			
Proportion of partners screened	0.50	0.50	Literature (van de Laar et al. 1997; Golden et al. 2003, 2005; Kissinger et al. 2005; Low et al. 2007; Cameron et al. 2009; Herzog et al. 2011; Estcourt et al. 2015)

ANC = antenatal care, FSW = female sex worker, GHS = General Household Survey
* HIV positive individuals, above the age of 14 and either on ART or in the symptomatic stages of HIV disease (WHO stage III or IV) are eligible for annual STI screening.
** Based on reports that that the majority of South Africans on ART receive laboratory monitoring at least annually (Takuva et al. 2017)
*** Only applicable in scenarios where partner notification is implemented

In all scenarios individuals are screened for both chlamydia and gonorrhoea simultaneously under the assumption that point of care (POC) testing would be performed with a POC test with dual testing capabilities, such the Cepheid GeneXpert CT/NG (Xpert) assay (Gaydos et al. 2013). The GeneXpert CT/NG is considerably more accurate than other commercially available POC tests (Herbst de Cortina et al. 2016) and demonstrates higher sensitivities using endocervical/urethral samples in comparison to urine samples (Gaydos et al. 2013). Despite the slightly reduced sensitivity, urine based STI testing yields higher rates of acceptability compared to more invasive sample collection (Cohen et al. 1999; Andersen et al. 2016; Low et al. 2016). Based on this, the sensitivity of STI testing in youth, FSW and HIV care targeted screening scenarios is assumed to be the same as the GeneXpert sensitivity for urine samples (97.4% in females and 97.5% in males for CT, 95.6% in females and 98.0% in males for NG) (Gaydos et al. 2013). STI testing using vaginal swab collection has been found to be acceptable when implemented as part of routine antenatal check-ups (Chen et al. 2006; Cabeza et al. 2015; Badman et al. 2016) and the sensitivity of the screening test in the antenatal strategy is therefore assumed to be the same as the GeneXpert sensitivity for vaginal samples (97.4% for CT and 100% for NG)(Gaydos et al. 2013).

2.2.1 Partner notification

Partner notification (PN) has been identified as an integral component of prevention of curable STIs, specifically STIs such as CT which is predominately asymptomatic (Althaus et al. 2014). PN is currently recommended as part of global STI screening programs and involves identifying partners of patients that test positive for curable STIs (index cases) so that they may be screened or presumptively treated (Althaus et al. 2014). Index cases in this model can have a maximum of two current partners. If screening produces a positive result, both primary and secondary (when applicable) partners are screened with a probability of 50% based on estimates from literature (Table S5). These estimates are based on studies implementing standard patient referral whereby index cases are given the responsibility of notifying partners (Table S5). Partners are treated with the same rate of success as index cases, although we acknowledge that the assumption of 50% of secondary partners being notified is optimistic. In this model only current sexual partners are considered eligible for partner notification. It is currently unknown whether index cases are more likely to notify current as opposed to former partners of their STI status, but mathematical modelling has shown that notification of only current partners is sufficient to achieve population level reduction in STI prevalence (Althaus et al. 2014). Women engaging in sex work are assumed not to form short-term or long-term relationships during the periods in which they are active as sex workers. Due to this, partner notification cannot be simulated in the FSW population.

2.2.2 Partner notification sensitivity analysis

Previously published studies of CT screening have assumed that primary and secondary partners would be notified and screened with equal probability (Kretzschmar et al. 2009). In order to investigate this further we ran a sensitivity analysis comparing youth targeted screening utilizing three different partner notification scenarios.

Run 1: Primary and secondary partners are screened with equal probability (50%). (This is the default scenario.)

Run 2: Only primary partners are screened, all with the same probability (50%).

Run 3: Only primary partners are screened. Primary partners that are marital partners are screened with a higher probability (100%) compared to non-marital partners (50%).

A comparison of these scenarios revealed that the highest number of partners screened resulted from screening primary and secondary partners with equal probabilities (Figure S3).

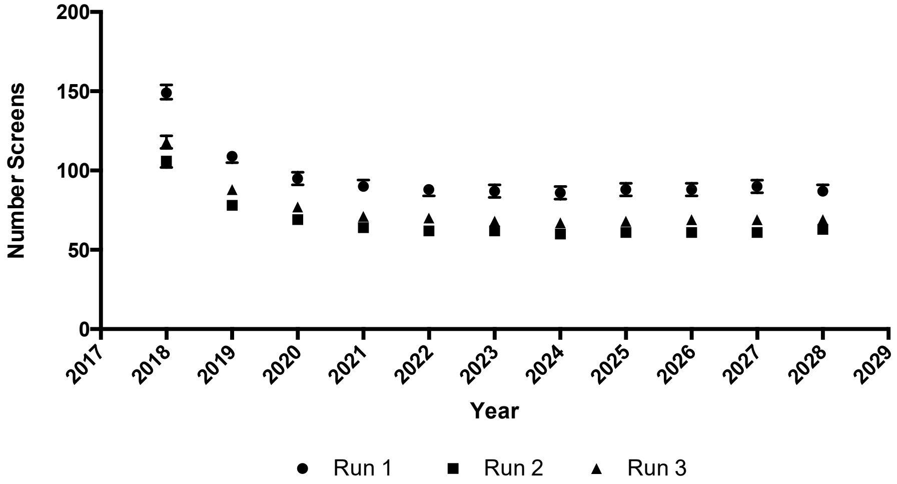

Figure S2: Comparison of number of partners screened under 3 partner notification scenarios over 10 years of youth targeted screening. Shapes represent mean number of partners screened from the range of model outputs generated when the 100 best-fitting parameter combinations are entered into each model (error bars represent 95% CIs). The population grew dynamically over the 10-year screening period with an average population size close to 36 000 mid-way through the screening period in 2023.

Table S3: % increase in STI estimates for general population (aged 15 to 49) with differing partner notification scenarios, in the context of youth-targeted screening

		Run 1 vs. Run 2	Run 1 vs. Run 3	Run 3 vs. Run 2
**% increase in cumulative incidence (2018 -2028)**				
**Chlamydia**		-1.05 (0.26-1.83)	-0.20 (-0.50-0.90)	0.88 (0.23-1.53)
**Gonorrhea**		0.15 (-1.58-1.28)	-1.38 (-0.58-3.35)	-1.00 (-2.64-0.64)
**Chlamydia**	**Females**	-1.58 (-0.26-3.42)	0.02 (-1.53-1.49)	1.91 (0.05-3.76)
	**Males**	-1.46 (-0.33-3.26)	-0.64 (-1.01-2.30)	1.21 (-0.66-3.09)
**Gonorrhea**	**Females**	-3.96 (-0.47-8.39)	-2.51 (-1.75-6.78)	3.23 (-0.88-7.35)
	**Males**	-7.34 (-1.56-13.13)	-7.03 (1.75-12.32)	2.41 (-2.4-7.21)

3. Estimation of screening parameters

Where possible, the health seeking behaviour of different South African populations has been calculated from South African national survey data and epidemiological studies on the impact and behaviours associated with STI screening (Table S1). In the cases where there is no current data on these behaviours in the South African population, data sources from other countries, where screening for CT and NG has been implemented, have been considered. Studies selected for parameterization comprise of observational studies, where investigators have reported samples of youths, pregnant women, female sex workers and those in HIV care, in South Africa and other countries.

The probability of antenatal screening for bacterial STIs is assumed to be 0.71, equal to the current coverage of antenatal syphilis screening in South African women (Dinh et al. 2013). This probability is applied to all pregnant women at the occurrence of childbirth.

For all other sub-populations, a screening coverage of 80% is assumed from 2018 until 2028 for all screening strategies, i.e. eligible individuals who attend health facilities are assumed to be offered screening with 80% probability. Youth are assumed to access healthcare at an annual rate of 0.48 for females and 0.32 for males (Statistics South Africa. 2017). Based on estimates from literature on the acceptability of STI screening using urine based testing, youth acceptability of STI screening is assumed to be 60% (Table S6). There is no empirical data on the STI screening acceptability in South Africans linked to HIV care. South African HIV treatment guidelines recommend viral load testing 6 and 12 months after initiating ART treatment and annually thereafter (Department of Public Health Republic of South Africa 2015). As this population is undergoing regular viral load testing, we can assume that the majority of those on ART attend a clinic at which STI screening may be offered at least annually. Even if patients are in WHO stages III/IV but not on ART, it is likely that they would attend HIV services regularly to receive treatment for opportunistic infections, and STI screening could also be offered at these services. We assume that 90% of those on ART or in WHO stages III and IV access healthcare annually and would be amenable to opportunistic STI testing. Based on literature regarding FSWs within South Africa and internationally, FSWs are assumed to access healthcare with an annual rate of 0.50 (Table S7) and FSWs acceptability of STI screening is assumed to be 0.90 (Table S8).

Table S4: Literature review of Partner Notification rates

Study	Year	Sample	Location	STI	Partner notification type	Proportion of partners screened
**Estcourt et al.** (Estcourt et al. 2015)	2015	Females aged 16 +, in GP, GUM and pharmacies	East London, United Kingdom	CT & NG	Standard referral	45% (N=102)
**Herzog et al.** (Herzog et al. 2011)	2011	Men and women attending GUM clinics	UK	CT	Not recorded	47% (N =4616)
**Cameron et al.** (Cameron et al. 2009)	2009	Women attending FPCs or GUM clinics	Edinburgh	CT	Standard referral	41% (N=134)
**Golden et al.** (Golden et al. 2005)	2005	Heterosexual men and women	Washington, USA	CT & NG	Standard referral	78% (N= 1375)
**Kissinger et al.** (Kissinger et al. 2005)	2005	Men attending an STD clinic	New Orleans	NG & CT	Standard referral	71% (N=285)
**Low et al.** (Low et al. 2006)	2005	Men and women visiting primary care facilities and GUM clinics	Bristol and Birmingham, UK	CT	Standard referral	52.9% (N= 68)
**van de Laar et al.** (van de Laar et al. 1997)	1997	Men and women attending an STI clinic	Amsterdam, Netherlands	CT & NG	Self-referral using referral cards	40% (N = 580)

CT = Chlamydia trachomatis, FPC = family planning clinic, GUM = genitourinary medicine, NG = Neisseria gonorrhoeae, UK = United Kingdom, USA = United States of America

Table S5: Literature review of Youth STI screening acceptability

Study	Year	Sample	Location	STI	Test	Screening acceptability
**Goyal et al.** (Goyal et al. 2016)	2016	14-21-year-old asymptomatic males and females presenting to ED	Washington, DC	CT & NG	Urine based PCR	59% (N= 553)
**Goldenkranz et al.** (Goldenkranz Salomon et al. 2012b)	2012	10-14-year-old females visiting Title FP X clinics	Region X (Alaska, Idaho, Oregon and Washington)	CT	Not reported	34.5% (N= 2697)
		15-19-year-old females visiting Title FP X clinics				40.7% (N = 56 400)
		20-24-year-old females visiting Title FP X clinics				40.7 % (N= 81 180)
**Marrazzo et al.** (Marrazzo et al. 2007)	2007	Asymptomatic males in adolescent primary care	Baltimore, Denver, San Francisco and Seattle	CT	Urine based PCR or SDA	61% (N= 93)
		Asymptomatic males in adolescent school based clinics				49% (N= 412)
		Asymptomatic males in Juvenile detention centers				68% (N=879)
**Kent et al.** (Kent et al. 2004)	2004	Male and female students at 4 high schools	San Francisco	CT & NG	Urine based LCR	14% (N= 4497)
**Monroe et al.** (Monroe et al. 2003)	2003	14 -20-year-old asymptomatic males and females presenting to ED	Texas	CT & NG	Urine based LCR	71% (N= 1231)
**Shafer et al.** (Shafer et al. 2002)	2002	14-18-year-old females in schools	Northern Caroline	CT	SDA	47% (N= 1071)
**Cohen et al.** (Cohen et al. 1999)	1999	9-12th grade high school male and females	Louisiana	CT	Urine based LCR	52 – 65% (N= 6000+)

CT = Chlamydia trachomatis, ED = emergency department, FP = family planning, LCR = ligase chain reaction, NG = Neisseria gonorrhoeae, PCR = polymerase chain reaction, SDA = strand displacement amplification

Table S6: Literature review of FSW STI Health seeking

Study	Year	Sample	Location	Health Seeking in the last year (at least once)	
				General	STI Screening
**Kohler et al.** (Kohler et al. 2016)	2016	FSWs ≥14	Peru	Not reported	52.3 -57.2 %
**SAHMS Final Report** (Department of Health Republic of South Africa)	2015	FSWs ≥ 16	Johannesburg	53.8%	Not reported
			Cape Town	36.2%	
			Durban	58.8%	
**Wong et al.** (Wong et al. 2015)	2015	FSWs	Hong Kong	Not reported	45% (N = 340)
**Richter et al.** (Richter et al. 2014)	2014	FSWs	South Africa	60% interacted with health service in the last month	Not reported
**Ramesh et al.** (Ramesh et al. 2010)	2009	FSWs	Karnataka, India	68.4% had visited project sexual health clinic (baseline)	Not reported

Table S7: Literature review of FSW STI Screening acceptability

Study	Year	Sample	Location	STI	Test	Screening acceptability
**Wong et al.** (Wong et al. 2015)	2015	FSWs	Hong Kong	HIV, Syphilis, CT & NG	Self -collected Urine/ physician collect ectocervical swab based NAAT	41.6% (N=818)
**Morton et al.** (Morton et al. 1999)	1999	Male, female and transsexual sex-workers	Melbourne	CT, NG & TV	Self-collected tampon (females) or urine (males/ transsexuals) based PCR	78% (N=81)
**Ramjee at al.** (Ramjee et al. 1998)	1997	FSWs	Kwa-Zulu Natal	HIV, Syphilis, CT, NG, Trichomonas and BV	Clinician obtained cervical samples based direct immunofluorescence	100% (N=145)

BV = Bacterial Vaginosis , CT = Chlamydia trachomatis, HIV = Human Immunodeficiency Virus, NAAT = Nucleic acid amplification test ,NG = Neisseria gonorrhoeae, PCR = polymerase chain reaction, TV = Trichomonas vaginalis

4. Correlates of Model Outputs

Tables S8 and S9 show the relationship between natural history and transmission parameters of chlamydia and gonorrhea and the reduction in STI transmission using different screening strategies. This relationship is described by the Pearson correlation coefficient, and was calculated using the 100 parameter combinations that yielded the best fit to the STI prevalence data. When considering which parameters had the largest effect on the impact of the screening intervention, a number of points emerge:

- The CT transmission probabilities were positively correlated with the reduction in chlamydia incidence and prevalence.
- The average duration of CT immunity was negatively associated with a reduction in chlamydia incidence and prevalence. This is because the direct benefit of the intervention in the short term is offset by a longer-term reduction in the prevalence of immunity (Johnson et al. 2011), and longer durations of immunity are associated with greater offsets.
- The duration of asymptomatic CT infection was also negatively associated with impact of the screening intervention. Since the average duration of asymptomatic infection is strongly positively associated with average duration of immunity (correlation = 0.61), it is likely that the association is confounded by the immunity parameter and it is the latter that is driving the observed association.
- For the most part, the same relationship between the average duration of immunity was not observed for NG. The strongest relationships between immunity parameters for CT and the impact of the screening intervention were observed in the screening strategies that showed the largest impacts (targeted at youth and HIV Care in comparison to ANC and FSWs). Only in the case of FSW screening, targeting a subpopulation with the highest NG prevalence, was the impact of screening negatively correlated with the average duration of NG immunity. We suspect that NG immunity is generally associated with the impact of the screening intervention but the low population prevalence of NG in this model results in less statistical power to observe this relationship.
- The proportion of female NG cases that become symptomatic was positively correlated with the impact of the screening intervention. In scenarios in which there is a higher proportion of symptomatic STI cases, there would be a higher rate of health seeking behaviour resulting in lower baseline prevalence on average. Since NG is closer to the threshold of persistence relative to CT (i.e. closer to $R_0 = 1$), this may result in NG becoming extinct in scenarios where a larger proportion of cases become symptomatic, resulting in the same intervention having a relatively higher impact on NG prevalence compared to CT.

Table S8: Correlates of reductions in chlamydia incidence and prevalence

Scenario	% Change relative to baseline	Transmission Probability		% of cases that become symptomatic		Average duration (in years)				
		M-to-F	F-to-M	Male	Female	Symptomatic infection	Asymptomatic infection	Immunity post treatment	% immune after treatment	% Correctly treated
**Youth Screening**										
Cumulative incidence (2018 -2028)		0.16	0.29**	0.02	-0.02	-0.15	-0.33**	-0.28**	-0.02	-0.01
Prevalence (2028)										
	Females (15 – 49)	0.17	0.16	-0.06	-0.02	-0.09	-0.46**	-0.13	-0.24*	0.01
	Males (15 – 49)	0.13	0.22*	-0.02	-0.10	-0.15	-0.26**	-0.08	-0.01	-0.03
	Females (15 – 24)	0.07	-0.01	-0.15	-0.11	0.01	-0.36**	-0.22*	-0.12	0.05
	Males (15 – 24)	0.12	0.15	-0.14	-0.10	0.04	-0.22*	-0.14	0.10	0.07
**Youth Screening with Partner Notification**										
Cumulative incidence (2018 -2028)		0.24*	0.24*	0.08	0.14	0.05	-0.38**	-0.53**	-0.12	0.01
Prevalence (2028)										
	Females (15 – 49)	0.26**	0.11	-0.14	-0.03	0.07	-0.57**	-0.33**	-0.30**	-0.03
	Males (15 – 49)	0.13	0.22*	-0.02	-0.10	-0.15	-0.26**	-0.08	-0.01	-0.03
	Females (15 – 24)	0.14	0.02	-0.14	-0.09	0.08	-0.46**	-0.33**	-0.21*	-0.04
	Males (15 – 24)	0.03	0.19	0.02	-0.08	0.15	-0.34**	-0.19	-0.25*	-0.01
**Female Sex Worker**										
Cumulative incidence (2018 -2028)		0.22*	0.03	0.01	0.09	-0.22*	-0.11	-0.02	0.04	0.02
Prevalence (2028)										
	Females (15 – 49)	0.20*	0.00	-0.07	0.18	-0.11	-0.12	0.00	-0.05	-0.07
	Males (15 – 49)	0.14	0.05	-0.04	0.09	-0.18	-0.03	-0.09	0.09	-0.02
	FSW	0.05	0.04	0.00	0.07	-0.09	0.03	0.01	0.08	-0.02

*p <0,05 **p <0,01

Estimated trends for South Africa from 2018-2028

Table S8 continued

**Antenatal Screening**									
Cumulative incidence (2018 -2028)	0.12	0.14	0.02	-0.05	-0.21*	-0.17	-0.19*	0.00	0.00
Prevalence (2028)									
Females (15 – 49)	0.11	0.05	-0.07	0.04	-0.15	-0.14	0.00	-0.08	-0.04
Males (15 – 49)	0.00	0.14	0.09	-0.04	-0.18	0.05	-0.02	0.08	0.00
ANC	0.13	0.03	-0.04	0.05	-0.16	-0.11	-0.07	-0.04	0.00
**Antenatal Screening with Partner Notification**									
Cumulative incidence (2018 -2028)	0.15	0.24*	0.12	-0.03	-0.17	-0.20*	-0.10	-0.04	-0.09
Prevalence (2028)									
Females (15 – 49)	0.12	0.26**	0.07	0.06	-0.12	-0.21*	-0.12	-0.06	-0.08
Males (15 – 49)	0.09	0.27**	0.07	-0.01	-0.15	-0.07	-0.18	0.06	-0.10
ANC	0.10	0.25**	0.05	0.05	-0.10	-0.19*	-0.15	-0.06	-0.06
**HIV Care Screening**									
Cumulative incidence (2018 -2028)	0.27**	0.33**	0.05	0.08	-0.17	-0.13	-0.33**	0.18	-0.10
Prevalence (2028)									
Females (15 – 49)	0.17	0.06	-0.12	0.09	-0.16	-0.11	-0.18	0.03	-0.12
Males (15 – 49)	0.09	0.08	0.03	0.02	-0.24*	0.03	-0.17	0.09	-0.15
Females HIV care	0.21*	0.17	-0.09	0.03	-0.06	-0.53**	-0.26**	-0.24*	-0.25*
Males HIV care	0.19	0.10	-0.08	-0.05	-0.10	-0.41**	-0.31**	-0.14	-0.11
**HIV Care Screening with Partner Notification**									
Cumulative incidence (2018 -2028)	0.21*	0.41**	0.19	-0.04	-0.12	-0.18	-0.26**	0.14	-0.02
Prevalence (2028)									
Females (15 – 49)	0.28**	0.23*	0.03	0.04	-0.06	-0.13	-0.05	0.04	-0.06
Males (15 – 49)	0.16	0.31**	0.09	-0.03	-0.18	0.00	-0.08	0.17	-0.07
Females HIV care	0.22*	0.21*	0.01	0.13	0.06	-0.38**	-0.27**	-0.13	-0.23*
Males HIV care	0.14	0.11	-0.10	0.00	-0.01	-0.32**	-0.29**	-0.04	-0.05

*p <0,05 **p <0,01

Estimated trends for South Africa from 2018-2028

Table S9: Correlates of Gonorrhea natural history parameters and model outputs

		Transmission Probability		% of cases that become symptomatic		Average duration (in years)			Immunity	
Scenario	% Change relative to baseline	M-to-F	F-to-M	Male	Female	Symptomatic infection	Asymptomatic infection	Immunity post treatment	% immune after treatment	% Correctly treated
**Youth Screening**										
Cumulative incidence (2018 -2028)		-0.01	-0.02	0.09	0.07	-0.03	-0.22*	-0.17	-0.03	-0.08
Prevalence (2028)										
	Females (15 – 49)	0.03	-0.12	-0.11	0.16	-0.01	0.03	-0.06	0.04	0.08
	Males (15 – 49)	0.03	-0.10	-0.09	0.15	-0.03	-0.05	-0.11	0.02	0.01
	Females (15 – 14)	0.02	-0.07	-0.16	0.05	-0.12	-0.09	-0.13	0.06	0.02
	Males (15 – 24)	0.01	0.13	0.06	0.09	-0.05	-0.11	-0.14	0.06	0.03
**Youth Screening with Partner Notification**										
Cumulative incidence (2018 -2028)		-0.08	0.11	-0.06	0.09	-0.03	0.00	-0.08	0.05	-0.14
Prevalence (2028)										
	Females (15 – 49)	-0.13	0.06	-0.09	0.20*	0.02	0.12	-0.07	0.08	0.01
	Males (15 – 49)	-0.08	0.03	-0.15	-0.09	-0.04	-0.06	0.11	-0.02	-0.18
	Females (15 – 14)	-0.12	0.12	0.00	0.07	-0.05	-0.04	-0.16	0.17	0.01
	Males (15 – 24)	-0.07	0.07	0.00	0.07	-0.11	-0.03	-0.24*	0.14	0.01
**Female Sex Worker**										
Cumulative incidence (2018 -2028)		0.01	-0.07	0.07	0.16	-0.11	-0.05	-0.30**	0.14	-0.07
Prevalence (2028)										
	Females (15 – 49)	-0.01	-0.07	-0.05	0.25**	0.15	0.04	-0.14	0.13	0.08
	Males (15 – 49)	-0.06	-0.05	-0.05	0.20*	0.08	0.07	-0.15	0.13	-0.06
	FSW	0.09	-0.09	0.12	0.15	0.10	0.07	-0.05	-0.03	-0.02

* p <0.05 **p <0.01

Estimated trends for South Africa from 2018-2028

Table S9 continued

**Antenatal Screening**									
Cumulative incidence (2018 -2028)	-0.05	-0.01	-0.04	0.24*	0.12	-0.10	-0.06	-0.02	-0.06
Prevalence (2028)									
Females (15 – 49)	-0.01	-0.04	-0.11	0.27**	0.04	0.05	-0.17	0.10	0.03
Males (15 – 49)	-0.01	-0.13	-0.11	0.25**	0.06	0.08	-0.18	0.12	0.00
ANC	0.01	-0.04	-0.11	0.24*	0.03	0.05	-0.16	0.11	0.03
**Antenatal Screening with Partner Notification**									
Cumulative incidence (2018 -2028)	-0.03	-0.01	-0.08	0.18	0.01	0.00	-0.05	-0.06	0.04
Prevalence (2028)									
Females (15 – 49)	-0.09	-0.02	0.02	0.14	0.08	0.14	-0.05	0.02	0.06
Males (15 – 49)	-0.07	-0.03	0.08	0.21*	0.13	0.17	-0.08	0.07	0.00
ANC	-0.08	-0.04	0.00	0.12	0.08	0.16	0.00	0.00	0.08
**HIV Care Screening**									
Cumulative incidence (2018 -2028)	0.04	0.04	-0.03	0.05	-0.12	-0.11	-0.21*	0.09	0.00
Prevalence (2028)									
Females (15 – 49)	-0.06	0.09	0.08	0.20*	-0.06	0.03	-0.11	0.05	0.03
Males (15 – 49)	-0.09	0.07	0.04	0.20*	-0.07	0.04	-0.12	0.08	-0.05
Females HIV care	0.08	0.05	0.09	0.18	0.04	-0.10	-0.23*	0.06	-0.02
Males HIV care	-0.10	0.09	0.13	0.10	-0.16	0.16	-0.16	0.14	-0.05
**HIV Care Screening with Partner Notification**									
Cumulative incidence (2018 -2028)	0.05	0.11	0.00	0.34**	-0.02	-0.04	-0.14	-0.04	0.05
Prevalence (2028)									
Females (15 – 49)	-0.14	0.03	-0.06	0.25**	0.01	0.26*	-0.10	0.15	0.03
Males (15 – 49)	-0.19*	0.01	-0.06	0.25**	-0.07	0.24	-0.20*	0.18	-0.02
Females HIV care	0.09	-0.06	-0.03	0.03	0.12	-0.01	-0.16	0.17	-0.01
Males HIV care	-0.10	0.04	0.00	0.03	-0.25*	0.21	-0.14	0.12	-0.05

* p <0.05 **p <0,01

Estimated trends for South Africa from 2018-2028

5. Additional model outputs

Table S10: Cumulative incidence over the 10-year targeted screening program

	Cumulative incidence over 10 years (95% CI)		% Reduction in Cumulative incidence relative to baseline	
Scenario	Chlamydia	Gonorrhea	Chlamydia	Gonorrhea
**Baseline***	11292 (10866-11718)	19168 (18004-20333)		
**Youth Screening**				
Without PN	10670 (10239-11101)	18614 (17454-19774)	5.72 (5.05-6.39)	3.00 (1.67-4.32)
With PN	10496 (10061-10931)	18489 (17346-19632)	7.37 (6.62-8.11)	3.4 (2.07-4.72)
**FSW Screening**				
Without PN	11216 (10790-11642)	18737 (17567-19908)	0.68 (0.18-1.19)	2.40 (1.33-3.47)
**Antenatal Care screening**				
Without PN	11092 (10661-11523)	18869 (17731-20007)	1.87 (1.34-2.40)	1.20 (-0.14-2.55)
With PN	10991 (10565-11418)	18905 (17765-20045)	2.77 (2.2-3.34)	1.05 (-0.25-2.35)
**HIV Care screening**				
Without PN	10412 (9996-10827)	18214 (17061-19368)	7.94 (7.33-8.56)	5.20 (4.02-6.38)
With PN	10184 (9768-10601)	18130 (16996-19264)	10.05 (9.33-10.78)	5.45 (4.06-6.83)

The population grew dynamically over the 10-year screening period with an average population size close to 36000 mid-way through the screening period.
*Baseline incidence estimates were calculated over the same 10-year period as the hypothetical screening intervention
The means and 95% CIs are calculated from the range of model outputs generated when the 100 best-fitting parameter combinations are entered into the model. Estimated trends for South Africa from 2018-2028.

Table S11: Estimated impact of targeted screening programs on population level prevalence (aged 15 to 49) after the implementation of a 10-year targeted screening program

	% Reduction in prevalence 10th Year (95% CI)			
	Chlamydia		Gonorrhea	
Scenario	Females	Males	Females	Males
Youth vs. Baseline	15.09 (13.82-16.35)	13.75 (12.25-15.25)	3.34 (-0.50-7.18)	1.29 (-3.56-6.15)
Youth PN vs. Baseline	17.38 (16.00-18.75)	17.29 (15.91-18.67)	2.30 (-1.52-6.13)	1.12 (-3.67-5.91)
Youth vs. Youth PN	2.52 (1.17-3.87)	3.75 (2.20-5.30)	-2.77 (-6.54-1.00)	-2.34 (-6.71-2.03)
FSW vs. Baseline	0.94 (-0.23-2.10)	1.34 (-0.02-2.69)	1.41 (-2.35-5.18)	0.34 (-4.24-4.91)
ANC vs. Baseline	6.94 (5.72-8.15)	4.64 (3.23-6.04)	0.27 (-3.98-4.52)	-2.61 (-7.29-2.07)
ANC PN vs. Baseline	8.70 (7.32-10.07)	6.66 (5.18-8.14)	0.39 (-2.93-3.71)	-0.75 (-5.25-3.74)
ANC vs. ANC PN	1.66 (0.14-3.18)	1.89 (0.45-3.33)	-2.96 (-7.28-1.36)	-0.84 (-5.27-3.60)
HIV Care vs. Baseline	14.54 (13.44-15.63)	12.85 (11.52-14.17)	4.74 (0.94-8.54)	3.67 (-1.37-8.72)
HIV Care PN vs. Baseline	17.12 (15.81-18.44)	15.45 (13.93-16.96)	8.29 (4.76-11.83)	5.86 (1.40-10.32)
HIV Care vs. HIV Care PN	2.87 (1.43-4.30)	2.79 (1.21-4.36)	0.97 (-3.76-5.70)	-1.69 (-7.33-3.94)

The means and 95% CIs are calculated from the range of model outputs generated when the 100 best-fitting parameter combinations are entered into each model. The hypothetical implementation of STI screening programs is simulated as having been initiated in 2018.
PN = partner notification, FSW = female sex worker, ANC = antenatal care, PLWHA = people living with HIV/Aids, ART = antiretroviral treatment. Estimated trends for South Africa from 2018-2028.

Table S12: Estimated impact of targeted screening programs in key populations after the implementation of different 10-year targeted screening programs

Scenario	Chlamydia		Gonorrhoea	
	Females	Males	Females	Males
**Youth targeted screening: % Reduction in Youth (aged 15 to 24) prevalence**				
Youth vs. Baseline	25.70 (24.1 - 27.3)	23.06 (20.75 - 25.37)	7.14 (2.59 - 11.68)	3.06 (-2.85 - 8.97)
Youth PN vs. Baseline	30.66 (28.85 - 32.46)	31.4 (29.34 - 33.45)	7.99 (3.3 - 12.68)	2.23 (-4.56 - 9.02)
**FSW targeted screening: % Reduction in FSW prevalence**				
FSW vs. Baseline	5.93 (1.24 - 10.61)		4.88 (1.54 - 8.23)	
**ANC targeted screening: % Reduction in pregnant women prevalence**				
ANC vs. Baseline	7.16 (5.95 - 8.37)		0.14 (-4.20 - 4.49)	
ANC PN vs. Baseline	9.00 (7.61 - 10.4)		0.65 (-2.77 - 4.07)	
**HIV Care targeted screening: % Reduction in STI prevalence in patients in HIV care**				
HIV Care vs. Baseline	52.58 (50.72 - 54.44)	51.97 (49.66 - 54.28)	22.5 (18.2 - 26.80)	18.3 (11.54 - 25.05)
HIV Care PN vs. Baseline	56.27 (54.44 - 58.09)	54.72 (52.68 - 56.75)	27.26 (23.37 - 31.14)	20.04 (12.46 - 27.62)

The means and 95% CIs are calculated from the range of model outputs generated when the 100 best-fitting parameter combinations are entered into each model. The hypothetical implementation of STI screening programs is simulated as having been initiated in 2018, and the reduction in incidence and prevalence is calculated relative to the level that would have been expected in the absence of any screening program. Estimated trends for South Africa from 2018-2028.
PN = partner notification, FSW = female sex worker, ANC = antenatal care

Table S13: Estimated impact of youth (aged 15 to 24) targeted screening in different populations after a 10-year targeted screening program

Scenario	**% Reduction STI prevalence in youth (aged 15-24 years) after 10 years of screening**			
	Chlamydia		Gonorrhea	
	Females	Males	Females	Males
Youth	-25,7 (-24,1- -27,3)	-23,06 (-20,75- -25,37)	-7,14 (-2,59- -11,68)	-3,06 (2,85- -8,97)
Youth PN	-30,66 (-28,85- -32,46)	-31,4 (-29,34- -33,45)	-7,99 (-3,3- -12,68)	-2,23 (4,56- -9,02)
HIV Care	-2,78 (-1,04- -4,51)	-2,42 (-0,2- -4,64)	2,93 (7,82- -1,97)	5,26 (12,55- -2,03)
HIV Care PN	-4,18 (-2,42- -5,93)	-2,66 (-0,25- -5,08)	-1,46 (3,13- -6,06)	2,86 (9,59- -3,87)
ANC	-4,79 (-3,08- -6,5)	-2,79 (-0,44- -5,14)	0,46 (5,5- -4,58)	5 (11,08- -1,09)
ANC PN	-6,84 (-5,26- -8,43)	-5,68 (-2,72- -8,64)	-0,03 (5,2- -5,25)	4,88 (12,04- -2,27)
FSW	-0,14 (1,7- -1,98)	0,29 (2,86- -2,29)	-2,1 (2,55- -6,74)	3,23 (10,71- -4,24)

The means and 95% CIs are calculated from the range of model outputs generated when the 100 best-fitting parameter combinations are entered into each model. The hypothetical implementation of STI screening programs is simulated as having been initiated in 2018, and the reduction in incidence and prevalence is calculated relative to the level that would have been expected in the absence of any screening program. Estimated trends for South Africa from 2018-2028.
* FSW = female sex worker, ANC = antenatal care

5. Standardised screening programme

Table S14: Sexual partner estimates and chlamydia prevalence

	Age (years)	CT prevalence prior to screening (%)	Average annual Partners	Effective Contact Rate *
**Females**	16 - 19	11.83 (11.46-12.2)	1.73 (1.73-1.74)	4.02 (4.01-4.02)
	20 - 24	15.34 (14.98-15.70)	2.68 (2.68-2.69)	4.02 (4.02-4.03)
	25 - 29	11.52 (11.25-11.78)	2.56 (2.55-2.56)	3.70 (3.70-3.71)
**Males**	16 - 19	4.30 (4.08-4.53)	0.89 (0.88-0.89)	3.51 (3.50-3.52)
	20 - 24	9.25 (8.91-9.59)	2.16 (2.15-2.17)	3.86 (3.85-3.87)
	25 - 29	9.93 (9.64-10.22)	2.59 (2.58-2.60)	3.80 (3.79-3.81)

The means and 95% CIs are calculated from the range of model outputs generated when the 100 best-fitting parameter combinations are entered into each model. The hypothetical implementation of STI screening programs is simulated as having been initiated in 2018.
* Effective contact rate (c), is calculated as $c = m + v/m$, where m is the mean number of sex partners per year and v its variance. This is a measure of the heterogeneity of sexual partnerships that can be compared across network models with different structures (Kretzschmar et al. 2009)
Estimated trends for South Africa from 2018-2028

Table S15: Chlamydia natural history parameters specified for individual-based stochastic simulation models of Chlamydia transmission

Parameter	RIVM	ClaSS	HPA	MicroCOSM
**Transmission probability per act of sex**				
Male-to-female	0.11	0.154	0.0375	0.162*
Female –to-male	0.11	0.122	0.0375	0.098*
**Frequency of sex acts**				
	1/day casual 0.25/day steady	0.5/day	1/day for 30 days then 0.25/day	3/month short-term 5/month long term (declining by 50% for every 20 -year increase in age)
**% of cases that become symptomatic**				
Male	0.50	0.75	0.00	0.37*
Female	0.30	0.30	0.045	0.11*
**Average duration asymptomatic infection (days)**				
Males	200	200	180	683*
Females	300	200	180	711*
**Average duration symptomatic infection (days)**				
Males	33	33	30	15*
Females	40	40	30	30*
**Proportion immune after treatment cure**	0	0	0	73.2*
**Average duration of immunity (days)**	0	0	0	2065*
**Pre-screening chlamydia prevalence (%)**				
Males (16-44 years old)	2.9	3.1	3.7	7.4 (7.2-7.6)**
Females (16-44 years old)	3.2	2.6	2.8	10.2 (9.9-10.4)**
**Chlamydia Gini Coefficient**	0.46	0.84	0.32	0.40***

* Median of 100 best fitting parameters
** Mean and 95% CIs calculated from the range of model outputs generated when the 100 best-fitting parameter combinations are entered into each model.
*** The Gini coefficient, a measure of the distribution of chlamydia infections among individuals with different levels of sexual activity, was calculated as previously described (Althaus et al. 2011), as a measure of the modelled heterogeneity in chlamydia risk.
Estimated trends for United Kingdom, Netherlands and South Africa after a hypothetical 10 year screening program

Table S16: Characteristics of individual-based stochastic simulation models of Chlamydia transmission used to simulate standardised screening program (Updated from Kretzschmar et al. (Kretzschmar et al. 2009))

RIVM model	ClaSS	HPA	MicroCOSM
**Simulation method**			
Discrete time step simulation. Movement between STI states are calculated at daily intervals.<br><br>Events occur each day with probabilities assigned or drawn from distributions			Discrete time step simulation. Movements between STI states are calculated at weekly intervals.
**Model population size**			
Closed population of 40 000	Closed population of 40 000	Closed population of 40 000	Population grows dynamically reaching a size of approximately 36 000 mid-way through the screening program
**Model population age**			
15-65 years<br>50% females	12-62 years<br>50% females	16-44 years<br>50% females	All ages. Projection begins with South Africa population profile in mid 1985, 50.39% births are male
**Sexual activity levels**			
Two risk groups<br><br>Core (5% of 15–35-year-old women and men)<br><br>Non-core (95% 15–35-year-old men and women, 100% 35+- year-old men and women)	Three risk groups<br><br>High (5% of 15–35-year-old women, 9% of 15–35-year-old men)<br><br>Medium (17% of 15–35-year-old women and men)<br><br>Low (78% of 15–35-year-old women, 74% of 15–35-year-old men)	Two risk groups<br><br>Core (propensity for short-term partnerships, initially 50% of 16-year-old women, 60% of 16-year-old men)<br><br>Non-core (prefer long-term partnership)<br><br>Each year 4% of men, 8% of women switch from core to non-core	Two risk groups<br><br>High (propensity for concurrency and commercial sex, 35% of males, 25% of females)<br><br>Low (no concurrent partnerships or commercial sex)<br><br>Sexual debut occurs between ages of 10-30, 50% higher rate in high-risk group.
**Partnership formation**			
Heterosexual only determined by sexual activity group, existing partnership status, age difference	Heterosexual only determined by sexual activity group, existing partnership status, age difference	Heterosexual only; determined by sexual activity group, existing partnership status, age difference<br>Two types of partnerships possible: short-term (core) and long-term (non-core).	Heterosexual only; determined by sexual activity group, existing partnership status and age of individual<br>Three types of partnerships possible: short-term (non-cohabiting), long-term (cohabiting or marital), sex worker-client
**Partnership duration**			
Mean 10 days for casual partnerships, 6.9 years (2519 days) for steady partnerships	Mean 950 days if both partners in lowest activity group; lower means for higher activity groups	Mean 14 days for short partnerships, 900 days for long partnerships and increases by 200 days each year	Mean 180 days for short term, rates of dissolution of long term partnerships based on age specific divorce rates
**Concurrent partnerships**			
Core group can have up to two partners<br><br>Non-core group has only one casual or steady partner at a time	Highest activity group can have more than two concurrent partners<br><br>Very low (but non-zero) probability of concurrent partnerships in lowest activity group	5% of population can have two partners until 35 years (first partnership may be short or long, second partner always casual)<br><br>All 35+ year olds prefer only one partner	High-risk individuals can have up to two partners. High-risk men can engage in commercial sex in addition to two concurrent partnerships.<br><br>Low-risk group are serially monogamous.
**Partner notification able to be modelled explicitly**			
Yes	Yes	Yes	Yes

References

Althaus CL, Turner KME, Mercer CH, et al (2014) Effectiveness and cost-effectiveness of traditional and new partner notification technologies for curable sexually transmitted infections: Observational study, systematic reviews and mathematical modelling. Health Technol Assess (Rockv) 18:1–99. doi: 10.3310/hta18020

Althaus CL, Turner KME, Schmid B V, et al (2011) Transmission of Chlamydia trachomatis through sexual partnerships: a comparison between three individual-based models and empirical data. J R Soc Interface 9:136–46. doi: 10.1098/rsif.2011.0131

Andersen B, Olesen F, Mller JK (2016) Population-Based Strategies for Outreach Screening of Urogenital Chlamydia trachomatis Infections : A Randomized , Controlled Trial Author (s): Berit Andersen , Frede Olesen , Jens K . Møller and Lars Østergaard Published by : Oxford University Press St. 185:252–258

Badman SG, Vallely LM, Toliman P, et al (2016) A novel point-of-care testing strategy for sexually transmitted infections among pregnant women in high-burden settings: results of a feasibility study in Papua New Guinea. BMC Infect Dis 16:1–6. doi: 10.1186/s12879-016-1573-4

Cabeza J, Garcia PJ, Segura E, et al (2015) Feasibility of Chlamydia trachomatis screening and treatment in pregnant women in Lima, Peru: a prospective study in two large urban hospitals. Sex Transm Infect 91:7–10. doi: 10.1136/sextrans-2014-051531

Cameron ST, Glasier A, Scott G, et al (2009) Novel interventions to reduce re-infection in women with chlamydia : a randomized controlled trial. 24:888–895. doi: 10.1093/humrep/den475

Centers for Disease Control and Prevention (2015) Sexually transmitted diseases treatment guidelines, 2015

Chen X-S, Yin Y-P, Chen L-P, et al (2006) Sexually transmitted infections among pregnant women attending an antenatal clinic in Fuzhou, China. Sex Transm Dis 33:296–301. doi: 10.1097/01.olq.0000194580.02116.6b

Cohen DA, Nsuami M, Martin DH, Farley TA (1999) Repeated school-based screening for sexually transmitted diseases: a feasible strategy for reaching adolescents. Pediatrics 104:1281–1285. doi: 10.1542/peds.104.6.1281

Department of Health Republic of South Africa SAHMS Final Report: Survey on female sex workers in South Africa 2013-2014

Department of Public Health Republic of South Africa (2015) National HIV Testing Services: Policy and Guidelines 2015. 1–46

Dinh T, Kamb ML, Msimang V, et al (2013) Integration of Preventing Mother-To-Child Transmission of HIV and Syphilis Testing and Treatment in Antenatal Care Services in the Northern Cape and Gauteng. Sex Transm Dis 40:846–851. doi: 10.1097/OLQ.0000000000000042

Estcourt CS, Sutcliffe LJ, Copas A, et al (2015) Developing and testing accelerated partner therapy for partner noti fi cation for people with genital Chlamydia trachomatis diagnosed in primary care : a pilot randomised controlled trial. Sex Transm Infect 91:548–554. doi: 10.1136/sextrans-2014-051994

Gaydos CA, Van Der Pol B, Jett-Goheen M, et al (2013) Performance of the cepheid CT/NG Xpert rapid PCR test for detection of Chlamydia trachomatis and Neisseria gonorrhoeae. J Clin Microbiol JCM-03461: doi: 10.1128/JCM.03461-12

Golden MR, Hogben M, Handsfield HH, et al (2003) Partner Notification for HIV and STD in the United States: Low Coverage for Gonorrhea , Chlamydial Infection and HIV. Sex Transm Dis 30:490–496

Golden MR, Whittington WLH, Handsfield HH, et al (2005) Effect of expedited treatment of sex partners on recurrent or persistent gonorrhea or chlamydial infection. N Engl J Med 352:676–685. doi: 10.1097/01.AOG.0000162921.49505.a1

Goldenkranz Salomon S, Fine D, Curtiss J, et al (2012a) Best practices in chlamydia (CT) and gonorrhea (GC) screening in a changing healthcare environment: Lessons from the Infertility Prevention Project (IPP). In: APHA Conference

Goldenkranz Salomon S, Fine D, Curtiss J, et al (2012b) Gonorrhea (GC) screening in a changing healthcare environment: Lessons from the Infertility Prevention Project (IPP). In: APHA Conference Proceedings

Goyal MK, Teach SJ, Badolato GM, et al (2016) Universal Screening for Sexually Transmitted Infections among Asymptomatic Adolescents in an Urban Emergency Department: High Acceptance but Low Prevalence of Infection. J Pediatr 171:128–132. doi: 10.1016/j.jpeds.2016.01.019

Herbst de Cortina S, Bristow CC, Joseph Davey D, Klausner JD (2016) A Systematic Review of Point of Care Testing for Chlamydia trachomatis, Neisseria gonorrhoeae, and Trichomonas vaginalis. Infect Dis Obstet Gynecol 2016:1–17. doi: 10.1155/2016/4386127

Herzog SA, Mcclean H, Carne CA, Low N (2011) Variation in partner notification outcomes for chlamydia in UK genitourinary medicine clinics : multilevel study. Sex Transm Infect 87:420–425. doi: 10.1136/sti.2011.049320

Hughes G, Ison C, Field N, et al (2014) Guidance for the detection of gonorrhoea in England. Public Hea:

Johnson LF, Alkema L, Dorrington RE, et al (2010) A Bayesian approach to uncertainty analysis of sexually transmitted infection models. Sex Transm Infect 86:169–174

Johnson LF, Dorrington RE, Bradshaw D (2011) The role of immunity in the epidemiology of gonorrhoea, chlamydial infection and trichomoniasis: insights from a mathematical model. Epidemiol Infect 139:1875–1883. doi: 10.1017/S0950268811000045

Johnson LF, Dorrington RE, Moolla H (2017) Progress towards the 2020 targets for HIV diagnosis and antiretroviral treatment in South Africa. South African J HIV Med 18:1–8

Johnson LF, Geffen N (2016) A Comparison of Two Mathematical Modeling Frameworks for Evaluating Sexually Transmitted Infection Epidemiology. Sex Transm Dis 43:139–46. doi: 10.1097/OLQ.0000000000000412

Kent CK, Branzuela A, Fischer L, et al (2004) Chlamydia and Gonorrhea Screening in San Francisco High Schools. Sex Transm Dis 29:373–375

Kissinger P, Mohammed H, Richardson-alston G, et al (2005) Patient-Delivered Partner Treatment for Male Urethritis : A Randomized , Controlled Trial. Clin Infect Dis 41:623–629

Kohler PK, Campos PE, Garcia PJ, et al (2016) STI Screening Uptake and Knowledge of STI Symptoms among Female Sex Workers Participating in a Community Randomized Trial in Peru. Int J STD AIDS 27:1–14. doi: 10.1038/nbt.3121.ChIP-nexus

Kretzschmar M, Turner KME, Barton PM, et al (2009) Predicting the population impact of chlamydia screening programmes: comparative mathematical modelling study. Sex Transm Infect 85:359–366. doi: 10.1136/sti.2009.036251

Low N, McCarthy A, Macleod J, et al (2007) Epidemiological, social, diagnostic and economic evaluation of population screening for genital chlamydial infection. Health Technol Assess (Rockv) 11:. doi:

10.2105/AJPH.2004.056044

Low N, Mccarthy A, Roberts TE, et al (2006) Partner notification of chlamydia infection in primary care: randomised controlled trial and analysis of resource use. BMJ 332:14–19. doi: 10.1136/bmj.38678.405370.7C

Low N, Redmond S, Uusküla A, et al (2016) Screening for genital chlamydia infection. Cochrane Database Syst Rev 2–5. doi: 10.1002/14651858.CD010866.pub2.

Marrazzo JM, Ellen JM, Kent C, et al (2007) Acceptability of urine-based screening for Chlamydia trachomatis to asymptomatic young men and their providers. Sex Transm Dis 34:147–153. doi: 10.1097/01.olq.0000230438.12636.eb

Miller CA, Tebb KP, Williams JK, et al (2015) Chlamydial Screening in Urgent Care Visits. 161:777–782

Monroe KW, Weiss HL, Jones M, Hook III EW (2003) Acceptability of urine screening for Neisseria gonorrheae and Chlamydia trachomatis in adolescents at an urban emergency department. Sex Transm Dis 30:850–853. doi: 10.1097/01.OLQ.0000086600.71690.14

Morton AN, Wakefield T, Tabrizi SN, et al (1999) An outreach programme for sexually transmitted infection screening in street sex workers using self-administered samples. Int J STD AIDS 10:741–743. doi: 10.1258/0956462991913286

Nelson HD, Zakher B, Cantor A, et al (2014) Screening for Gonorrhea and Chlamydia: Systematic Review to Update the U.S. Preventive Services Task Force Recommendations. Evid Synth Number 115:. doi: 10.7326/M14-1022

Ostergaard L, Andersen B, Olesen F, Moller JK (1998) Efficacy of home sampling for screening of Chlamydia trachomatis: randomised study. BMJ 317:26–27. doi: 10.1136/bmj.317.7150.26

Ramesh BM, Beattie TSH, Shajy I, et al (2010) Changes in risk behaviours and prevalence of sexually transmitted infections following HIV preventive interventions among female sex workers in five districts in Karnataka state, south India. Sex Transm Infect 86 Suppl 1:i17–i24. doi: 10.1136/sti.2009.038513

Ramjee G, Karim SSA, Sturm AW (1998) Sexually transmitted infections among sex workers in KwaZulu-Natal, South Africa. Sex Transm Dis 25:346–349

Richter M, Chersich MF, Vearey J, et al (2014) Migration status, work conditions and health utilization of female sex workers in three South African Cities. J Immigr Minor Heal 16:7–17. doi: 10.1007/s10903-012-9758-4

Shafer MB, Tebb KP, Pantell RH, et al (2002) Effect of a clinical practice improvement intervention on Chlamydial screening among adolescent girls. J Am Med Assoc 288:2846–2852. doi: 10.1001/jama.288.22.2846

South African Department of Health (2016) Implementation of the Universal Test and Treat Strategy for HIV Positive Patients and Differentiated Care for Stable Patients. Pretoria

Statistics South Africa. (2017) General Household Survey 2016

Takuva S, Brown AE, Pillay Y, et al (2017) The Continuum of HIV Care in South Africa: implications for achieving the second and third UNAIDS 90-90-90 targets. Aids 31:545–552. doi: 10.1097/QAD.0000000000001340

van de Laar MJW, Termorshuizen F, van den Hoek A (1997) Partner Referral by Patients With Gonorrhea and Chlamydial Infection. Sex Transm Dis 24:334–342

Wong HTH, Lee KCK, Chan DPC (2015) Community-Based Sexually Transmitted Infection Screening and Increased Detection of Pharyngeal and Urogenital Chlamydia trachomatis and Neisseria gonorrhoeae

Infections in Female Sex Workers in Hong Kong. Sex Transm Dis 42:185. doi: 10.1097/OLQ.0000000000000257
