# Supplementary material for: Modelling the impact of screening for chlamydia and gonorrhoea in youth and other high-prevalence groups in a resource-limited setting
Source: Int J Public Health. 2020 Apr 9;65(4):413–23. doi: 10.1007/s00038-020-01351-0 (PMC7274998; doi:10.1007/s00038-020-01351-0)
Supplement: Supplementary file 2 — Supplementary material 2 (PDF 1079 kb) [file 38_2020_1351_MOESM2_ESM.pdf]

Electronic Supplementary Material 2
Title: The impact of screening for chlamydia and gonorrhoea in youth and other high-prevalence groups in a resource-limited setting: insights from mathematical modelling
Journal: International Journal of Public Health
Authors:
Rachel T. Esra, MPH, University of Cape Town, South Africa
resra@myuct.ac.za
Dr Leigh F. Johnson, PhD, University of Cape Town, South Africa
leigh.johnson@uct.ac.za

Please note that Electronic Supplementary Material 2 is largely based on the original publication of the MircoCOSM model:

Johnson LF, Geffen N (2016) A Comparison of Two Mathematical Modeling Frameworks for Evaluating Sexually Transmitted Infection Epidemiology. Sex Transm Dis 43:139–46. doi: 10.1097/OLQ.0000000000000412

Online Resource 2

Contents

1. Model of sexual behaviour 3

   1.1 Structure of sexual behaviour model: risk groups and relationship types 3

   1.2 Rates of short-term partnership formation 5

   1.3 Rates of sexual debut 6

   1.4 Rates of marriage 7

   1.5 Mixing between risk groups 7

   1.6 Mixing between age groups 8

   1.7 Rates of partnership dissolution and divorce 9

   1.8 Commercial sex 9

   1.9 Frequency of sex 11

   1.10 Condom usage 11

   1.11 Partner matching algorithm 14

2. Mathematical modelling of STI transmission and natural history 16

   2.1 Mathematical model of gonorrhoea 16

   2.2 Mathematical model of chlamydial infection 17

   2.6 Mathematical model of HIV 18

   2.4 STI treatment 20

3. Modelling fitting procedure 21

4. Model fits to STI prevalence data 24

References 29

1. Model of sexual behaviour

The sections that follow describe the features of the sexual behaviour model in more detail. Most of this material has been published previously [1], but is reproduced here for convenience.

1.1 Structure of sexual behaviour model: risk groups and relationship types

The population is divided into two broad risk groups: a high risk group (representing individuals with a propensity for concurrent sexual partners and commercial sex) and a low risk group (representing individuals who do not engage in concurrent partnerships or commercial sex). Within each of these risk groups a number of sub-groups (or states) are defined, based on the individual's current relationship status; movements between these states occur as individuals form new partnerships and end previously-formed partnerships. Figure S1 illustrates the state space that is defined for women in the high risk group. The model distinguishes between short-term (non-cohabiting) and long-term (cohabiting or marital) relationships; in addition the model allows for once-off sex acts between sex workers and clients. All long-term relationships are assumed to start as non-cohabiting relationships. For the sake of simplicity, it is assumed that individuals in the high risk group do not have more than two partners at any point in time (although high risk men can have contact with sex workers if they have two current partners). It is also assumed in the interests of simplicity that individuals do not have more than one long-term partner at any point in time, as rates of polygamy in South Africa are relatively low [2]. By definition, individuals in the low risk group cannot have more than one partner at any point in time, and many of the states that are defined for the high risk group (shaded in grey in Figure S1) therefore do not apply to the low risk group. Women engaging in sex work are assumed not to form short-term or long-term relationships during the periods in which they are active as sex workers. Only heterosexual partnerships are considered.

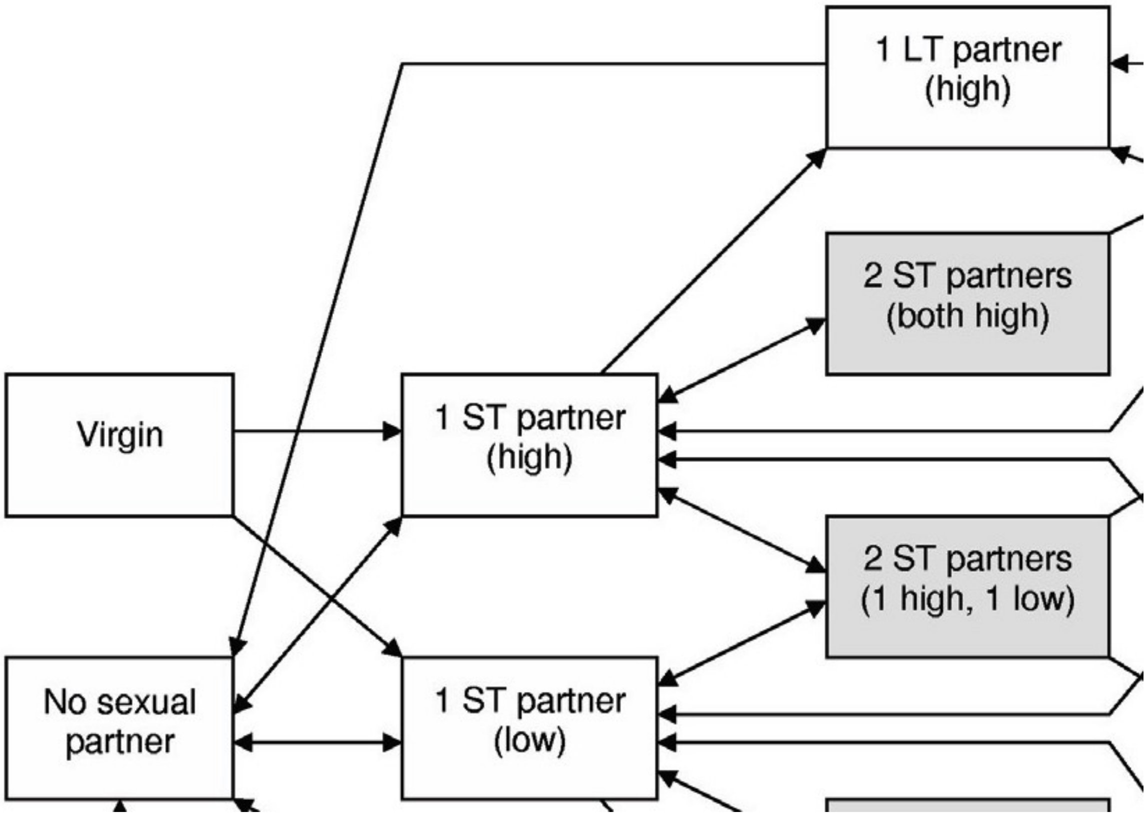

Figure S1: Multi-state model of sexual behaviour of 'high risk' females
LT = long-term (spousal). ST = short-term (non-spousal). 'High' and 'low' refer to the risk group of the sexual partner. The multi-state model for low risk females is the same as that shown here, except that the shaded states are omitted. The multi-state model for high risk men is also the same as that shown here, except that the 'sex worker' state is omitted.

The rates of transition between the different states differ according to the individual's age (rates are specified separately for each five-year age group). Some of the transition rates also depend on the individual's HIV status and stage of HIV disease. The variables used in classifying the behavioural states are summarized in Table S1. Throughout section 1, $N^{s}_{g,i,j,l}(x,t)$ represents the number of individuals in the population of age x (a 5-year interval), sex g, and risk group i, in relationship type l with a partner in risk group j, and in HIV disease stage s at time t.

Table S1: Index variables

Symbol	Definition	State space
i	Individual risk group	01 = virgin with propensity for concurrency<br>02 = virgin with no propensity for concurrency<br>1 = Sexually experienced, high risk<br>2 = Sexually experienced, low risk<br>3 = Commercial sex worker (relevant to females only)
j	Risk group(s) of partner(s)	0 = no partner; 1 = 1 high risk partner; 2 = 1 low risk partner; 11 = 2 high risk partners; 12 = primary high risk & secondary low risk; 21 = primary low risk & secondary high risk; 22 = 2 low risk partners*
l	Relationship type	1 = short-term (non-marital)<br>2 = long-term (marital)†
x	Individual age group	10, 15, 20, …, 85
y	Partner age	10, 15, 20, …, 85
g	Sex	1 = male; 2 = female
s	HIV disease state	0 = uninfected; 1 = acute HIV; 2 = asymptomatic HIV; 3 = WHO clinical stage 3; 4 = AIDS; 5 = on ART
t	Time	0 to 40 (in years from mid-1985)

ART = antiretroviral treatment.
* Where the individual is in a marital relationship with one partner and a non-marital relationship with another, the first index refers to the risk group of the spouse and the second refers to the risk group of the other partner.
† Where the individual has two partners, this index refers to the nature of the primary partnership (the secondary relationship is always short-term). Where the individual has no partners, the index is omitted.

The fraction of the population in the high risk group has been set at 35% for men and 25% for women, based on South African studies evaluating the fraction of individuals reporting concurrent partnerships [3, 4].

1.2 Rates of short-term partnership formation

The parameter $c^s_{g,i,j,l}(x)$ is defined as the annual rate at which a sexually-experienced individual of sex g wishes to form new short-term partnerships if they are in risk group i, aged x, in HIV disease state s, and in relationship type l with a partner in group j (if the individual is currently single, j = 0 and the l subscript is omitted). A gamma probability density function is used to represent age differences in rates of partnership formation; for the purpose of calculating a constant rate over a five-year age interval, x is taken as the mid-point of the age interval (e.g. 17.5 in the 15-19 year age group). The rate at which individuals wish to form new partnerships is calculated as

$$c^s_{g,i,j,l}(x) = c_g\left(x - 17.5\right)^{\alpha_g - 1}\exp\left(-\lambda_g\left(x - 17.5\right)\right)\Omega_{g,i,j,l}\Phi(s)$$

where c_g is the desired rate in the baseline group (single, HIV-negative individuals in the high risk group who are aged 15-19), λ_g and α_g are the parameters of the gamma probability density function, $\Omega_{g,i,j,l}$ is an adjustment factor taking into account the individual's risk group and current relationship status, and $\Phi(s)$ is an adjustment factor that takes into account the individual's HIV status. The values assumed in the model are summarized in Table S2. These parameter values were previously estimated by fitting a similarly-structured frequency-

dependent model to data on numbers of current sexual partners, by age and sex, in a nationally-representative 2005 survey [5]. (The calibration to sexual behaviour data made allowance for misreporting of partner numbers, as evidenced by inconsistencies in the numbers of current partners reported by men and women.) The sexual behaviour parameters were also partially determined based on the age and sex patterns of HIV prevalence in nationally representative household surveys and antenatal surveys [5, 6]. Full details regarding the model fitting procedure are provided elsewhere [7].

Table S2: Parameters determining rates of short-term partnership formation, in sexually-experienced adults

Parameter	Assumed value		Source/explanation
	Males	Females	
c_g	7.3	14.6	[8] for women, male rate assumed to be half of female rate
α_g	3.98	4.14	Calibrated
λ_g	0.1486	0.2272	Calibrated
$\Omega_{g,i,j,l}$ for i=1, if j=0	1	1	-
$\Omega_{g,i,j,l}$ for i=1, if l=1 and j=1 or 2	0.64	0.54	Calibrated [7]
$\Omega_{g,i,j,l}$ for i=1, if l=2 and j=1 or 2	0.41	0.17	Calibrated [7]
$\Omega_{g,i,j,l}$ for i=1, if j=11, 12, 21 or 22	0	0	Maximum of 2 current partners
$\Omega_{g,i,j,l}$ for i=2, if $j \neq 0$	0	0	Definition of low risk
$\Omega_{g,i,j,l}$ for i=2, if j=0	0.19	0.60	Calibrated [7]
$\Omega_{g,i,j,l}$ for i=3	0	0	No regular partners assumed for sex workers
$\Phi(0)$	1	1	-
$\Phi(1)$	1	1	No change in behaviour
$\Phi(2)$	1	1	assumed during early disease
$\Phi(3)$	0.65	0.65	[9-12]
$\Phi(4)$	0.25	0.25	[9-12]
$\Phi(5)$	0.80	0.80	[13, 14]

Because male and female demand for new partners may be inconsistent, it is necessary to balance the demand for new partnerships between the sexes. This balancing is achieved by randomly changing the order in which individuals choose their sexual partners, from one time step to the next, with individuals being more or less likely to achieve their desired number of partners depending on how close they are to the front of the 'queue'. This ensures that the actual number of new partnerships formed will be (on average) halfway between the number of new partnerships desired by men and the number of new partnerships desired by women.

1.3 Rates of sexual debut

Sexual debut is assumed to occur between the ages of 10 and 30. Sexual debut is assumed to occur upon entry into a short-term relationship, and the modelling of sexual debut is therefore similar to the modelling of the rates of short-term partnership formation. Rates of sexual debut are specified for each five-year age group, and correspond to the desired number of new sexual partners per period referred to in the previous section. The rates for the high risk group are specified in Table S3. Rates for the low risk group are assumed to be 50% of those in the high risk group, based on studies showing strong associations between early sexual

debut and high risk behaviour later in life [3, 15, 16]. The assumed rates of sexual debut have been set in such a way that the overall fraction of youth who are sexually experienced, by age and sex, are roughly consistent with those reported in a 2005 national survey [5], except in the case of girls, where a degree of under-reporting is assumed to occur.

Table S3: Annual rates of sexual debut in high risk youth, by age and sex

Age group	10-14	15-19	20-24	25-29
Males	0.01	0.27	0.82	1.00
Females	0.05	0.52	0.91	1.00

1.4 Rates of marriage

The rates at which short-term partnerships become cohabiting or marital have been set in such a way that the model matches the observed proportions of the population in marital/cohabiting relationships, by age and sex, as reported in national censuses in 1996 and 2001, and in a 2007 national community survey (more detail on the parameters and calibration is provided elsewhere [7]). The fraction married is assumed to be the same for the high risk group and low risk group, which means that the rate at which short-term relationships become marital must be higher in the low risk group in order to compensate for the lower numbers of short-term partnerships in the low risk group. The rates at which short-term relationships become marital are also assumed to depend on age and sex.

1.5 Mixing between risk groups

The parameter $\rho_{g,i,j}(t)$ is defined as the desired proportion of new short-term partners who are in risk group j, for an individual of sex g and in risk group i (j = 1 or 2 only) at time t. Mathematically, it is calculated according to the following formula:

$$\rho_{g,i,j}(t) = (1-\varepsilon)\delta_{ij} + \varepsilon \frac{\sum_{u=0}^{2}\sum_{l}\sum_{y}\sum_{s=0}^{5} N_{g^*,j,u,l}^{s}(y,t)c_{g^*,j,u,l}^{s}(y)}{\sum_{v=1}^{2}\sum_{u=0}^{2}\sum_{l}\sum_{y}\sum_{s=0}^{5} N_{g^*,v,u,l}^{s}(y,t)c_{g^*,v,u,l}^{s}(y)},$$

where δ_{ij} = 1 if $i = j$ and 0 otherwise, g^* is the sex opposite to g, and ε is the degree of assortative mixing. The degree of assortative mixing can be any value from 0 to 1, with lower values of the parameter indicating greater tendency to form partnerships with individuals in the same sexual activity class. To our knowledge, there are no South African data sources that directly inform the choice of the ε parameter. However, in a previous Bayesian analysis, which involved fitting the frequency-dependent model to South African HIV prevalence data and sexual behaviour data, the posterior mean of the ε parameter was 0.56 [7]. This parameter value has been used in the present analysis.

1.6 Mixing between age groups

For both sexes, an age mixing matrix is specified, which determines the fraction of partners in each five-year age group, for individuals in each age group. Tables S4 and S5 show the age mixing matrices for women and men respectively. The female age mixing matrix is estimated based on the ages of spousal partners reported by women in the 1998 Demographic and Health Survey [17] and the age differences reported by women in non-spousal partnerships in smaller studies [5, 18-20]. The male age mixing matrix has been calculated to be consistent with the female age mixing matrix. The age mixing matrices are taken into consideration when determining the relative probabilities of selecting different individuals as sexual partners (see section 1.11).

Table S4: Percentage of women's partners in each age group

	Age of male partner															
Female age	10-14	15-19	20-24	25-29	30-34	35-39	40-44	45-49	50-54	55-59	60-64	65-69	70-74	75-79	80-84	85+
10-14	46.8	43.1	8.1	1.5	0.3	0.1	0.0	0.0	0.0	0.0	0.0	0.0	0.0	0.0	0.0	0.0
15-19	0.0	47.5	40.9	8.8	2.1	0.5	0.0	0.1	0.0	0.0	0.0	0.0	0.0	0.0	0.0	0.0
20-24	0.0	0.0	48.9	35.2	11.1	3.6	0.8	0.3	0.0	0.1	0.0	0.0	0.0	0.0	0.0	0.0
25-29	0.0	0.0	1.3	43.8	35.7	12.7	4.4	1.0	0.3	0.3	0.1	0.2	0.1	0.0	0.0	0.0
30-34	0.0	0.0	0.2	3.3	37.5	38.4	13.3	4.1	1.4	0.9	0.2	0.4	0.1	0.0	0.0	0.0
35-39	0.0	0.0	0.0	0.3	3.9	37.2	36.3	14.0	4.4	1.4	0.9	1.2	0.3	0.0	0.0	0.0
40-44	0.0	0.0	0.0	0.1	1.4	5.3	35.4	35.3	15.6	3.7	1.2	1.6	0.4	0.1	0.0	0.0
45-49	0.0	0.0	0.0	0.0	0.0	1.2	9.2	35.3	31.9	14.2	3.8	3.4	0.9	0.1	0.0	0.0
50-54	0.0	0.0	0.0	0.0	0.0	0.0	1.6	14.4	32.1	29.8	15.3	5.2	1.3	0.2	0.0	0.0
55-59	0.0	0.0	0.0	0.0	0.0	0.0	0.0	2.0	15.8	33.6	29.7	13.9	4.0	0.8	0.1	0.0
60-64	0.0	0.0	0.0	0.0	0.0	0.0	0.0	0.0	1.8	15.9	36.5	30.9	11.9	2.5	0.3	0.0
65-69	0.0	0.0	0.0	0.0	0.0	0.0	0.0	0.0	0.0	1.4	16.2	41.2	31.4	8.7	1.0	0.1
70-74	0.0	0.0	0.0	0.0	0.0	0.0	0.0	0.0	0.0	0.0	1.0	17.4	47.6	29.1	4.6	0.2
75-79	0.0	0.0	0.0	0.0	0.0	0.0	0.0	0.0	0.0	0.0	0.0	0.6	20.3	56.5	21.3	1.2
80-84	0.0	0.0	0.0	0.0	0.0	0.0	0.0	0.0	0.0	0.0	0.0	0.0	0.7	26.7	57.9	14.8
85+	0.0	0.0	0.0	0.0	0.0	0.0	0.0	0.0	0.0	0.0	0.0	0.0	0.0	1.4	35.0	63.6

Table S5: Percentage of men's partners in each age group

	Age of female partner															
Male age	10-14	15-19	20-24	25-29	30-34	35-39	40-44	45-49	50-54	55-59	60-64	65-69	70-74	75-79	80-84	85+
10-14	100.0	0.0	0.0	0.0	0.0	0.0	0.0	0.0	0.0	0.0	0.0	0.0	0.0	0.0	0.0	0.0
15-19	10.8	89.2	0.1	0.0	0.0	0.0	0.0	0.0	0.0	0.0	0.0	0.0	0.0	0.0	0.0	0.0
20-24	0.8	30.7	66.5	1.8	0.2	0.0	0.0	0.0	0.0	0.0	0.0	0.0	0.0	0.0	0.0	0.0
25-29	0.1	5.6	40.9	49.8	3.3	0.2	0.1	0.0	0.0	0.0	0.0	0.0	0.0	0.0	0.0	0.0
30-34	0.0	1.4	13.5	42.5	38.7	3.0	0.9	0.0	0.0	0.0	0.0	0.0	0.0	0.0	0.0	0.0
35-39	0.0	0.4	4.7	16.4	43.0	31.2	3.6	0.7	0.0	0.0	0.0	0.0	0.0	0.0	0.0	0.0
40-44	0.0	0.0	1.2	6.9	18.0	36.6	29.6	6.7	1.0	0.0	0.0	0.0	0.0	0.0	0.0	0.0
45-49	0.0	0.1	0.6	1.8	6.4	16.3	34.0	29.6	10.0	1.2	0.0	0.0	0.0	0.0	0.0	0.0
50-54	0.0	0.0	0.1	0.7	2.6	6.3	18.4	32.9	27.3	11.0	0.7	0.0	0.0	0.0	0.0	0.0
55-59	0.0	0.0	0.2	0.9	2.2	2.5	5.4	18.4	32.0	29.7	7.9	0.5	0.0	0.0	0.0	0.0
60-64	0.0	0.0	0.0	0.4	0.8	2.2	2.4	6.4	21.4	34.2	23.8	8.2	0.3	0.0	0.0	0.0
65-69	0.0	0.0	0.0	0.9	1.6	3.4	3.7	7.0	8.7	19.2	24.3	25.3	5.8	0.0	0.0	0.0
70-74	0.0	0.0	0.0	0.4	0.7	1.5	1.7	3.1	3.7	9.6	16.1	33.1	27.3	2.7	0.0	0.0
75-79	0.0	0.0	0.0	0.2	0.3	0.6	0.6	1.1	1.8	4.7	8.3	22.5	40.9	18.3	0.8	0.0
80-84	0.0	0.0	0.0	0.0	0.0	0.0	0.0	0.0	1.4	3.6	5.4	13.6	32.4	34.7	8.7	0.2
85+	0.0	0.0	0.0	0.0	0.0	0.0	0.0	0.0	2.7	5.9	6.2	10.6	19.6	24.5	26.8	3.6

1.7 Rates of partnership dissolution and divorce

Partnerships can be terminated through death of either partner, through divorce (in the case of marital relationships) or through 'break up' (in the case of short-term relationships). In order to calculate rates of termination, it is necessary to define $D_{g,l}(x)$ as the annual rate at which partnerships of type l dissolve, among individuals aged x, of sex g (ignoring mortality). For short-term relationships ($l = 1$), the annual rate of dissolution has been set at 2, which implies an average duration of short-term relationships equal to 6 months, roughly consistent with average durations of 3-12 months observed in African studies of non-spousal relationships [8, 21, 22]. For long-term relationships ($l = 2$), the rates of relationship dissolution have been estimated by multiplying estimated rates of divorce in 2004 (by age and sex) by a factor of 2 [23]. (This upward adjustment makes allowance for the fact that rates of dissolution are higher in cohabiting non-marital relationships than in marital relationships, and many married individuals are separated although not formally divorced.) The age- and sex-specific rates of divorce are tabulated in a previous publication [7].

Relationships are also automatically terminated whenever one of the partners dies.

1.8 Commercial sex

Sexually experienced men are assumed to have contact with sex workers at an annual rate $w_{i,j,l}(x)$, which depends on their age (x), risk group (i) and current relationship status (represented by j and l). As in section 1.2, a gamma probability density function is used to represent the age differences in rates of male contact with sex workers; for the purpose of calculating a constant rate over each five-year age band, x is taken to be the midpoint of the age group (e.g. 17.5 for men aged 15 to 19). The following formula is used to calculate $w_{i,j,l}(x)$:

$$w_{i,j,l}(x) = w\lambda_c^{\alpha_c}(x-10)^{\alpha_c - 1}\exp\left(-\lambda_c(x-10)\right)Y_{i,j,l}$$

where λ_c and α_c are the parameters of the gamma probability density function, and $Y_{i,j,l}$ is an adjustment factor to represent the man's risk group and relationship status. The assumed values of the parameters are summarized in Table S6. The base rate of sex worker contact (w, which applies to high risk men who currently have no partner and are aged 20-24) has been set in such a way that the demand for commercial sex is sufficient to match the estimated size of the South African sex worker population in a recent study [24], when it is assumed that sex workers have 750 clients per annum on average [25-32]. (The resulting estimate of the fraction of women who are sex workers is lower than that estimated empirically [24] because the empirical estimate is based on a broader definition of sex worker than is used in the model.) The w parameter has been calculated so that the average number of sex worker contacts per year, averaged across all men aged 15 to 49 at the start of the simulation, is 5, i.e.

$$w \equiv \frac{5\sum_{x=1}^{7}\sum_{i}\sum_{j}\sum_{l}\sum_{s} N_{1,i,j,l}^{s}(x \times 5,0)}{\sum_{x=1}^{7}\sum_{j}\sum_{l}\sum_{s} N_{1,1,j,l}^{s}(x \times 5,0)\lambda_c^{\alpha_c}\left(x \times 5 + 2.5\right)^{\alpha_c - 1}\exp\left(-\lambda_c\left(x \times 5 + 2.5\right)\right)Y_{i,j,l}}$$

Table S6: Assumed male rates of sex worker contact

Parameter	Value	Source/explanation
λ_c	0.165	Based on age differences in rates of
α_c	3.31	male contact with sex workers [33]
$Y_{i,j,l}$ for i=1 and j=0	1	-
$Y_{i,j,l}$ for i=1, l=1 and j=1 or 2	0.5	Assumption
$Y_{i,j,l}$ for i=1, l=2 and j=1 or 2	0.3	Assumption
$Y_{i,j,l}$ for i=1, l=1 and j=11, 12, 21 or 22	0.2	Assumption
$Y_{i,j,l}$ for i=1, l=2 and j=11, 12, 21 or 22	0.1	Assumption
$Y_{i,j,l}$ for i=2	0	Definition of low risk group

Since only men in the high risk group are assumed to have contact with sex workers, and since all sex workers are assumed to be recruited from the high risk group, no assumptions about mixing between risk groups are required for the purpose of modelling commercial sex. It is assumed in the interests of simplicity that clients have no preference regarding the age of their commercial sex contacts, although age preferences are accounted for implicitly by assuming a sex worker age distribution (based on data from Johannesburg [34]) and setting the age-specific rates of entry into sex work in such a way that the simulated age distribution remains roughly stable over time and matches that assumed. Women are assumed to remain active as sex workers for two years on average, before returning to the 'No sexual partner' state (Figure S1).

The number of new sex workers required over the period $[t, t+d)$ in order to satisfy male demand, $\Delta_c(t, t+d)$, is calculated as

$$\Delta_c(t, t+d) = \frac{1}{C}\sum_{s=0}^{5}\sum_{j}\sum_{l}\sum_{x} N_{1,1,j,l}^{s}(x,t)w_{1,j,l}(x) - \sum_{s=0}^{5}\sum_{x} N_{2,3,0}^{s}(x,t),$$

where C is the assumed annual number of clients per sex worker (750), and the second term in the equation is the total number of sex workers at the start of the time step. The probability that a woman in the high risk group, who has no partners and is aged x and in HIV disease state s at time t, becomes a sex worker over the period $[t, t+d)$ is calculated as

$$\frac{\Delta_c(t, t+d)W(x)\Phi(s)}{\sum_{s=0}^{5}\sum_{u} N_{2,1,0}^{s}(u,t)W(u)\Phi(s)},$$

where $W(x)$ is the factor by which the rate of recruitment into the 'sex worker' group is multiplied when the woman is of age x in order to match the target sex worker age profile, and $\Phi(s)$ is the factor by which the rate of recruitment into commercial sex is adjusted in HIV

disease stage s (these are the same as the factors assumed in modelling formation of short-term partnerships – see Table S2).

1.9 Frequency of sex

In short-term relationships, sex is assumed to occur at a rate of 3 times per month on average, based on South African studies reporting on coital frequencies among youth [20, 35, 36]. In long-term relationships, coital frequencies are assumed to depend on the age and sex of the individual. In married women, coital frequencies are assumed to reduce exponentially from a rate of 5 per month in the 20-24 age group, declining by a factor of 50% for each 20-year increase in age (i.e. reducing to 2.5 times per month in 40-44 year olds, to 1.25 times per month in 60-64 year olds, etc.). Coital frequencies in married men are calculated to be consistent with the assumed female frequencies, taking into account the age mixing matrices. These assumed frequencies of sex in spousal and non-spousal partnerships result in numbers of sex acts that are roughly consistent with the aggregate reported coital frequencies in the 15-24 and 25-49 age bands in a 2005 national household survey [5]. As inconsistencies can emerge if coital frequencies are specified for men and women separately, the coital frequencies are simulated for men only.

1.10 Condom usage

The probability of condom use is assumed to depend on the individual's age and sex, as well as the type of relationship that they are in. In addition, the model allows for changes in condom usage over time, as evidence suggests that there have been substantial increases in condom usage since the launch of various HIV communication programmes in the 1990s and early 2000s [37, 38]. However, it has been noted that the actual trends in HIV prevalence in South Africa appear inconsistent with the reported increases in condom usage, and this suggests that there may be some degree of social desirability bias in the reporting of condom use [39]. In the calibration of the models to the HIV prevalence data, we therefore make allowance for uncertainty regarding the extent of the bias in the self-reported data, allowing certain parameters to be interpolated between minimum and maximum values (the maxima corresponding to values that would be assumed if there were no reporting bias). A detailed explanation of the model of condom use has been published previously [39], but is repeated here for convenience.

The parameter $\gamma_{2,l}(x,t)$ represents the probability that a woman aged x uses a condom in an act of sex with a partner of type l at time t. This parameter is calculated in relation to a 'baseline' rate of condom usage, γ^*, which is the probability of condom use for a woman aged 15-19 in a short-term relationship in 1998 (1998 has been chosen as the baseline because it is the year for which the most condom usage data are available, and because there is little reliable data on condom usage prior to 1998). The following formula is used to calculate $\gamma_{2,l}(x,t)$:

$$\ln\left(\frac{\gamma_{2,l}(x,t)}{1-\gamma_{2,l}(x,t)}\right) = \ln\left(\frac{\gamma^*}{1-\gamma^*}\right) + \chi_l + \nu_l(x-15) + \left[\kappa_l^i + (\kappa_l^u - \kappa_l^i)\left(1 - 0.5^{(t/M_l)^{Q_l}}\right)\right]$$

where

$\exp(\chi_l)$ = the odds of using a condom in relationship type *l*, relative to that in short-term relationhips (l = 1), in 1998;

$\exp(\nu_l)$ = the factor by which the odds of condom use reduces, per year of age;

$\exp(\kappa_l^i)$ = the initial odds of using a condom in relationship type *l*, in 1985 (before the onset of behaviour change), relative to the odds in 1998;

$\exp(\kappa_l^u)$ = the ultimate odds of using a condom in relationship type *l*, once behaviour change is at its maximum, relative to the odds in 1998;

M_l = the median time to behaviour change in relationships of type *l*, i.e. the time at which the log odds of condom use is half-way between its initial and ultimate levels (in years since 1985);

Q_l = the Weibull shape parameter controlling the speed of behaviour change in relationships of type *l*.

The term in square brackets represents the difference in condom usage (on a logit scale) between year *t* and 1998. A Weibull distribution is used to model the transition from the initial low levels of condom usage to the 'ultimate' levels of condom use. The logistic transformation prevents rates of condom use greater than 100%, and facilitates a 'logistic regression' interpretation of the condom parameters. Based on logistic regression models fitted to data on condom usage in the 1998 and 2003 South African DHSs [17, 40], it is assumed that the parameter ν_l is -0.025 for both spousal and non-spousal relationships, and that the odds of condom usage in spousal relationships relative to that in non-spousal relationships ($\exp(\chi_2)$) is 0.46 in 1998. The proportion of African women reporting condom usage for contraceptive purposes was found to be 0.13% in the 1987-89 DHS [41], compared to 1.8% in the 1998 DHS, and on the basis of this information, the ratio of the initial odds of condom use to that in 1998 ($\exp(\kappa_l^i)$) is assumed to be 0.07 for both spousal and non-spousal relationships.

In interactions between sex workers and their clients, levels of condom usage were around 60% in 1998 [18, 34], compared with levels of around 20% in women aged 15-19 in the 1998 DHS. Based on this evidence, it is assumed that in 1998 the ratio of the odds of condom use in sex worker-client interactions to that in non-spousal relationships ($\exp(\chi_3)$) was 6.0. In the absence of information regarding age differences in condom use by sex workers, no age effect is assumed (ν_3 = 0). A study conducted in 1988 found that condom usage was reported by only about 20% of sex workers and their clients [42], and the odds ratio for condom use in 1985, relative to that in 1998 ($\exp(\kappa_3^i)$), is therefore set at 0.17. More recent studies suggest that levels of condom usage close to 90% may be possible [30, 43, 44], and the ratio of the

ultimate odds of condom use to that in 1998 ($\exp(\kappa_3^u)$) is therefore set at 6.0. The parameter Q_3 has been set at 5.22, to produce a trend in condom use consistent with these survey estimates.

The remaining parameters - γ^*, κ_1^u, κ_2^u, Q_1 and Q_2 - have been set separately for two scenarios: a scenario in which women are assumed to report accurately on their levels of condom use, and a scenario in which women are assumed to overstate their levels of condom use substantially. A condom reporting bias parameter, θ, is used to interpolate linearly between the parameter values in these two scenarios, with $\theta = 0$ corresponding to the scenario in which there is no bias and $\theta = 1$ corresponding to the scenario in which there is substantial over-reporting of condom use. The assumed parameter values for the two scenarios are summarized in Table S7. Parameters in the 'no bias' scenario were chosen so that the modelled proportions of young women using condoms were reasonably consistent with data on the proportion of young women reporting having used a condom the last time they had sex [5, 17, 45-47]. Parameters in the 'high bias' scenario were chosen so that the modelled proportions of young women reporting condoms were consistent with proportions of sexually active women who reported using condoms for contraceptive purposes in the Demographic and Health Surveys (on the assumption that these would be less affected by social desirability bias and would represent a minimum on the true rate of condom use). Although the assumptions about the relative levels of condom usage in the early stages of the epidemic and the levels of condom usage in sex workers are the same in all scenarios, these parameters were found to have little influence on HIV incidence, and potential bias in the estimation of these parameters is therefore of little consequence.

Table S7: Differences in condom usage parameters between scenarios

Parameter	Symbol	No bias scenario ($\theta = 0$)	High bias scenario ($\theta = 1$)
Probability of condom use in women aged 15-19, in short-term relationships, in 1998	γ^*	0.20	0.08
Ultimate odds of condom use in short-term relationships, relative to the odds of condom use in 1998	$\exp(\kappa_1^u)$	15	3
Ultimate odds of condom use in long-term relationships, relative to the odds of condom use in 1998	$\exp(\kappa_2^u)$	7	1.5
Shape parameter controlling the speed of behaviour change in short-term relationships	Q_1	2.8	3.8
Shape parameter controlling the speed of behaviour change in long-term relationships	Q_2	1.8	3.6

For all relationship types, the median parameter M_l is calculated by noting that the 'baseline' parameters relate to 1998, and hence when $t = 13$ (i.e. in 1998)

$$\kappa_l^i + \left(\kappa_l^u - \kappa_l^i\right)\left(1 - 0.5^{(t/M_l)^{Q_l}}\right) = 0\,.$$

The parameter M_l is therefore calculated as a function of κ_l^i, κ_l^u and Q_l.

To ensure that male and female assumptions are consistent, the probability that a man uses a condom in a short-term or long-term relationship is calculated as

$$\gamma_{1,l}(x,t) = \sum_{y} f_1(y \mid x)\gamma_{2,l}(y,t),$$

where $f_1(y \mid x)$ is the probability that a female partner is aged y, if the male partner is aged x. The rate of condom use among clients of sex workers is the same as that estimated for sex workers, with no age dependency.

For the purpose of these analysis, the θ value is fixed at 0.63, the average of the parameters in the 100 best-fitting parameter combinations obtained when the model was previously calibrated to South African HIV prevalence data [1].

1.11 Partner matching algorithm

The defining feature of a network model is that it simulates pair formation and links an individual to a specific partner whenever a new partnership is formed. The procedure for pair formation is as follows:

1. At the start of each week, we calculate for each individual the rate at which they wish to form new partnerships (this calculation is described in section 1.2). Suppose that for the ith individual, c_i is the desired annual rate at which new partnerships are formed.
2. We then randomly generate a 'queue' of all individuals in the population (a new queue is randomly generated at the start of each time step, as a fixed queue would mean that some individuals are permanently advantaged/disadvantaged by their position in the queue).
3. For the first person in the queue, we randomly assign a new relationship status at the end of the week. Depending on the individual's relationship status at the start of the week, one of seven possible events can occur: they can acquire a new high risk partner, acquire a new low risk partner, marry an existing high risk partner, marry an existing low risk partner, end a short-term relationship with a high risk partner, end a short-term relationship with a low risk partner, or get divorced. If none of these events occur, the 'new' relationship status of the individual at the end of the week is the same as that at the start. The method for calculating the probabilities of each of these events is described in previous sections.
4. If the new event is acquisition of a new high risk partner, a partner age group is randomly sampled from the specified partner age preference matrix (see Tables S4 and S5). A new partner is selected from the pool of potential high risk partners in the relevant 5-year age group as follows:
   a) We calculate the sum of the rates at which individuals wish to form new partnerships, out of those people of the opposite sex in the high risk group who remain in the queue (i.e. individuals who have not yet been assigned a new relationship status). Mathematically, we are calculating for age group x,

$$N(x) = \sum_{j \in J(x)} c_j ,$$

where $J(x)$ is the set of high risk individuals aged x, of the opposite sex, who remain in the queue, and c_j is the annual rate at which individual j wishes to acquire new partners.

b) We assign sample weights to each of the individuals who is eligible to form a new relationship with the first individual. Mathematically, the weight assigned to individual j, if they are in the set $J(x)$, is $w_j = c_j / N(x)$.

c) A new partner is randomly selected from the set $J(x)$ using the sample weights. If set $J(x)$ is empty, a different age group is randomly selected, and a high risk partner from that risk group is randomly chosen, in the same way as before. However, if the second age group is also empty, the event assigned to individual i changes to acquisition of a new partner in the low risk group, and a partner is selected from the low risk group in the same way as for the high risk group (using the same sampled age groups). If there are no available partners in the low risk group, for either of the randomly sampled ages, the individual is assigned no change in relationship status.

The procedure is exactly the same if the new event is acquisition of a new low risk partner, except that the 'high' and 'low' risk labels in steps a-c are reversed.

5. The procedure outlined in steps 3 and 4 is repeated for the second person in the queue, and similarly for each subsequent individual. Note that each time an individual has a change in relationship status assigned to them, the relationship status of the associated partner automatically also gets updated, so that the partner gets removed from the queue of individuals waiting to be assigned a new relationship status.

The removal from the queue in the last step happens because we are only allowing a maximum of one new relationship event per individual, in each time step. For example, if individual A selects individual B as their new partner, then individual B has had an event assigned to them and cannot experience another event in the same time step. Similarly, if individual A is in a relationship with individual C at the start of the time step and does not end that relationship, then individual C cannot end the relationship when their turn in the queue comes (otherwise individual A would be both forming a new partnership and ending a partnership in the same time step). Because we are only allowing one event to occur in each time step, we avoid having to make assumptions about how partnership allocation is ordered when multiple partnerships can be assigned [48]. Although the assumption that only one partnership event can occur in each time step is not realistic, we are using weekly time steps to model changes in relationship status, and any loss of accuracy is therefore likely to be minimal. Contacts between sex workers and clients are not included in these relationship events.

Although sexual relationships are updated at weekly time steps, STI transmission and resolution can be updated more frequently. In the analyses that follow, HIV transmission and disease progression are updated at weekly time steps, but all other STIs are updated four times per week (to account for the rapid health seeking that may occur when STI symptoms develop).

2. Mathematical modelling of sexually transmitted infection transmission and natural history

The sections that follow describe the modelling of the natural history of each STI and the assumptions about transmission probabilities per act of unprotected sex. Prior distributions are specified to represent the uncertainty around certain parameters, and in section 3 we explain the procedure for sampling from these distributions to generate the 100 parameter combinations that are used in the main text. The model assumptions and prior distributions are the same as described previously [1].

2.1 Mathematical model of gonorrhoea

Individuals who acquire gonorrhoea are assumed to either develop symptoms or remain asymptomatic, and eventually experience spontaneous resolution of infection if treatment is not sought. Symptomatic individuals are assumed to seek treatment at rate υ, which is effective in curing the infection with probability ψ. As there is some evidence of strain-specific immunity following recovery from gonorrhoea [49, 50], an additional state is defined to represent individuals who are temporarily immune following recovery (individuals are assumed to be completely protected against reinfection while in this state). All individuals who experience spontaneous resolution of infection are assumed to enter this state. However, since successful early treatment of gonorrhoea does not appear to be followed by immunity [51], only a fraction (φ_2) of individuals are assumed to be immune if they have experienced resolution of infection following treatment. Immunity is assumed to wane at rate σ_3. This model of natural history and immunity is illustrated in Figure S2.

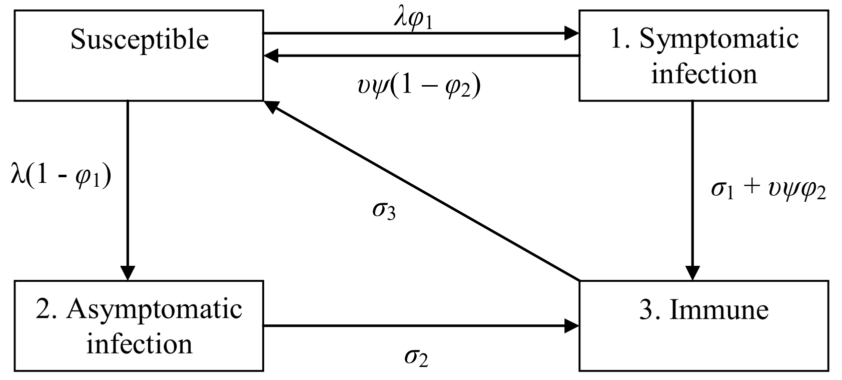

Figure S2: Multi-state model of the natural history of gonorrhoea

The prior distributions for the associated model parameters are specified in Table S8. For the most part these are the same as assumed in earlier iterations of the model [52, 53], but the prior distribution for the average duration of untreated gonorrhoea has a greater mean and standard deviation than that assumed previously, due to the recognized limitations of the available data [54]. Due to the lack of empirical data on transmission probabilities per act of

sex, we have relied on estimates of transmission probabilities from other studies that have fitted mathematical models to gonorrhoea prevalence data.

Table S8: Gonorrhoea parameters

Parameter	Symbol	Prior distribution Type	Mean	SD	Ref.
% of cases that become symptomatic					
Male	φ_1	Beta	0.90	0.05	[51, 55-59]
Female		Beta	0.40	0.15	[60, 61]
Average duration if untreated (weeks)					
Male	$1/\sigma_2$*	Gamma	20.0	10.0	[54, 61, 62]
Female		Gamma	20.0	10.0	[54, 61]
Average duration of immunity (weeks)	$1/\sigma_3$	Gamma	52.0	26.0	-
Proportion immune after treatment cure	φ_2	Uniform	0.50	0.29	-†
Transmission probability per act of sex					
Male-to-female	-	Beta	0.40	0.10	[60, 63-67]
Female-to-male	-	Beta	0.20	0.05	[64-69]
Fraction of symptoms correctly treated prior to introduction of SM	A	Beta	0.70	0.10	[70-73]

* Same parameter is used for symptomatic duration ($1/\sigma_1$) and asymptomatic duration ($1/\sigma_2$). † Due to the lack of evidence, a vague prior (uniform on the interval [0, 1]) is assumed.
SD = standard deviation, SM = syndromic management.

2.2 Mathematical model of chlamydial infection

The model of chlamydial infection is identical in structure to that used for gonorrhoea (see Figure S2), but parameters differ. As there is substantial evidence of partial immunity to chlamydial infection following recovery [74-78], a longer average duration of immunity is assumed. Since immunity is thought to be more significant when treatment is initiated in late disease than in early disease [79-81], it is assumed that only a proportion φ_2 of those who are successfully treated acquire immunity (since treated symptomatic individuals would tend to have a shorter duration of infection than individuals who experience spontaneous resolution). The prior distributions assigned to the various parameters are shown in Table S9; these are the same as the prior distributions assumed previously [1, 52].

Table S9: Parameters for chlamydial infection

Parameter	Symbol	Prior distribution Type	Mean	SD	Ref.
% of cases that become symptomatic					
Male	φ_1	Beta	0.30	0.15	[56, 58, 61, 82]
Female		Beta	0.15	0.08	[56, 61]
Average duration if untreated (weeks)					
Symptomatic	$1/\sigma_1$	Gamma	16.0	5.0	[61, 83]
Asymptomatic	$1/\sigma_2$	Gamma	90.0	15.0	[84-86]
Average duration of immunity (weeks)	$1/\sigma_3$	Gamma	520	200	-
Proportion immune after treatment cure	φ_2	Uniform	0.50	0.29	-*
Transmission probability per act of sex					
Male-to-female	-	Beta	0.12	0.06	[64-66]
Female-to-male	-	Beta	0.16	0.10	[64-66]
Fraction of symptoms correctly treated prior to introduction of SM	A	Beta	0.70	0.10	[70-73]

* Due to the lack of evidence, a vague prior (uniform on the interval [0, 1]) is assumed.
SD = standard deviation, SM = syndromic management.

2.6 Mathematical model of HIV

A four-stage model is used to describe the course of HIV disease in the absence of antiretroviral treatment (ART), with σ_i representing the weekly rate of transition out of stage i (in the absence of ART) and β_i representing the weekly rate of HIV transmission from infected individuals in stage i to susceptible partners. Individuals who develop AIDS-related symptoms are assumed to start ART with probability φ, but φ can increase above 1 if the number of individuals starting ART exceeds the number of individuals progressing to AIDS (Figure S3).

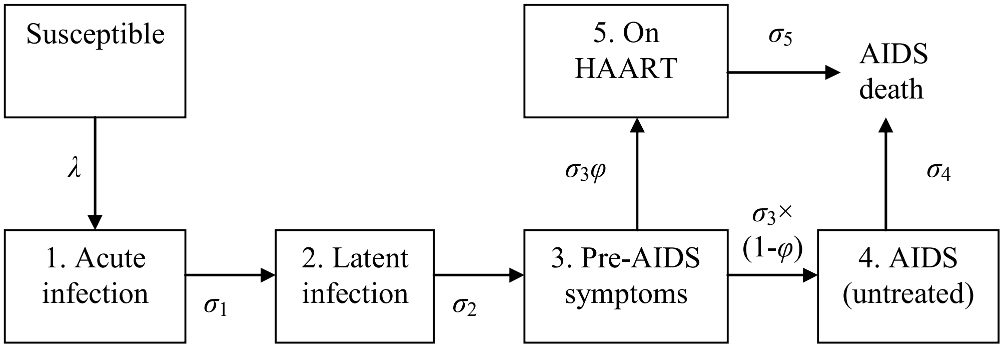

Figure S3: Model of HIV/AIDS
In cases where the ratio of ART initiates to new AIDS cases (φ) exceeds 1, the rate of transition from pre-AIDS to AIDS is set to 0.

Table S10 summarizes the values assigned to the parameters in the HIV model. Some of these are estimated directly from the cited sources, while others are the average values from the 100 parameter combinations that previously gave the best fit to South African HIV prevalence data [1]. The relative infectiousness during the pre-AIDS symptomatic stage is

assumed to be intermediate between that in the asymptomatic stage and that in the untreated AIDS stage, with $\beta_3/\beta_2 = \sqrt{\beta_4/\beta_2}$. HIV transmission probabilities per act of unprotected sex are specified separately for short-term and long-term relationships.

Table S10: Prior distributions for parameters in HIV model

Parameter	Symbol	Value	Ref.
Average time spent in absence of ART (years)			
Acute infection	$1/\sigma_1$	0.25	[87]
Latent infection	$1/\sigma_2$	5.16	[88, 89]
Pre-AIDS symptoms	$1/\sigma_3$	4.14	[88, 89]
AIDS	$1/\sigma_4$	1.96	[88, 89]
Average annual AIDS mortality after ART start	σ_5	0.033	[90]
Relative infectiousness during acute HIV	β_1/β_2	19.5	[87, 91, 92]
Relative infectiousness during untreated AIDS	β_4/β_2	6.9	[93]
Transmission probability: non-spousal partners*			
Client-to-sex worker	-	0.003	[94-97]
Male-to-female (ST relationships)	-	0.0083	[98, 99]
Female-to-male	-	0.0036	[100, 101]
Transmission probability: spousal partners*			
Male-to-female	-	0.0020	[102-104]
Female-to-male	-	0.0017	[102-104]
HIV prevalence in high risk females in 1990	V_0	0.0217	[105, 106]

* Average across untreated disease stages, using average time spent in each stage as weights.
SD = standard deviation.

V_0 is the initial HIV prevalence in high risk females aged 15-49 in 1990 (although the simulation begins in 1985, it is convenient to start the simulation of HIV transmission in 1990 because stochastic variation in HIV trajectories is less substantial when the HIV epidemic is initialized using a higher initial HIV prevalence). This initial HIV prevalence is adjusted by a set of scaling factors to determine the initial HIV prevalence by sex and by 5-year age group, based on the relative levels of HIV prevalence in males and females in different age groups in a 1991 survey in KwaZulu-Natal [107]. Suppose that $s_g(x)$ represents the scaling factor for high risk individuals of age *x* and sex *g*, in the high risk group, and that $v_{g,r}(x)$ represents the initial HIV prevalence (in 1990) in individuals of sex *g* and risk group *r*, who are aged *x*. We calculate $v_{g,r}(x)$ as

$$v_{g,r}(x) = \begin{cases} V_0 s_g(x) & \text{for } r = 1 \text{ (high risk)} \\ 0 & \text{for } r = 2 \text{ (low risk)} \end{cases}$$

The values assumed for the $s_g(x)$ scaling factors, based on the KwaZulu-Natal survey, are summarized in Table S11.

Table S11: Assumed ratios of initial HIV prevalence to average prevalence in females aged 15-49 (high risk group)

	15-19	20-24	25-29	30-34	35-39	40-44	45-49
Males (g = 0)	0.04	0.44	1.00	0.69	0.58	0.48	0.29
Females (g = 1)	1.11	1.24	1.24	0.95	0.68	0.54	0.38

2.4 Sexually transmitted infection treatment

The modelling of STI treatment has been described previously [108]. In the case of genital ulcer and discharge symptoms, it is assumed that the rate of health seeking (υ) depends on the individual's age and sex: for adults aged 20 and older, the weekly rate of seeking treatment is 0.23 in women and 0.57 in men [18, 109-111], and these rates are halved in adolescents [112, 113]. In sex workers the weekly rate of health seeking is assumed to be 0.9 [18, 113].

Rates of STI cure are determined by defining the following symbols:

$\psi_g^d(t)$ = probability that an individual of sex *g*, experiencing symptoms of disease *d*, is cured if they seek treatment at time *t*

$r_{g,h}$ = % of individuals of sex *g* who seek STI treatment in health sector *h* (*h* can take on values of 0, 1 or 2, corresponding to the public health sector, formal private health sector and traditional healers respectively)

$\Omega_h(t)$ = % of health workers in sector *h* who correctly follow syndromic management protocols at time *t* (*h* = 0 or 1 only)

A_g^d = % of individuals of sex *g*, with symptoms of STI *d*, who receive effective treatment if the health worker is *not* following syndromic management protocols (ignoring the potential effect of drug shortages)

Z_g^d = % of individuals of sex *g*, with symptoms of STI *d*, who receive effective treatment if the health worker is following syndromic management protocols (ignoring the potential effect of drug shortages)

$V(t)$ = % reduction in the probability of cure in public STI clinics as a result of drug shortages, at time *t*

ς_A^d = probability that STI *d* is cured if treated with effective drugs

ς_T^d = probability that STI *d* is cured if treated by a traditional healer

The probability of cure is then calculated as:

$$\psi_g^d(t) = \big[r_{g,0}\big((1-\Omega_0(t))A_g^d + \Omega_0(t)Z_g^d\big)(1-V(t)) \\ + r_{g,1}\big((1-\Omega_1(t))A_g^d + \Omega_1(t)Z_g^d\big)\big]\varsigma_A^d + r_{g,2}\varsigma_T^d$$

This is the weighted average probability of cure in the different health sectors, where the weights are the proportions of individuals seeking treatment in each sector, $r_{g,h}$.

Of men who seek treatment, 45% are assumed to seek treatment in the public health sector, 40% seek treatment in the formal private sector and the remaining 15% seek treatment from traditional healers [18, 109, 114-116]. For women, the corresponding proportions are 60%, 30% and 10% respectively. For the formal health sectors, probabilities of effective treatment being provided prior to syndromic management (A_g^d) are difficult to determine precisely, and these parameters have therefore been assigned prior distributions to represent the associated uncertainty (see Tables S8 and S9). Probabilities of cure, if effective treatment is provided,

have been set to 90% for most STIs [108]. For the informal health sector, treatment is assumed not to be effective (ς_T =0).

The time-varying parameters are summarized in Table S12. Rates of ART initiation are the same as those assumed previously [117]. In the case of gonorrhea, probabilities of treatment success have been adjusted to take into account rising levels of ciprofloxacin resistance in recent years [118-120]: the treatment effectiveness parameter (ς_A) is reduced in proportion to the product of the fraction of gonorrhoea cases treated with ciprofloxacin and the fraction of gonorrhoea cases that are ciprofloxacin-resistant (Table S12). This adjustment ceases to apply after 2008, when syndromic management guidelines were revised to recommend the use of ceftriaxone in place of ciprofloxacin [121].

Table S12: Time-varying treatment parameters

Year	% of providers correctly using syndromic management protocols		% of public clinics with STI drug shortages	Source	Ratio of new ART patients to new AIDS cases	% of gonorrhoea cases that are ciprofloxacin-resistant	% of gonorrhoea cases that are treated with ciprofloxacin
	Private	Public					
1993	0%	0%	20%		0.000	0%	50%
1994	3%	10%	20%		0.000	0%	50%
1995	7%	30%	18%		0.000	0%	50%
1996	11%	50%	16%	[122][a]	0.000	0%	50%
1997	15%	65%	13%	[123][b]	0.000	0%	50%
1998	18%	75%	10%	[124][a]	0.000	0%	50%
1999	21%	78%	8%	[73][b]	0.000	0%	50%
2000	23%	80%	6%		0.035	0%	50%
2001	25%	80%	5%	[125][b]	0.046	0%	50%
2002	27%	80%	4%	[126][a]	0.048	0%	50%
2003	29%	80%	4%	[127][a]	0.085	4%	50%
2004	31%	80%	4%		0.220	10%	50%
2005	33%	80%	4%		0.402	16%	50%
2006	35%	80%	4%		0.480	25%	50%
2007	37%	80%	4%		0.650	38%	50%
2008	39%	80%	4%		1.004	55%	50%
2009	41%	80%	4%		1.290	70%	0%
2010	43%	80%	4%		1.656	82%	0%
2011	45%	80%	4%		1.656	90%	0%
2012	47%	80%	4%		1.656	96%	0%

[a] Public health sector. [b] Private health sector.

3. Modelling fitting procedure

The models are fit separately for each STI, using the sexual behaviour assumptions described previously. The approach to defining the likelihood for STIs is the same as that described previously [52], with some modifications to the data sources used. Briefly, a beta-binomial distribution is used to model the observed variation in the fraction of individuals testing positive for the STI of interest in different studies. The beta distribution parameters are set to take into account both (a) the expected variation in the true STI prevalence between different studies populations and (b) the expected variation in the performance (sensitivity and specificity) of the diagnostic method used in the study of interest. Although we previously specified a prior distribution to represent the variance in respect of (a), we have fixed these

variance parameters at the posterior means estimated previously [52] for the purpose of the present analysis. The calibration procedure has been shown to have good validity [52].

The data sources on which the likelihood values are based are summarized in Tables S13 and S14.

Table S13: Gonorrhoea prevalence estimates

Study	Year	Sample	Location	n	Prev.	Diagnostic
Hoosen *et al* [128]	-	ANC	Durban	32	6.0%	Culture
Govender *et al* [129]	1994-5	ANC	Durban	168	3.0%	Culture
Kharsany *et al* [130]	1994	ANC	Durban	52	5.8%	Culture
Rours *et al* [131]	1996-7	ANC	Johannesburg	766	8.5%	LCR on urine
Sturm *et al* [132]	1996	ANC	Hlabisa	327	7.8%	Culture
Sturm *et al* [133]	1999	ANC	Hlabisa	245	7.0%	PCR on swabs
Sturm *et al* [134]	-	ANC	Hlabisa	185	7.6%	-*
Sturm *et al* [133]	2002	ANC	Hlabisa	449	4.0%	PCR on swabs
Odendaal *et al* [135]	2002	ANC	Cape Town	343	0.9%	Culture
Frohlich *et al* [136]	2002	ANC	Vulindlela	48	4.2%	SDA on swabs
Moodley *et al* [137]	2008-10	ANC	Durban	1459	6.4%	SDA on swabs
Ramjee *et al* [94]	1996-2000	CSW	KZN	387	10.3%	Culture
Steen *et al* [138]	1996-7	CSW	Virginia	407	17.3%	LCR on urine
Dunkle *et al* [28]	1996-7	CSW	Johannesburg	295	23.3%	LCR on urine
Williams *et al* [18]	1998	CSW	Khutsong	121	15.7%	LCR on urine
Williams *et al* [139]	2000	CSW	Khutsong	93	16.1%	LCR on urine
Vickerman *et al* [140]	2000	CSW	Johannesburg	310	25%	LCR on urine
Ndhlovu *et al* [116]	2001	CSW	Khutsong	101	10.0%	LCR on urine
Schneider *et al* [141]	1994	FPC	Bushbuckridge	249	3.0%	LCR on urine
Wilkinson *et al* [142]	-	FPC	Hlabisa	189	4.0%	Culture
Kharsany *et al* [130]	1994	FPC	Durban	55	5.5%	Culture
Hoosen *et al* [143]	-	FPC	Durban	40	5.0%	Culture
Fehler *et al* [144]	-	FPC	Johannesburg	210	8.6%	LCR on urine
Kleinschmidt *et al* [145]	1999-2001	FPC	Orange Farm	538	3.9%	LCR on urine
Frohlich *et al* [136]	2002	FPC	Vulindlela	226	6.6%	SDA on swabs
Colvin *et al* [146]	1995	HH, F 15-49	Hlabisa	137	5.8%	LCR on urine
Williams *et al* [18]	1998	HH, F 15-59	Khutsong	712	6.9%	LCR on urine
Auvert *et al* [98]	1999	HH, F 15-24	Khutsong	622	10.9%	LCR on urine
Williams *et al* [139]	2000	HH, F 15-49	Khutsong	893	8.6%	LCR on urine
Ndhlovu *et al* [116]	2001	HH, F 15-59	Khutsong	878	11.0%	LCR on urine
Pettifor *et al* [147]	2002-3	HH, F 15-19	Peri-urban	2624	3.5%	PCR on urine
		HH, F 20-24	townships	2002	3.5%	PCR on urine
Hurkchand *et al* [148]	2002	HH, F 20-49	Mbalenhle	399	4.7%	PCR on urine
O'Leary *et al* [149]	2010	HH, F 15-19	Eastern Cape	329	10.6%	Aptima on urine
Colvin *et al* [146]	1995	HH, M 15-49	Hlabisa	85	2.4%	LCR on urine
Williams *et al* [18]	1998	HH, M 15-59	Khutsong	475	3.4%	LCR on urine
Auvert *et al* [98]	1999	HH, M 15-24	Khutsong	560	2.9%	LCR on urine
Williams *et al* [139]	2000	HH, M 15-49	Khutsong	606	3.3%	LCR on urine
Ndhlovu *et al* [116]	2001	HH, M 15-59	Khutsong	532	4.0%	LCR on urine
Pettifor *et al* [147]	2002-3	HH, M 15-19	Peri-urban	2389	1.1%	PCR on urine
		HH, M 20-24	townships	1455	3.2%	PCR on urine
Hurkchand *et al* [148]	2002	HH, M 20-49	Mbalenhle	291	3.9%	PCR on urine
O'Leary *et al* [149]	2010	HH, M 15-19	Eastern Cape	330	1.8%	Aptima on urine

* Diagnosed by culture and a series of genetic tests, which in combination would have had very high sensitivity and specificity.

ANC = antenatal clinic attenders. CSW = commercial sex workers. F = females. FPC = family planning clinic attenders. HH = households. KZN = KwaZulu-Natal. LCR = ligase chain reaction test. M = males. PCR = polymerase chain reaction test. Prev. = prevalence. SDA = strand displacement amplification.

Table S14: Chlamydial infection prevalence estimates

Study	Year	Sample	Location	n	Prev.	Diagnostic
Hoosen *et al* [128]	-	ANC	Durban	32	41.0%	DIF
Kharsany *et al* [130]	1994	ANC	Durban	52	19.2%	DIF
Rours *et al* [131]	1996-7	ANC	Johannesburg	766	12%	LCR on urine
Sturm *et al* [132]	1996	ANC	Hlabisa	327	12.9%	DIF
Sturm *et al* [133]	1999	ANC	Hlabisa	245	11.0%	PCR on swabs
Sturm *et al* [134]	-	ANC	Hlabisa	185	13.5%	-*
Sturm *et al* [133]	2002	ANC	Hlabisa	449	11.0%	PCR on swabs
Odendaal *et al* [135]	2002	ANC	Cape Town	343	11.7%	PCR on swabs
Frohlich *et al* [136]	2002	ANC	Vulindlela	48	8.3%	SDA on swabs
Govender *et al* [150]	2005	ANC	Cape Town	219	18.7%	PCR on swabs
Moodley *et al* [137]	2008-10	ANC	Durban	1459	17.8%	SDA on swabs
Ramjee *et al* [27]	1996-7	CSW	KZN	145	16.4%	DIF
Steen *et al* [138]	1996-7	CSW	Virginia	407	14.3%	LCR on urine
Dunkle *et al* [28]	1996-7	CSW	Johannesburg	295	8.4%	LCR on urine
Williams *et al* [18]	1998	CSW	Khutsong	121	9.1%	LCR on urine
Williams *et al* [139]	2000	CSW	Khutsong	93	12.9%	LCR on urine
Vickerman *et al* [140]	2000	CSW	Johannesburg	310	17%	LCR on urine
Ndhlovu *et al* [116]	2001	CSW	Khutsong	101	8.0%	LCR on urine
Schneider *et al* [141]	1994	FPC	Bushbuckridge	249	12.0%	LCR on urine
Wilkinson *et al* [142]	-	FPC	Hlabisa	189	8.0%	DIF
Kharsany *et al* [130]	1994	FPC	Durban	55	12.7%	DIF
Hoosen *et al* [143]	-	FPC	Durban	40	15.0%	DIF
Fehler *et al* [144]	-	FPC	Johannesburg	210	18.1%	LCR on urine
Kleinschmidt *et al* [145]	1999-2001	FPC	Orange Farm	539	14.1%	LCR on urine
Frohlich *et al* [136]	2002	FPC	Vulindlela	226	8.8%	SDA on swabs
Colvin *et al* [146]	1995	HH, F 15-49	Hlabisa	140	6.4%	LCR on urine
Williams *et al* [18]	1998	HH, F 15-59	Khutsong	712	8.1%	LCR on urine
Auvert *et al* [98]	1999	HH, F 15-24	Khutsong	622	14.6%	LCR on urine
Williams *et al* [139]	2000	HH, F 15-49	Khutsong	893	13.8%	LCR on urine
Ndhlovu *et al* [116]	2001	HH, F 15-59	Khutsong	878	12.0%	LCR on urine
Auvert et al [151]	2002	HH, F 15-49	Orange Farm	492	6.9%	PCR on urine
Pettifor *et al* [147]	2002-3	HH, F 15-19	Semi-urban	2624	9.1%	PCR on urine
		HH, F 20-24	townships	2002	10.8%	PCR on urine
Hurkchand *et al* [148]	2002	HH, F 20-49	Mbalenhle	399	6.5%	PCR on urine
O'Leary *et al* [149]	2010	HH, F 15-19	Eastern Cape	329	23.1%	Aptima on urine
Colvin *et al* [146]	1995	HH, M 15-49	Hlabisa	90	5.6%	LCR on urine
Williams *et al* [18]	1998	HH, M 15-59	Khutsong	475	5.2%	LCR on urine
Auvert *et al* [98]	1999	HH, M 15-24	Khutsong	560	4.8%	LCR on urine
Williams *et al* [139]	2000	HH, M 15-49	Khutsong	606	12.4%	LCR on urine
Ndhlovu *et al* [116]	2001	HH, M 15-59	Khutsong	532	7.0%	LCR on urine
Auvert et al [151]	2002	HH, M 15-49	Orange Farm	438	6.2%	PCR on urine
Pettifor *et al* [147]	2002-3	HH, M 15-19	Semi-urban	2389	3.5%	PCR on urine
		HH, M 20-24	townships	1455	10.1%	PCR on urine
Hurkchand *et al* [148]	2002	HH, M 20-49	Mbalenhle	291	8.2%	PCR on urine
O'Leary *et al* [149]	2010	HH, M 15-19	Eastern Cape	330	8.2%	Aptima on urine

* Diagnosed by culture and a series of genetic tests, which in combination would have had very high sensitivity and specificity.

ANC = antenatal clinic attenders. CSW = commercial sex workers. DIF = direct immunofluorescence. F = females. FPC = family planning clinic attenders. HH = households. KZN = KwaZulu-Natal. LCR = ligase chain reaction test. M = males. PCR = polymerase chain reaction test. Prev. = prevalence. SDA = strand displacement amplification.

Assumptions about the sensitivity and specificity of the different assays are summarized in Table S15.

Table S15: Assumed sensitivity and specificity of different diagnostics

STI	Diagnostic	Sex	Sensitivity		Specificity		Ref
			Mean	SD	Mean	SD	
Gonorrhoea	Culture	F	0.742	0.193	0.998	0.005	[152]
	LCR on urine	M	0.921	0.029	1	0	[56, 153]
		F	0.838	0.191	1	0	[152]
	PCR on urine	M	0.904	0.029	0.997	0.007	[154]
		F	0.556	0.202	0.987	0.018	[154]
	PCR on swabs	F	0.942	0.046	0.992	0.012	[154]
	SDA on swabs	F	0.955	0.998	0.051	0.003	[155-157]
	Aptima on urine	M, F	0.886	0.998	0.138	0.001	[158-160]
Chlamydial infection	Direct immunofluorescence	F	0.763	0.02	0.988	0.008	[161-163]
	LCR on urine	M	0.875	0.121	1	0	[152]
		F	0.866	0.121	1	0	[152]
	PCR on urine	F	0.833	0.139	0.995	0.007	[154]
	PCR on swabs	F	0.855	0.116	0.996	0.005	[154]
	SDA on swabs	F	0.868	0.993	0.105	0.009	[155-157, 164]
	Aptima on urine	M, F	0.939	0.995	0.045	0.001	[158-160]

F = females. LCR = ligase chain reaction. M = males. PCR = polymerase chain reaction. SD = standard deviation. SDA = strand displacement amplification.

4. Model fits to sexually transmitted infection prevalence data

Figure S4 compares the model estimates of gonorrhoea prevalence. In women aged 15-49, the model predicts a steeper decline in gonorrhoea prevalence than suggested by the data, although there are very few recent studies, and the only two studies conducted since 2002 were conducted in sexually-active schoolgirls and pregnant women – groups known to be at a relatively high risk of gonorrhoea [165]. In sex workers the model tends to over-estimate gonorrhoea prevalence. This might be because asymptomatic gonorrhoea in sex workers often resolves due to treatment for other STIs, and this dynamic has not been modelled accurately in the model. Although many of the observations lie outside of the 95% confidence intervals, this is to be expected, as none of the observations are from nationally representative surveys, and inter-regional differences in STI prevalence, as well as variability between studies in sampling procedures and diagnostic performance, can be expected to cause substantial variation.

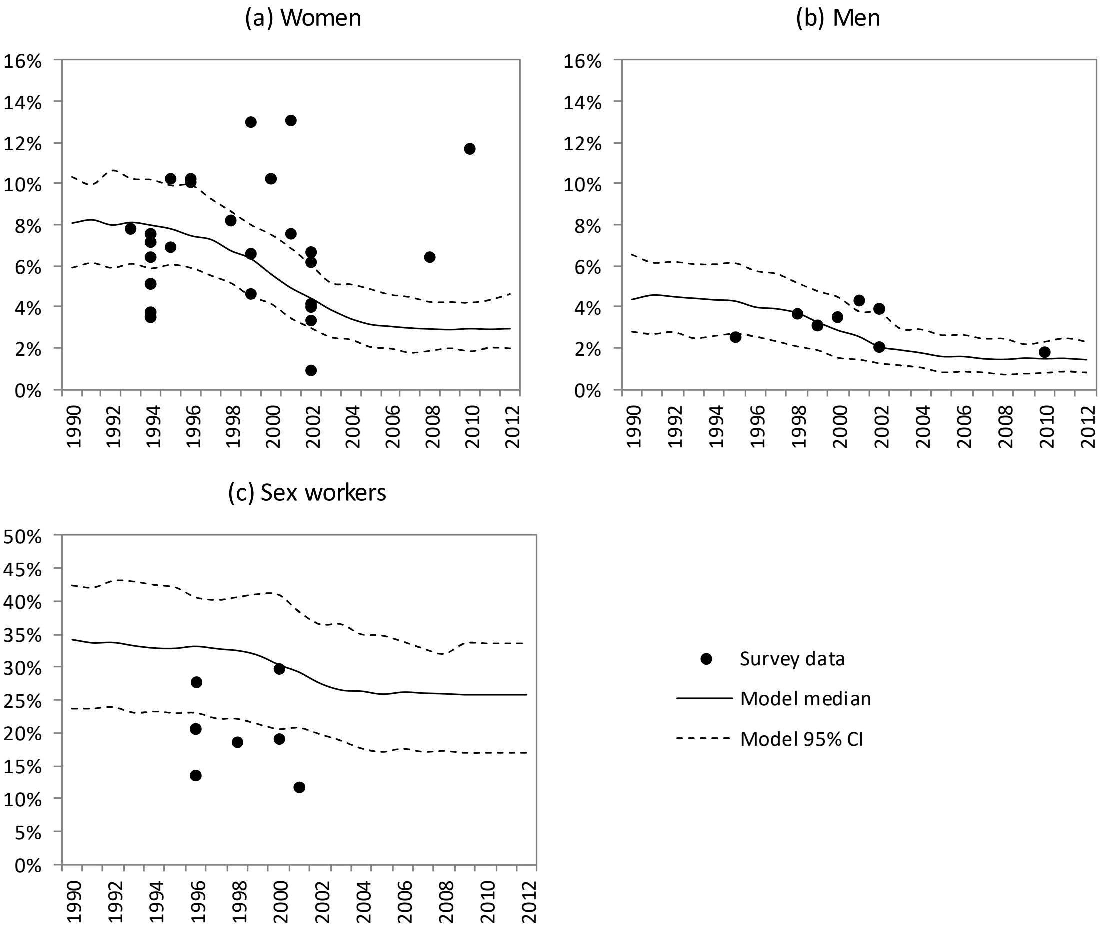

Figure S4: Prevalence of gonorrhoea

Solid lines represent the median results obtained using the 100 best-fitting parameter combinations, and dashed lines represent the 2.5 and 97.5 percentiles. Model estimates in panels (a) and (b) are for the 15-49 age group. Empirical estimates (closed circles) have been adjusted to reflect the expected sensitivities and specificities of the diagnostics used in the different studies (see Tables S13 and S15). Survey data in panel (a) include data from household surveys, antenatal surveys and surveys of women attending family planning clinics.

Figure S5 shows the estimated prevalence of chlamydia. The model is roughly consistent with the survey data, but is not consistent with the very high levels of chlamydia prevalence measured in recent surveys of women (panel a). This might be because the three most recent data points are from samples of pregnant women and sexually-active schoolgirls, and chlamydia prevalence is known to be particularly high in such samples of young women [165, 166]. Given the lack of recent data, it is difficult to argue with confidence that there has been a real increase in chlamydia prevalence over the last decade.

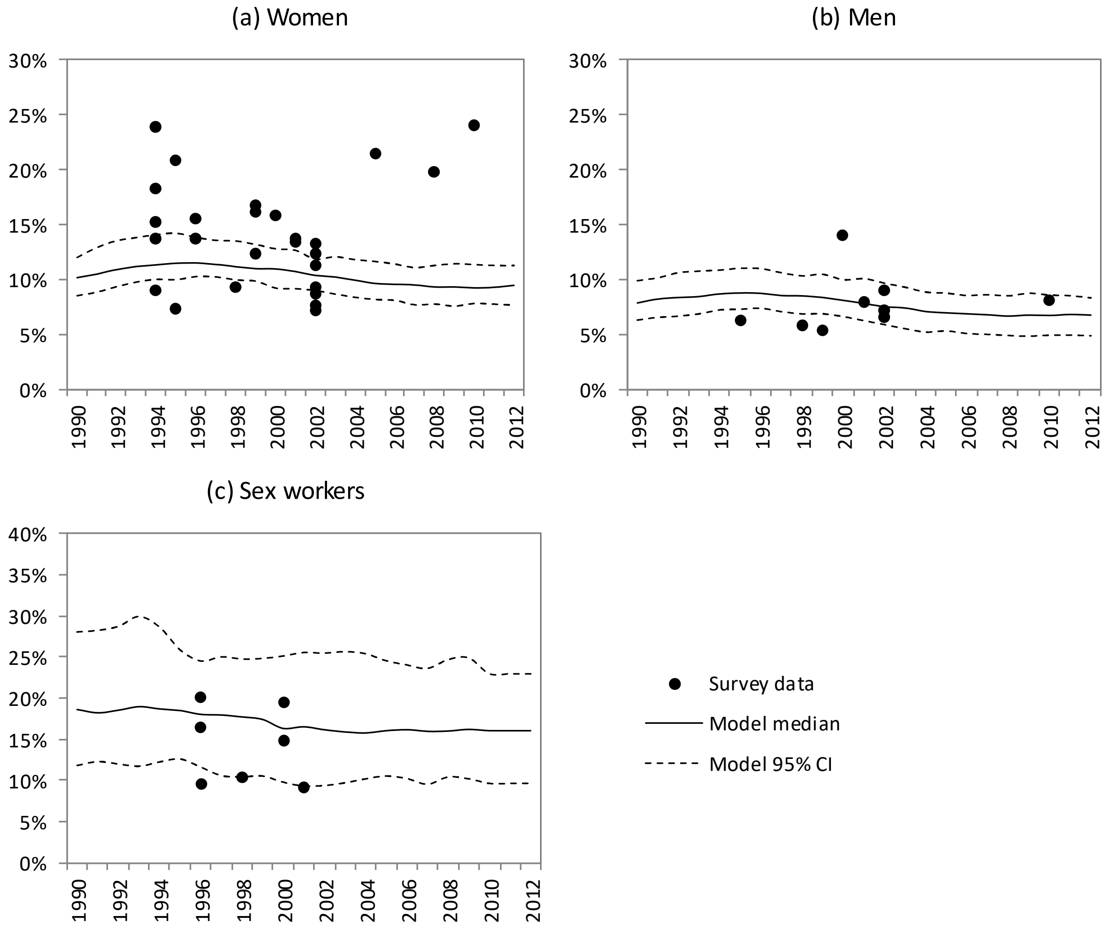

Figure S5: Prevalence of chlamydia

Solid lines represent the median results obtained using the 100 best-fitting parameter combinations. Model estimates in panels (a) and (b) are for the 15-49 age group. Empirical estimates (closed circles) have been adjusted to reflect the expected sensitivities and specificities of the diagnostics used in the different studies (see Tables S14 and S15). Survey data in panel (a) include data from household surveys, antenatal surveys and surveys of women attending family planning clinics.

Table S16 summarizes the 100 gonorrhoea parameters that gave the best fits to the gonorrhoea prevalence data. The model tended to give a better fit to the data when the average duration of untreated infection was longer than that assumed *a priori*.

Table S16: Comparison of prior distributions and best-fitting parameter combinations: gonorrhoea

Parameter	Prior distribution (median, IQR)	Best-fitting parameters (median, IQR)
Transmission probability per sex act		
M-to-F	39.7% (32.9-46.8%)	45.9% (40.1-53.1%)
F-to-M	19.7% (16.4-23.2%)	23.7% (19.8-27.2%)
% of cases that become symptomatic		
Male	90.8% (87.1-93.7%)	86.6% (83.5-89.6%)
Female	39.3% (28.9-50.4%)	29.9% (22.6-39.2%)
Average duration (in weeks)		
Male infection (untreated)	18.4 (12.7-25.5)	34.0 (25.8-38.3)
Female infection (untreated)	18.4 (12.7-25.5)	33.6 (29.5-38.9)
Immunity	47.7 (33.0-66.4)	48.8 (41.8-61.8)
% immune after treatment cure	50.0% (25.0-75.0%)	40.1% (20.4-59.0%)
% symptoms correctly treated pre-SM	70.7% (63.4-77.3%)	70.4% (65.2-76.2%)

F-to-M = female-to-male; IQR = inter-quartile range; M-to-F = male-to-female; SM = syndromic management.

Table S17 shows the correlation between the 100 best-fitting parameter combinations. Several of the parameters are strongly correlated, reflecting identifiability issues. For example, there is a strong negative correlation (-0.48) between the average duration of immunity and the fraction of patients who experience immunity following treatment of gonorrhoea. This suggests that there is a particular prevalence of anti-gonorrhoea immunity that yields an optimal model fit to the gonorrhoea prevalence data, and that increases in the average duration of immunity would need to be offset by decreases in the proportion of treated individuals who develop immunity in order for this optimal prevalence to be maintained.

Table S17: Correlations coefficients between the 100 best-fitting parameter combinations: gonorrhoea

	M-to-F transmission	F-to-M transmission	Male symptomatic %	Female symptomatic %	Male duration	Female duration	% immune post-treatment	Immunity duration	Correctly treated %
M-to-F transmission	1.00	-0.06	-0.01	-0.15	-0.10	**-0.31**	-0.06	-0.02	**0.28**
F-to-M transmission		1.00	**0.33**	0.07	**-0.37**	0.00	0.07	0.04	-0.02
Male symptomatic %			1.00	0.01	0.06	0.07	**-0.27**	0.10	0.01
Female symptomatic %				1.00	0.00	**0.33**	-0.20	0.08	0.09
Male duration					1.00	-0.20	0.12	-0.04	0.08
Female duration						1.00	0.11	**0.43**	-0.14
% immune post-treatment							1.00	**-0.48**	0.02
Immunity duration								1.00	-0.18
Correctly treated %									1.00

F-to-M = female-to-male; IQR = inter-quartile range; M-to-F = male-to-female. Highlighted values are statistically significant at the 5% significance level.

Table S18 summarizes the 100 chlamydia parameters that gave the best fits to the chlamydia prevalence data. The best-fitting immunity parameters differed substantially from the values assumed to be most plausible *a priori*: the model fitted the chlamydia prevalence data better with a shorter average duration of immunity but a greater fraction of patients experiencing immunity following treatment.

Table S18: Comparison of prior distributions and best-fitting parameter combinations: chlamydia

Parameter	Prior distribution (median, IQR)	Best-fitting parameters (median, IQR)
Transmission probability per sex act		
M-to-F	11.1% (7.5-15.5%)	16.2% (11.8-20.5%)
F-to-M	14.2% (8.4-21.8%)	9.75% (7.13-15.4%)
% of cases that become symptomatic		
Male	28.3% (18.5-39.8%)	36.7% (26.5-45.0%)
Female	13.8% (9.0-19.7%)	11.6% (7.0-15.9%)
Average duration (in weeks)		
Symptomatic infection (untreated)	14.7 (10.1-20.4)	15.0 (10.2-19.5)
Asymptomatic infection (untreated)	89.2 (79.5-99.6)	106.6 (96.7-116.6)
Immunity	495 (375-637)	295 (241-343)
% immune after treatment cure	50.0% (25.0-75.0%)	73.2% (59.5-88.4%)
% symptoms correctly treated pre-SM	70.7% (63.4-77.3%)	71.1% (63.0-78.2%)

F-to-M = female-to-male; IQR = inter-quartile range; M-to-F = male-to-female; SM = syndromic management.

Table S19 shows the correlation between the 100 best-fitting parameter combinations. As with gonorrhoea, several of the parameters are strongly correlated. For example, the strong positive correlation between the female-to-male transmission probability and the male symptomatic proportion (r = 0.62) suggests that an increase in the male symptomatic fraction is associated with a reduction in the prevalence of chlamydia in men (since symptomatic infections tend to have a much shorter average duration than asymptomatic infections), and that this decrease will need to be offset by an increase in the female-to-male transmission probability if the model estimates of chlamydia prevalence in men are to remain consistent with observations.

Table S19: Correlations coefficients between the 100 best-fitting parameter combinations: chlamydia

	M-to-F transmission	F-to-M transmission	Male symptomatic %	Female symptomatic %	Male duration	Female duration	% immune post-treatment	Immunity duration	Correctly treated %
M-to-F transmission	1.00	**-0.20**	-0.14	0.15	0.09	-0.14	-0.16	**0.23**	-0.14
F-to-M transmission		1.00	**0.62**	-0.10	0.01	-0.06	0.20	-0.13	0.02
Male symptomatic %			1.00	0.02	-0.04	**0.31**	0.15	-0.08	0.06
Female symptomatic %				1.00	-0.07	**0.22**	-0.06	-0.11	-0.05
Male duration					1.00	**-0.21**	-0.10	-0.12	0.13
Female duration						1.00	0.08	**0.61**	-0.02
% immune post-treatment							1.00	**-0.23**	-0.06
Immunity duration								1.00	-0.05
Correctly treated %									1.00

F-to-M = female-to-male; IQR = inter-quartile range; M-to-F = male-to-female. Highlighted values are statistically significant at the 5% significance level.

References

1. Johnson LF, Geffen N. A comparison of two mathematical modeling frameworks for evaluating sexually transmitted infection epidemiology. *Sexually Transmitted Diseases* 2016; **43**:139-146.
2. Budlender D, Chobokoane N, Simelane S. Marriage patterns in South Africa: Methodological and substantive issues. *Southern African Journal of Demography* 2004; **9**:1-26.
3. Dunkle KL, Jewkes RK, Brown HC, Gray GE, McIntryre JA, Harlow SD. Transactional sex among women in Soweto, South Africa: prevalence, risk factors and association with HIV infection. *Social Science and Medicine* 2004; **59**:1581-1592.
4. Jewkes RK, Nduna M, Jama PN, Dunkle KL, Levin JB. Steadys, roll-ons and hit and runs: using indigenous typology to measure number of sexual partners [Abstract TuPpE2069]. *14th International AIDS Conference*. Barcelona, Spain; 2002.
5. Shisana O, Rehle T, Simbayi LC, Parker W, Zuma K, Bhana A*, et al.* South African National HIV Prevalence, HIV Incidence, Behaviours and Communication Survey, 2005. Cape Town: HSRC Press; 2005. Available: http://www.hsrcpress.ac.za. Accessed 1 Dec 2005
6. Department of Health. National HIV and Syphilis Prevalence Survey, South Africa 2005. Pretoria: Directorate of Epidemiology and Surveillance; 2006. Available: http://www.doh.gov.za/docs/reports-f.html. Accessed 6 Aug 2010
7. Johnson LF, Dorrington RE, Bradshaw D, Pillay-Van Wyk V, Rehle TM. Sexual behaviour patterns in South Africa and their association with the spread of HIV: insights from a mathematical model. *Demographic Research* 2009; **21**:289-340.
8. Jewkes R, Vundule C, Maforah F, Jordaan E. Relationship dynamics and teenage pregnancy in South Africa. *Social Science and Medicine* 2001; **52**:733-744.
9. Ross A, Van der Paal L, Lubega R, Mayanja BN, Shafer LA, Whitworth J. HIV-1 disease progression and fertility: the incidence of recognized pregnancy and pregnancy outcome in Uganda. *AIDS* 2004; **18**:799-804.
10. Terceira N, Gregson S, Zaba B, Mason PR. The contribution of HIV to fertility decline in rural Zimbabwe, 1985-2000. *Population Studies* 2003; **57**:149-164.
11. Hankins C, Tran T, Lapointe N. Sexual behaviour and pregnancy outcome in HIV-infected women. *Journal of Acquired Immune Deficiency Syndromes* 1998; **18**:479-487.
12. Greenblatt RM, Bacchetti P, Barkan S, Augenbraun M, Silver S, Delapenha R*, et al.* Lower genital tract infections among HIV-infected and high-risk uninfected women: findings of the Women's Interagency HIV Study (WIHS). *Sexually Transmitted Diseases* 1999; **26**:143-151.
13. Moatti JP, Prudhomme J, Traore DC, Juillet-Amari A, Akribi HA, Msellati P. Access to antiretroviral treatment and sexual behaviours of HIV-infected patients aware of their serostatus in Côte d'Ivoire. *AIDS* 2003; **17 (Suppl 3)**:S69-77.
14. Bunnell R, Ekwaru JP, Solberg P, Wamai N, Bikaako-Kajura W, Were W*, et al.* Changes in sexual behavior and risk of HIV transmission after antiretroviral therapy and prevention interventions in rural Uganda. *AIDS* 2006; **20**:85-92.
15. Mpofu E, Flisher AJ, Bility K, Onya H, Lombard C. Sexual partners in a rural South African setting. *AIDS and Behavior* 2006; **10**:399-404.

16. Pettifor AE, van der Straten A, Dunbar MS, Shiboski SC, Padian NS. Early age of first sex: a risk factor for HIV infection among women in Zimbabwe. *AIDS* 2004; **18**:1435-1442.
17. Department of Health. South Africa Demographic and Health Survey 1998: Full Report. 1999.
18. Williams B, Gilgen D, Campbell C, Taljaard D, MacPhail C. *The natural history of HIV / AIDS in South Africa: A biomedical and social survey in Carletonville*. Johannesburg: Council for Scientific and Industrial Research; 2000.
19. Hallman K. Socioeconomic Disadvantage and Unsafe Sexual Behaviors among Young Women and Men in South Africa. New York: Population Council, Policy Research Division; 2004.
20. Kelly K. Communicating for action: A contextual evaluation of youth responses to HIV/AIDS. Department of Health; 2000. Available: http://www.cadre.org.za. Accessed 12 October 2006
21. Nnko S, Boerma JT, Urassa M, Mwaluko G, Zaba B. Secretive females or swaggering males? An assessment of the quality of sexual partnership reporting in rural Tanzania. *Social Science and Medicine* 2004; **59**:299-310.
22. Ferry B, Caraël M, Buvé A, Auvert B, Laourou M, Kanhonou L*, et al.* Comparison of key parameters of sexual behaviour in four African urban populations with different levels of HIV infection. *AIDS* 2001; **15**:S41-50.
23. Statistics South Africa. Marriages and divorces, 2004. Pretoria; 2006. Available: http://www.statssa.gov.za/publications/Report-03-07-01/Report-03-07-012004.pdf. Accessed 2 Aug 2008
24. Konstant TL, Rangasami J, Stacey MJ, Stewart ML, Nogoduka C. Estimating the number of sex workers in South Africa: rapid population size estimation. *AIDS and Behavior* 2015; **19 (Suppl 1)**:S3-15.
25. Varga CA. The condom conundrum: barriers to condom use among commercial sex workers in Durban, South Africa. *African Journal of Reproductive Health* 1997; **1**:74-88.
26. Abdool Karim QA, Abdool Karim SS, Soldan K, Zondi M. Reducing the risk of HIV infection among South African sex workers: socioeconomic and gender barriers. *American Journal of Public Health* 1995; **85**:1521-1525.
27. Ramjee G, Abdool Karim SS, Sturm AW. Sexually transmitted infections among sex workers in KwaZulu-Natal, South Africa. *Sexually Transmitted Diseases* 1998; **25**:346-349.
28. Dunkle KL, Beksinska ME, Rees VH, Ballard RC, Htun Y, Wilson ML. Risk factors for HIV infection among sex workers in Johannesburg, South Africa. *International Journal of STD and AIDS* 2005; **16**:256-261.
29. Gould C, Fick N. *Selling Sex in Cape Town: Sex Work and Human Trafficking in a South African City*: Institute for Security Studies; 2008.
30. Peltzer K, Seoka P, Raphala S. Characteristics of female sex workers and their HIV/AIDS/STI knowledge, attitudes and behaviour in semi-urban areas in South Africa. *Curationis* 2004; **March 2004**:4-11.
31. van Loggerenberg F, Mlisana K, Williamson C, Auld SC, Morris L, Gray CM*, et al.* Establishing a cohort at high risk of HIV infection in South Africa: challenges and experiences of the CAPRISA 002 acute infection study. *PLoS One* 2008; **3**:e1954.
32. Delva W, Richter M, De Koker P, Chersich M, Temmerman M. Sex work during the 2010 FIFA World Cup: results from a three-wave cross-sectional survey. *PLoS One* 2011; **6**:e28363.

33. Caraël M, Slaymaker E, Lyerla R, Sarkar S. Clients of sex workers in different regions of the world: hard to count. *Sexually Transmitted Infections* 2006; **82 (Suppl 3)**:iii26-33.
34. Rees H, Beksinska ME, Dickson-Tetteh K, Ballard RC, Htun Y. Commercial sex workers in Johannesburg: risk behaviour and HIV status. *South African Journal of Science* 2000; **96**:283-284.
35. Wingood GM, Reddy P, Lang DL, Saleh-Onoya D, Braxton N, Sifunda S*, et al.* Efficacy of SISTA South Africa on sexual behavior and relationship control among isiXhosa women in South Africa: results of a randomized-controlled trial. *Journal of Acquired Immune Deficiency Syndromes* 2013; **63 (Suppl 1)**:S59-65.
36. Harling G, Newell ML, Tanser F, Kawachi I, Subramanian S, Bärnighausen T. Do age-disparate relationships drive HIV incidence in young women? Evidence from a population cohort in rural KwaZulu-Natal, South Africa. *Journal of Acquired Immune Deficiency Syndromes* 2014; **66**:443-451.
37. Johnson S, Kincaid DL, Figueroa ME, Delate R, Mahlasela L, Magni S. The Third National HIV Communication Survey, 2012. Pretoria: Johns Hopkins Health and Education in South Africa; 2013. Available: http://jhhesa.org/sites/default/files/hiv_survey.pdf. Accessed 19 April 2014
38. Katz I, Low-Beer D. Why has HIV stabilized in South Africa, yet not declined further? Age and sexual behavior patterns among youth. *Sexually Transmitted Diseases* 2008; **35**:837-842.
39. Johnson LF, Hallett TB, Rehle TM, Dorrington RE. The effect of changes in condom usage and antiretroviral treatment coverage on HIV incidence in South Africa: a model-based analysis. *Journal of the Royal Society Interface* 2012; **9**:1544-1554.
40. Department of Health. South Africa Demographic and Health Survey 2003: Preliminary Report. Pretoria; 2004. Available: http://www.doh.gov.za/docs/reports/2003/sadhs2003/part2.pdf. Accessed 6 Jan 2012
41. Kaufman CE. The politics and practice of reproductive control in South Africa: a multilevel analysis of fertility and contraceptive use [Doctoral thesis]. Ann Arbor: University of Michigan; 1996.
42. Jochelson K, Mothibeli M, Leger JP. Human immunodeficiency virus and migrant labor in South Africa. *International Journal of Health Services* 1991; **21**:157-173.
43. Richter ML, Chersich M, Temmerman M, Luchters S. Characteristics, sexual behaviour and risk factors of female, male and transgender sex workers in South Africa. *South African Medical Journal* 2013; **103**:246-251.
44. Sex Worker Education and Advocacy Taskforce. Beginning to build the picture: South African national survey of sex worker knowledge, experiences and behaviour. 2013.
45. Human Sciences Research Council. South African national HIV prevalence, behavioural risks and mass media household survey 2002. 2002. Available: http://www.hsrcpress.ac.za. Accessed 18 Feb 2009
46. Reproductive Health Research Unit. HIV and sexual behaviour among young South Africans: a national survey of 15-24 year olds. Joint publication of Reproductive Health Research Unit and loveLife; 2004. Available: www.rhru.co.za/images/Docs/national%20survey%20RHRU.pdf. Accessed 8 May 2004
47. Shisana O, Rehle T, Simbayi LC, Zuma K, Jooste S, Pillay-van Wyk V*, et al.* South African national HIV prevalence, incidence, behaviour and communication survey,

2008: A turning tide among teenagers? Cape Town: Human Sciences Research Council; 2009. Available: http://www.hsrcpress.ac.za. Accessed 9 June 2009
48. Boily MC, Asghar Z, Garske T, Ghani AC, Poulin R. Influence of selected formation rules for for finite population networks with fixed macrostructures: implications for individual-based model of infectious diseases. *Mathematical Population Studies* 2007; **14**:237-267.
49. Plummer FA, Simonsen JN, Chubb H, Slaney L, Kimata J, Bosire M*, et al.* Epidemiologic evidence for the development of serovar-specific immunity after gonococcal infection. *Journal of Clinical Investigation* 1989; **83**:1472-1476.
50. Moodley P, Martin IM, Ison CA, Sturm AW. Typing of *Neisseria gonorrhoeae* reveals rapid reinfection in rural South Africa. *Journal of Clinical Microbiology* 2002; **40**:4567-4570.
51. Schmidt KA, Schneider H, Lindstrom JA, Boslego JW, Warren RA, Van de Verg L*, et al.* Experimental gonococcal urethritis and reinfection with homologous gonococci in male volunteers. *Sexually Transmitted Diseases* 2001; **28**:555-564.
52. Johnson LF, Alkema L, Dorrington RE. A Bayesian approach to uncertainty analysis of sexually transmitted infection models. *Sexually Transmitted Infections* 2010; **86**:169-174.
53. Johnson LF, Dorrington RE, Bradshaw D. The role of immunity in the epidemiology of gonorrhoea, chlamydial infection and trichomoniasis: insights from a mathematical model. *Epidemiology and Infection* 2011; **139**:1875-1883.
54. Garnett GP, Mertz KJ, Finelli L, Levine WC, St Louis ME. The transmission dynamics of gonorrhoea: modelling the reported behaviour of infected patients from Newark, New Jersey. *Philosophical Transactions of the Royal Society of London. Series B* 1999; **354**:787-797.
55. Harrison WO, Hooper RR, Wiesner PJ, Campbell AF, Karney WW, Reynolds GH*, et al.* A trial of minocycline given after exposure to prevent gonorrhea. *New England Journal of Medicine* 1979; **300**:1074-1078.
56. Buimer M, van Doornum GJ, Ching S, Peerbooms PG, Plier PK, Ram D*, et al.* Detection of *Chlamydia trachomatis* and *Neisseria gonorrhoeae* by ligase chain reaction-based assays with clinical specimens from various sites: implications for diagnostic testing and screening. *Journal of Clinical Microbiology* 1996; **34**:2395-2400.
57. Price MA, Miller WC, Kaydos-Daniels SC, Hoffman IF, Chilongozi D, Martinson FE*, et al.* Trichomoniasis in men and HIV infection: data from 2 outpatient clinics at Lilongwe Central Hospital, Malawi. *Journal of Infectious Diseases* 2004; **190**:1448-1455.
58. Joyner JL, Douglas JM, Jr., Ragsdale S, Foster M, Judson FN. Comparative prevalence of infection with *Trichomonas vaginalis* among men attending a sexually transmitted diseases clinic. *Sexually Transmitted Diseases* 2000; **27**:236-240.
59. Hobbs MM, Kazembe P, Reed AW, Miller WC, Nkata E, Zimba D*, et al.* *Trichomonas vaginalis* as a cause of urethritis in Malawian men. *Sexually Transmitted Diseases* 1999; **26**:381-387.
60. Platt R, Rice PA, McCormack WM. Risk of acquiring gonorrhea and prevalence of abnormal adnexal findings among women recently exposed to gonorrhea. *Journal of the American Medical Association* 1983; **250**:3205-3209.
61. Korenromp EL, Sudaryo MK, de Vlas SJ, Gray RH, Sewankambo NK, Serwadda D*, et al.* What proportion of episodes of gonorrhoea and chlamydia becomes symptomatic? *International Journal of STD and AIDS* 2002; **13**:91-101.

62. Handsfield HH, Lipman TO, Harnisch JP, Tronca E, Holmes KK. Asymptomatic gonorrhea in men. Diagnosis, natural course, prevalence and significance. *New England Journal of Medicine* 1974; **290**:117-123.
63. Lin JS, Donegan SP, Heeren TC, Greenberg M, Flaherty EE, Haivanis R*, et al.* Transmission of *Chlamydia trachomatis* and *Neisseria gonorrhoeae* among men with urethritis and their female sex partners. *Journal of Infectious Diseases* 1998; **178**:1707-1712.
64. Orroth KK, Freeman EE, Bakker R, Buvé A, Glynn JR, Boily M*, et al.* Understanding differences between contrasting HIV epidemics in East and West Africa: results from a simulation model of the Four Cities Study. *Sexually Transmitted Infections* 2007; **83 (Suppl 1)**:i5-16.
65. Bracher M, Santow G, Watkins SC. 'Moving' and marrying: modelling HIV infection among newly-weds in Malawi. *Demographic Research* 2003; **Special Collection 1**:207-246.
66. Korenromp EL, Van Vliet C, Grosskurth H, Gavyole A, Van der Ploeg CPB, Fransen L*, et al.* Model-based evaluation of single-round mass treatment of sexually transmitted diseases for HIV control in a rural African population. *AIDS* 2000; **14**:573-593.
67. Ghani AC, Aral SO. Patterns of sex worker-client contacts and their implications for the persistence of sexually transmitted infections. *Journal of Infectious Diseases* 2005; **191 Suppl 1**:S34-41.
68. Holmes KK, Johnson DW, Trostle HJ. An estimate of the risk of men acquiring gonorrhea by sexual contact with infected females. *American Journal of Epidemiology* 1970; **91**:170-174.
69. Hooper RR, Reynolds GH, Jones OG, Zaidi A, Wiesner PJ, Latimer KP*, et al.* Cohort study of veneral disease. I: The risk of gonorrhea transmission from infected women to men. *American Journal of Epidemiology* 1978; **108**:136-144.
70. Mathews C, van Rensburg A, Schierhout G, Coetzee N, Lombard CJ, Fehler HG*, et al.* An assessment of care provided by a public sector STD clinic in Cape Town. *International Journal of STD and AIDS* 1998; **9**:689-694.
71. Somsé P, Mberyo-Yaah F, Morency P, Dubois MJ, Grésenguet G, Pépin J. Quality of sexually transmitted disease treatments in the formal and informal sectors of Bangui, Central African Republic. *Sexually Transmitted Diseases* 2000; **27**:458-464.
72. Buvé A, Changalucha J, Mayaud P, Gavyole A, Mugeye K, Todd J*, et al.* How many patients with a sexually transmitted infection are cured by health services? A study from Mwanza region, Tanzania. *Tropical Medicine and International Health* 2001; **6**:971-979.
73. Chabikuli N, Schneider H, Blaauw D, Zwi AB, Brugha R. Quality and equity of private sector care for sexually transmitted diseases in South Africa. *Health Policy and Planning* 2002; **17**:40-46.
74. Brunham RC, Kimani J, Bwayo J, Maitha G, Maclean I, Yang C*, et al.* The epidemiology of *Chlamydia trachomatis* within a sexually transmitted diseases core group. *Journal of Infectious Diseases* 1996; **173**:950-956.
75. Arno JN, Katz BP, McBride R, Carty GA, Batteiger BE, Caine VA*, et al.* Age and clinical immunity to infections with *Chlamydia trachomatis*. *Sexually Transmitted Diseases* 1994; **21**:47-52.
76. Golden MR, Schillinger JA, Markowitz L, St Louis ME. Duration of untreated genital infections with chlamydia trachomatis: a review of the literature. *Sexually Transmitted Diseases* 2000; **27**:329-337.

77. Schachter J, Cles LD, Ray RM, Hesse FE. Is there immunity to chlamydial infections of the human genital tract? *Sexually Transmitted Diseases* 1983; **10**:123-125.
78. Katz BP, Batteiger BE, Jones RB. Effect of prior sexually transmitted disease on the isolation of *Chlamydia trachomatis*. *Sexually Transmitted Diseases* 1987; **14**:160-164.
79. Brunham RC, Pourbohloul B, Mak S, White R, Rekart ML. The unexpected impact of a *Chlamydia trachomatis* infection control program on susceptibility to reinfection. *Journal of Infectious Diseases* 2005; **192**:1836-1844.
80. Rekart ML, Brunham RC. Epidemiology of chlamydial infection: are we losing ground? *Sexually Transmitted Infections* 2008; **84**:87-91.
81. Su H, Morrison R, Messer R, Whitmire W, Hughes S, Caldwell HD. The effect of doxycycline treatment on the development of protective immunity in a murine model of chlamydial genital infection. *Journal of Infectious Diseases* 1999; **180**:1252-1258.
82. Krieger JN, Jenny C, Verdon M, Siegel N, Springwater R, Critchlow CW*, et al.* Clinical manifestations of trichomoniasis in men. *Annals of Internal Medicine* 1993; **118**:844-849.
83. Parks KS, Dixon PB, Richey CM, Hook EWI. Spontaneous clearance of *Chlamydia trachomatis* infection in untreated patients. *Sexually Transmitted Diseases* 1997; **24**:229-235.
84. Rahm VA, Gnarpe H, Odlind V. *Chlamydia trachomatis* among sexually active teenage girls. Lack of correlation between chlamydial infection, history of the patient and clinical signs of infection. *British Journal of Obstetrics and Gynaecology* 1988; **95**:916-919.
85. Molano M, Meijer CJ, Weiderpass E, Arslan A, Posso H, Franceschi S*, et al.* The natural course of *Chlamydia trachomatis* infection in asymptomatic Colombian women: a 5-year follow-up study. *Journal of Infectious Diseases* 2005; **191**:907-916.
86. Morré SA, van den Brule AJ, Rozendaal L, Boeke AJ, Voorhorst FJ, de Blok S*, et al.* The natural course of asymptomatic *Chlamydia trachomatis* infections: 45% clearance and no development of clinical PID after one-year follow-up. *International Journal of STD and AIDS* 2002; **13 (Suppl 2)**:12-18.
87. Wawer MJ, Gray RH, Sewankambo NK, Serwadda D, Li X, Laeyendecker O*, et al.* Rates of HIV-1 transmission per coital act, by stage of HIV-1 infection, in Rakai, Uganda. *Journal of Infectious Diseases* 2005; **191**:1403-1409.
88. Johnson LF, Dorrington RE. Modelling the demographic impact of HIV/AIDS in South Africa and the likely impact of interventions. *Demographic Research* 2006; **14**:541-574.
89. Johnson LF, Dorrington RE, Matthews AP. An investigation into the extent of uncertainty surrounding estimates of the impact of HIV/AIDS in South Africa. *South African Journal of Science* 2007; **103**:135-140.
90. Johnson LF, Mossong J, Dorrington RE, Schomaker M, Hoffmann CJ, Keiser O*, et al.* Life expectancies of South African adults starting antiretroviral treatment: Collaborative analysis of cohort studies. *PLoS Medicine* 2013; **10**:e1001418.
91. Hollingsworth TD, Anderson RM, Fraser C. HIV-1 transmission, by stage of infection. *Journal of Infectious Diseases* 2008; **198**:687-693.
92. Cohen MS, Dye C, Fraser C, Miller WC, Powers KA, Williams BG. HIV treatment as prevention: debate and commentary - will early infection compromise treatment-as-prevention strategies? *PLoS Medicine* 2012; **9**:e1001232.

93. Boily MC, Baggaley RF, Wang L, Masse B, White RG, Hayes RJ*, et al.* Heterosexual risk of HIV-1 infection per sexual act: systematic review and meta-analysis of observational studies. *Lancet Infectious Diseases* 2009; **9**:118-129.
94. Ramjee G, Williams B, Gouws E, Van Dyck E, De Deken B, Abdool Karim S. The impact of incident and prevalent herpes simplex virus-2 infection on the incidence of HIV-1 infection among commercial sex workers in South Africa. *Journal of Acquired Immune Deficiency Syndromes* 2005; **39**:333-339.
95. Ramjee G, Gouws E. Prevalence of HIV among truck drivers visiting sex workers in KwaZulu-Natal, South Africa. *Sexually Transmitted Diseases* 2002; **29**:44-49.
96. Ramjee G, Weber AE, Morar NS. Recording sexual behavior: comparison of recall questionnaires with a coital diary. *Sexually Transmitted Diseases* 1999; **26**:374-380.
97. Hayes RJ, Schulz KF, Plummer FA. The cofactor effect of genital ulcers on the per-exposure risk of HIV transmission in sub-Saharan Africa. *Journal of Tropical Medicine and Hygiene* 1995; **98**:1-8.
98. Auvert B, Ballard R, Campbell C, Caraël M, Carton M, Fehler G*, et al.* HIV infection in a South African mining town is associated with herpes simplex virus-2 seropositivity and sexual behaviour. *AIDS* 2001; **15**:885-898.
99. Pettifor AE, Hudgens MG, Levandowski BA, Rees HV, Cohen MS. Highly efficient HIV transmission to young women in South Africa. *AIDS* 2007; **21**:861-865.
100. Mahiane SG, Legeai C, Taljaard D, Latouche A, Puren A, Peillon A*, et al.* Transmission probabilities of HIV and herpes simplex virus type 2, effect of male circumcision and interaction: a longitudinal study in a township of South Africa. *AIDS* 2009; **23**:377-383.
101. Baeten JM, Richardson BA, Lavreys L, Rakwar JP, Mandaliya K, Bwayo JJ*, et al.* Female-to-male infectivity of HIV-1 among circumcised and uncircumcised Kenyan men. *Journal of Infectious Diseases* 2005; **191**:546-553.
102. Gray R, Wawer M, Brookmeyer R, Sewankambo N, Serwadda D, Wabwire-Mangen F*, et al.* Probability of HIV-1 transmission per coital act in monogamous, heterosexual, HIV-1-discordant couples in Rakai, Uganda. *Lancet* 2001; **357**:1149-1153.
103. Allen S, Tice J, Van de Perre P, Serufilira A, Hudes E, Nsengumuremyi F*, et al.* Effect of serotesting with counselling on condom use and seroconversion among HIV discordant couples in Africa. *British Medical Journal* 1992; **304**:1605-1609.
104. Hughes JP, Baeten JM, Lingappa JR, Magaret AS, Wald A, de Bruyn G*, et al.* Determinants of per-coital-act HIV-1 infectivity among African HIV-1-serodiscordant couples. *Journal of Infectious Diseases* 2012; **205**:358-365.
105. Department of National Health and Population Development. First national HIV survey of women attending antenatal clinics, South Africa, October/November 1990. *Epidemiological comments.* 1991; **18**:35-45.
106. Montana LS, Mishra V, Hong R. Comparison of HIV prevalence estimates from antenatal care surveillance and population-based surveys in sub-Saharan Africa. *Sexually Transmitted Infections* 2008; **84 (Suppl 1)**:i78-i84.
107. Williams BG, Gouws E, Colvin M, Sitas F, Ramjee G, Abdool Karim SS. Patterns of infection: using age prevalence data to understand the epidemic of HIV in South Africa. *South African Journal of Science* 2000; **96**:305-312.
108. Johnson LF, Dorrington RE, Bradshaw D, Coetzee DJ. The effect of syndromic management interventions on the prevalence of sexually transmitted infections in South Africa. *Sexual and Reproductive Healthcare* 2011; **2**:13-20.

109. Wilkinson D, Connolly A, Harrison A, Lurie M, Abdool Karim SS. Sexually transmitted disease syndromes in rural South Africa: results from health facility surveillance. *Sexually Transmitted Diseases* 1998; **25**:20-23.
110. O'Farrell N, Hoosen AA, Coetzee KD, Van den Ende J. Genital ulcer disease in men in Durban, South Africa. *Genitourinary Medicine* 1991; **67**:327-330.
111. O'Farrell N, Hoosen AA, Coetzee KD, Van den Ende J. Genital ulcer disease in women in Durban, South Africa. *Genitourinary Medicine* 1991; **67**:322-326.
112. Reddy SP, Panday S, Swart D, Jinabhai CC, Amosun SL, James S*, et al.* Umthenthe Uhlaba Usamila - The South African Youth Risk Behaviour Survey 2002. Cape Town: South African Medical Research Council; 2003. Available: http://www.mrc.ac.za/healthpromotion/healthpromotion.htm. Accessed 15 Dec 2003
113. Lewis JJ, Garnett GP, Nyamukapa CA, Donnelly CA, Mason PR, Gregson S. Patterns of uptake of treatment for self reported sexually transmitted infection symptoms in rural Zimbabwe. *Sexually Transmitted Infections* 2005; **81**:326-332.
114. Wilkinson D, Wilkinson N. HIV infection among patients with sexually transmitted diseases in rural South Africa. *International Journal of STD and AIDS* 1998; **9**:736-739.
115. Wilson T, Strebel A, Simbayi L, Andipatin M, Potgieter C, Ratele K*, et al.* Health care-seeking behaviour for sexually transmitted diseases in South Africa. *Journal of Psychology in Africa* 2000; **10**:101-121.
116. Ndhlovu L, Searle C, Van Dam J, Mzaidume Y, Rasego B, Moema S. Reducing the transmission of HIV and sexually transmitted infections in a mining community: Findings from the Carletonville Mothusimpilo intervention project: 1998 to 2001. Washington, DC: Population Council; 2005. Available: http://www.popcouncil.org/pdfs/horizons/crltnvll.pdf. Accessed 1 Dec 2006
117. Eaton JW, Johnson LF, Salomon JA, Bärnighausen T, Bendavid E, Bershteyn A*, et al.* HIV treatment as prevention: systematic comparison of mathematical models of the potential impact of antiretroviral therapy on HIV incidence in South Africa. *PLoS Medicine* 2012; **9**:e1001245.
118. Lewis DA, Scott L, Slabbert M, Mkhonto S, van Zijl A, Ntuli D*, et al.* Escalation in the prevalence of ciprofloxacin resistant gonorrhoea in men presenting with urethritis to public health facilities in two South African cities [Abstract P-449]. *17th International Society for STD Research World Congress*. Seattle, USA; 2007.
119. Black V, Magooa P, Radebe F, Myers M, Pillay C, Lewis DA. The detection of urethritis pathogens among patients with the male urethritis syndrome, genital ulcer syndrome and HIV VCT clients: should South Africa's syndromic management approach be revised? *Sexually Transmitted Infections* 2008; **84**:254-258.
120. Moodley P, Sturm AW. Ciprofloxacin-resistant gonorrhoea in South Africa. *Lancet* 2005; **366**:1159.
121. Department of Health. First Line Comprehensive Management and Control of Sexually Transmitted Infections (STIs). Pretoria; 2009. Available: http://www.nicd.ac.za/units/stirc. Accessed 10 July 2009
122. Harrison A, Wilkinson D, Lurie M, Connolly AM, Abdool Karim S. Improving quality of sexually transmitted disease case management in rural South Africa. *AIDS* 1998; **12**:2329-2335.
123. Dartnall E, Schneider H, Hlatswayo Z, Clews F. STD management in the private sector: a national evaluation. Centre for Health Policy; 1997.
124. Pick W, Fisher B, Kowo H, Conway S, Kgosidintsi N, Weiner R. Measuring quality of care in South African clinics and hospitals. (Technical report to chapter 14 of the

South African Health Review 1998). Department of Community Health, University of Witwatersrand; 1998. Available: http://www.hst.org.za/uploads/files/care_98.pdf. Accessed 6 Aug 2010
125. Schneider H, Chabikuli N, Blaauw D, Funani I, Brugha R. Sexually transmitted infections - factors associated with quality of care among private general practitioners. *South African Medical Journal* 2005; **95**:782-785.
126. Ramkissoon A, Kleinschmidt I, Beksinska M, Smit J, Hlazo J, Mabude Z. National Baseline Assessment of Sexually Transmitted Infection and HIV Services in South African Public Sector Health Facilities. Durban: Reproductive Health Research Unit; 2004. Available: http://www.rhru.co.za. Accessed 13 February 2004
127. Reagon G, Irlam J, Levin J. The National Primary Health Care Facilities Survey 2003. Durban: Health Systems Trust; 2004. Available: http://www.hst.org.za/publications/617. Accessed 6 Aug 2010
128. Hoosen A, Nteta C, Moodley J, Sturm A. Diagnosis of bacterial vaginosis and its effect on pregnancy outcome: a preliminary study. *Southern African Journal of Epidemiology and Infection* 1996; **11**:104-106.
129. Govender L, Hoosen AA, Moodley J, Moodley P, Sturm AW. Bacterial vaginosis and associated infections in pregnancy. *International Journal of Gynecology and Obstetrics* 1996; **55**:23-28.
130. Kharsany ABM, Hoosen AA, Moodley J. Bacterial vaginosis and lower genital tract infections in women attending out-patient clinics at a tertiary institution serving a developing community. *Journal of Obstetrics and Gynaecology* 1997; **17**:171-175.
131. Rours GIJG, Verkooyen RP, Ye H, Radebe F, Rothberg AD, Cooper PA*, et al.* Sexually transmitted infections in pregnant urban South African women: socio-economic characteristics and risk factors. *Southern African Journal of Epidemiology and Infection* 2006; **21**:14-19.
132. Sturm AW, Wilkinson D, Ndovela N, Bowen S, Connolly C. Pregnant women as a reservoir of undetected sexually transmitted diseases in rural South Africa: implications for disease control. *American Journal of Public Health* 1998; **88**:1243-1245.
133. Sturm A, Moodley P, Sturm P, Karim F, Khan N. Trends in the prevalence of sexually transmitted infections and HIV in pregnant women in KwaZulu/Natal from 1995 to 2002. *South African AIDS Conference*. Durban, South Africa; 2003.
134. Sturm PD, Connolly C, Khan N, Ebrahim S, Sturm AW. Vaginal tampons as specimen collection device for the molecular diagnosis of non-ulcerative sexually transmitted infections in antenatal clinic attendees. *International Journal of STD and AIDS* 2004; **15**:94-98.
135. Odendaal HJ, Schoeman J, Grové D, de Jager M, Theron GB, Orth H*, et al.* The association between Chlamydia trachomatis genital infection and spontaneous preterm labour. *South African Journal of Obstetrics and Gynaecology* 2006; **12**:146-149.
136. Frohlich JA, Abdool Karim Q, Mashego MM, Sturm AW, Abdool Karim SS. Opportunities for treating sexually transmitted infections and reducing HIV risk in rural South Africa. *Journal of Advanced Nursing* 2007; **60**:377-383.
137. Moodley D, Moodley P, Sebitloane M, Soowamber D, McNaughton-Reyes HL, Groves AK*, et al.* High prevalence and incidence of asymptomatic sexually transmitted infections during pregnancy and postdelivery in KwaZulu Natal, South Africa. *Sexually Transmitted Diseases* 2015; **42**:43-47.

138. Steen R, Vuylsteke B, DeCoito T, Ralepeli S, Fehler G, Conley J*, et al.* Evidence of declining STD prevalence in a South African mining community following a core-group intervention. *Sexually Transmitted Diseases* 2000; **27**:1-8.
139. Williams BG, Taljaard D, Campbell CM, Gouws E, Ndhlovu L, van Dam J*, et al.* Changing patterns of knowledge, reported behaviour and sexually transmitted infections in a South African gold mining community. *AIDS* 2003; **17**:2099-2107.
140. Vickerman P, Terris-Prestholt F, Delany S, Kumaranayake L, Rees H, Watts C. Are targeted HIV prevention activities cost-effective in high prevalence settings? Results from a sexually transmitted infection treatment project for sex workers in Johannesburg, South Africa. *Sexually Transmitted Diseases* 2006; **33**:S122-132.
141. Schneider H, Coetzee DJ, Fehler HG, Bellingan A, Dangor Y, Radebe F*, et al.* Screening for sexually transmitted diseases in rural South African women. *Sexually Transmitted Infections* 1998; **74**:S147-S152.
142. Wilkinson D, Ndovela N, Harrison A, Lurie M, Connolly C, Sturm AW. Family planning services in developing countries: an opportunity to treat asymptomatic and unrecognized genital tract infections? *Genitourinary Medicine* 1997; **73**:558-560.
143. Hoosen AA, Moodley J, Maitin P, Sturm AW. Bacterial vaginosis in symptomatic women attending a gynaecology outpatient clinic. *Southern African Journal of Epidemiology and Infection* 1997; **12**:119-121.
144. Fehler HG, Lyall M, Htun Y, Muiznieks S, Khoza BD, Ballard RC. Genital tract infections among women attending an urban family planning clinic. *Southern African Journal of Epidemiology and Infection* 1998; **13**:79-82.
145. Kleinschmidt I, Rees H, Delany S, Smith D, Dinat N, Nkala B*, et al.* Injectable progestin contraceptive use and risk of HIV infection in a South African family planning cohort. *Contraception* 2007; **75**:461-467.
146. Colvin M, Abdool Karim SS, Connolly C, Hoosen AA, Ntuli N. HIV infection and asymptomatic sexually transmitted infections in a rural South African community. *International Journal of STD and AIDS* 1998; **9**:548-550.
147. Pettifor AE, Kleinschmidt I, Levin J, Rees HV, MacPhail C, Madikizela-Hlongwa L*, et al.* A community-based study to examine the effect of a youth HIV prevention intervention on young people aged 15-24 in South Africa: results of the baseline survey. *Tropical Medicine and International Health* 2005; **10**:971-980.
148. Hurkchand HP, Levine JB, Makuluma H, Molefe N. Measuring the impact of HIV and STIs in a cluster designed household prevalence survey in a coal mining community, eMbalenhle, Mpumalanga, South Africa [Abstract ThPeC7319]. *15th International AIDS Conference*. Bangkok, Thailand; 2004.
149. O'Leary A, Jemmott JB, Jemmott LS, Teitelman A, Anita Heeren G, Ngwane Z*, et al.* Associations between psychosocial factors and incidence of sexually transmitted disease among South african adolescents. *Sexually Transmitted Diseases* 2015; **42**:135-139.
150. Govender S, Theron GB, Odendaal HJ, Chalkley LJ. Prevalence of genital mycoplasmas, ureaplasmas and *chlamydia* in pregnancy. *Journal of Obstetrics and Gynaecology* 2009; **29**:698-701.
151. Auvert B, Males S, Puren A, Taljaard D, Carael M, Williams B. Can highly active antiretroviral therapy reduce the spread of HIV? A study in a township of South Africa. *Journal of Acquired Immune Deficiency Syndromes* 2004; **36**:613-621.
152. Orroth KK, Korenromp EL, White RG, Changalucha J, de Vlas S, Gray RH*, et al.* Comparison of STD prevalences in the Mwanza, Rakai, and Masaka trial populations:

the role of selection bias and diagnostic errors. *Sexually Transmitted Infections* 2003; **79**:98-105.
153. van Doornum GJ, Schouls LM, Pijl A, Cairo I, Buimer M, Bruisten S. Comparison between the LCx Probe system and the COBAS AMPLICOR system for detection of *Chlamydia trachomatis* and *Neisseria gonorrhoeae* infections in patients attending a clinic for treatment of sexually transmitted diseases in Amsterdam, The Netherlands. *Journal of Clinical Microbiology* 2001; **39**:829-835.
154. Cook RL, Hutchison SL, Østergaard L, Braithwaite RS, Ness RB. Systematic review: noninvasive testing for *Chlamydia trachomatis* and *Neisseria gonorrhoeae*. *Annals of Internal Medicine* 2005; **142**:914-925.
155. Fontana C, Favaro M, Cicchetti O, Minelli S, Pistoia ES, Favalli C. Performance of Strand Displacement Amplification assay in the detection of *Chlamydia trachomatis* and *Neisseria gonorrhoeae*. *Japanese Journal of Infectious Diseases* 2005; **58**:283-288.
156. Van Der Pol B, Ferrero DV, Buck-Barrington L, Hook E, Lenderman C, Quinn T, *et al.* Multicenter evaluation of the BDProbeTec ET System for detection of *Chlamydia trachomatis* and *Neisseria gonorrhoeae* in urine specimens, female endocervical swabs, and male urethral swabs. *Journal of Clinical Microbiology* 2001; **39**:1008-1016.
157. Van Dyck E, Ieven M, Pattyn S, Van Damme L, Laga M. Detection of Chlamydia trachomatis and Neisseria gonorrhoeae by enzyme immunoassay, culture, and three nucleic acid amplification tests. *Journal of Clinical Microbiology* 2001; **39**:1751-1756.
158. Chernesky M, Jang D, Gilchrist J, Hatchette T, Poirier A, Flandin JF, *et al.* Head-to-head comparison of second-generation nucleic acid amplification tests for detection of *Chlamydia trachomatis* and *Neisseria gonorrhoeae* on urine samples from female subjects and self-collected vaginal swabs. *Journal of Clinical Microbiology* 2014; **52**:2305-2310.
159. Mushanski LM, Brandt K, Coffin N, Levett PN, Horsman GB, Rank EL. Comparison of the BD Viper System with XTR Technology to the Gen-Probe APTIMA COMBO 2 Assay using the TIGRIS DTS system for the detection of *Chlamydia trachomatis* and *Neisseria gonorrhoeae* in urine specimens. *Sexually Transmitted Diseases* 2012; **39**:514-517.
160. Van Der Pol B, Liesenfeld O, Williams JA, Taylor SN, Lillis RA, Body BA, *et al.* Performance of the cobas CT/NG test compared to the Aptima AC2 and Viper CTQ/GCQ assays for detection of *Chlamydia trachomatis* and *Neisseria gonorrhoeae*. *Journal of Clinical Microbiology* 2012; **50**:2244-2249.
161. Thejls H, Gnarpe J, Gnarpe H, Larsson PG, Platz-Christensen JJ, Ostergaard L, *et al.* Expanded gold standard in the diagnosis of Chlamydia trachomatis in a low prevalence population: diagnostic efficacy of tissue culture, direct immunofluorescence, enzyme immunoassay, PCR and serology. *Genitourinary Medicine* 1994; **70**:300-303.
162. Mills RD, Young A, Cain K, Blair TM, Sitorius MA, Woods GL. Chlamydiazyme plus blocking assay to detect Chlamydia trachomatis in endocervical specimens. *American Journal of Clinical Pathology* 1992; **97**:209-212.
163. Lefebvre J, Laperrière H, Rousseau H, Massé R. Comparison of three techniques for detection of Chlamydia trachomatis in endocervical specimens from asymptomatic women. *Journal of Clinical Microbiology* 1988; **26**:726-731.

164. Haugland S, Thune T, Fosse B, Wentzel-Larsen T, Hjelmevoll SO, Myrmel H. Comparing urine samples and cervical swabs for Chlamydia testing in a female population by means of Strand Displacement Assay (SDA). *BMC Women's Health* 2010; **10**:9.
165. Kapiga S, Kelly C, Weiss S, Daley T, Peterson L, Leburg C*, et al.* Risk factors for incidence of sexually transmitted infections among women in South Africa, Tanzania, and Zambia: results from HPTN 055 study. *Sexually Transmitted Diseases* 2009; **36**:199-206.
166. Peters RP, Dubbink JH, van der Eem L, Verweij SP, Bos ML, Ouburg S*, et al.* Cross-sectional study of genital, rectal, and pharyngeal chlamydia and gonorrhea in women in rural South Africa. *Sexually Transmitted Diseases* 2014; **41**:564-569.
